# The unfolded protein response links tumor aneuploidy to local immune dysregulation

**DOI:** 10.15252/embr.202152509

**Published:** 2021-10-26

**Authors:** Su Xian, Magalie Dosset, Gonzalo Almanza, Stephen Searles, Paras Sahani, T Cameron Waller, Kristen Jepsen, Hannah Carter, Maurizio Zanetti

**Affiliations:** ^1^ Division of Medical Genetics Biostatistics Department of Medicine, Bioinformatics and System Biology Program University of California, San Diego La Jolla CA USA; ^2^ The Laboratory of Immunology Department of Medicine and Moores Cancer Center University of California, San Diego La Jolla CA USA; ^3^ IGM Genomics Center University of California, San Diego La Jolla CA USA

**Keywords:** aneuploidy, macrophages, T cells, tumor immune microenvironment, unfolded protein response, Cancer, Immunology, Translation & Protein Quality

## Abstract

Aneuploidy is a chromosomal abnormality associated with poor prognosis in many cancer types. Here, we tested the hypothesis that the unfolded protein response (UPR) mechanistically links aneuploidy and local immune dysregulation. Using a single somatic copy number alteration (SCNA) score inclusive of whole‐chromosome, chromosome arm, and focal alterations in a pan‐cancer analysis of 9,375 samples in The Cancer Genome Atlas (TCGA) database, we found an inverse correlation with a cytotoxicity (CYT) score across disease stages. Co‐expression patterns of UPR genes changed substantially between SCNA^low^ and SCNA^high^ groups. Pathway activity scores showed increased activity of multiple branches of the UPR in response to aneuploidy. The PERK branch showed the strongest association with a reduced CYT score. The conditioned medium of aneuploid cells transmitted XBP1 splicing and caused IL‐6 and arginase 1 transcription in receiver bone marrow‐derived macrophages and markedly diminished the production of IFN‐γ and granzyme B in activated human T cells. We propose the UPR as a mechanistic link between aneuploidy and immune dysregulation in the tumor microenvironment.

## Graphical abstract

Aneuploidy, the unfolded protein response (UPR) and dysregulated local immunity are a common feature of human solid tumors, however, the relationship between these three variables has not been explored before. This study shows that the UPR links aneuploidy in tumor cells to dysregulation of macrophages and T cells in the tumor microenvironment.

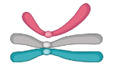

## Introduction

Aneuploidy is the oldest form of chromosomal abnormality identified (Boveri, 2008) and can result from mis‐segregation during anaphase (e.g., spindle assembly, checkpoint defects) (Gordon *et al*, 2012), cell fusion (Migeon *et al*, 1974), or cell‐in‐cell formation (entosis) (Krajcovic *et al*, 2011). In cancer, aneuploidy is part of a broader category of genomic abnormalities called somatic copy number alteration (SCNA; distinguished from germline copy number variations), which are often divided into three categories: whole chromosome, chromosome arm, and focal (Beroukhim *et al*, 2010). Approximately 90% of solid tumors and 50% of blood cancers present some features of aneuploidy (Beroukhim *et al*, 2010; Mitelman *et al*, 2016). Aneuploidy is associated with tumor progression and poor prognosis (Owainati *et al*, 1987; Newburger *et al*, 2013; Hieronymus *et al*, 2018; Stopsack *et al*, 2019), and chromosomally unstable cancer cells exhibit increased multidrug resistance (Duesberg *et al*, 2000). While aneuploidy is usually detrimental to cell viability in healthy tissues leading to negative selection of aneuploid cells, it is paradoxically tolerated in cancer cells (Holland & Cleveland, 2009; Valind *et al*, 2013; Varetti *et al*, 2014) suggesting that it provides selective growth advantage to cancer cells in the hostile tumor microenvironment (Giam & Rancati, 2015).

From an evolutionary perspective, aneuploidy is a source of genetic variation allowing for selection and fitness advantage (Torres *et al*, 2007), but this may also have a functional impact on cells through gene and protein dosage change as demonstrated in yeast and in mammalian cells (Stranger *et al*, 2007; Pavelka *et al*, 2010; Sheltzer *et al*, 2012). In tumors, whole genome duplication facilitates accelerated genome evolution and more aggressive disease (Gao *et al*, 2007; Lopez *et al*, 2020). There is also substantive evidence that overexpression of genes associated with aneuploidy leads to dysregulated proteostasis (Gao *et al*, 2007; Tang & Amon, 2013) and cell stress (Santaguida & Amon, 2015; Zhu *et al*, 2018; Chunduri & Storchova, 2019). This conclusion is supported by studies in yeast (Torres *et al*, 2007; Geiler‐Samerotte *et al*, 2011; Beaupere *et al*, 2018; Tsai *et al*, 2019) and mammalian cells (Senovilla *et al*, 2012; Donnelly *et al*, 2014; Ohashi *et al*, 2015). Importantly, in yeast, quantitative changes in the proteome beyond the buffering capability of the cell cause an unfolded protein response (UPR) (Geiler‐Samerotte *et al*, 2011) and hypo‐osmotic stress (Tsai *et al*, 2019). Congruently, a proteotoxic response is predicted to be a consequence of aneuploidy in cancer cells (Zhu *et al*, 2018; Chunduri & Storchova, 2019) potentially triggering a UPR.

Recent reports showed that tumor aneuploidy correlates with markers of immune evasion (Davoli *et al*, 2017) and reduced number of tumor‐infiltrating leukocytes (Taylor *et al*, 2018) suggesting a connection between aneuploidy and immune surveillance. However, neither study provided a mechanistic explanation for the correlation. Paradoxically, two earlier reports (Senovilla *et al*, 2012; Boileve *et al*, 2013) showed that tetraploid neoplastic cells ostensibly lead to their selective elimination by T cells. Curiously, tissue containing tetraploid cells was also enriched for phosphorylated eIF2α leading to the suggestion that the UPR was involved in a beneficial way. With the issue remaining largely unresolved, we decided to test the hypothesis that an SCNA‐triggered unfolded protein response (UPR) could serve as the link between cancer cell aneuploidy and immune cells (Zanetti, 2017).

The UPR is mediated by three initiator/sensor ER transmembrane molecules: PKR‐like ER kinase (PERK), inositol‐requiring enzyme 1 (IRE1α), and activating transcription factor 6 (ATF6). These are maintained inactive through association with the 78‐kDa glucose‐regulated protein (GRP78) (Schroder & Kaufman, 2005). During ER stress, GRP78 disassociates from each of the three sensor molecules, activating downstream signaling cascades to normalize protein folding and secretion. PERK phosphorylates the translation initiation factor 2 (eIF2α), resulting in global inhibition of translation to reduce ER client proteins (Walter & Ron, 2011). IRE1α auto‐phosphorylates to activate its endonuclease domain, resulting in the cleavage of *XBP1* that generates a spliced XBP1 isoform (XBP1s), which drives the production of various ER chaperones to restore ER homeostasis (Walter & Ron, 2011). XBP1s also binds to the promoter of several pro‐inflammatory cytokine genes (Martinon *et al*, 2010). In addition, under ER stress or forced autophosphorylation, IRE1α's RNase domain can cause endonucleolytic decay of many ER‐localized mRNAs through a phenomenon termed regulated IRE1‐dependent decay (RIDD) (Hollien & Weissman, 2006). ATF6 induces XBP1 and translocates to the Golgi where it is cleaved into its functional form, and acts in parallel with XBP1s to restore ER homeostasis as a transcription factor (Yoshida *et al*, 2001). If these compensatory mechanisms fail, downstream signaling from PERK via transcription factor 4 (ATF4) activates the transcription factor CCAAT–enhancer‐binding protein homologous protein (CHOP) encoded by *DDIT3* to initiate apoptosis (Walter & Ron, 2011).

In cancer cells, the UPR serves as a cell‐autonomous process to restore proteostasis, enable survival, and signal cell growth (Clarke *et al*, 2014; Lee, 2014). However, it can also function cell‐nonautonomously by promoting the release of soluble molecules that target neighboring cells (Mahadevan *et al*, 2011; Rodvold *et al*, 2017). These can increase the fitness and survival of tumor cells (Rodvold *et al*, 2017), impart immunosuppressive and pro‐tumorigenic functions to bone marrow‐derived macrophages and dendritic cells (Mahadevan *et al*, 2011, 2012; Cubillos‐Ruiz *et al*, 2015), and indirectly impair the function of T cells (Mahadevan *et al*, 2012; Song *et al*, 2018).

To test the hypothesis that the UPR may represent the link between aneuploidy and immune dysregulation, we applied statistical methods to UPR gene expression and pathway structures in a pan‐cancer analysis of 9,375 TCGA samples across 32 tumor types using an SCNA score (inclusive of whole‐chromosome, arm, and focal SCNA) and analyzed the effects of aneuploidy‐generated *in vitro* on bone marrow‐derived macrophages and T cells. We show that the UPR is a mechanism by which aneuploidy can disrupt local immunity in cancer contributing to the loss of immune surveillance. Our findings lead to the provisional conclusion that the aneuploidy‐generated UPR is a new variable in the interplay between cancer and immunity.

## Results

### Pan‐cancer distribution of SCNA

**Figure 1:**
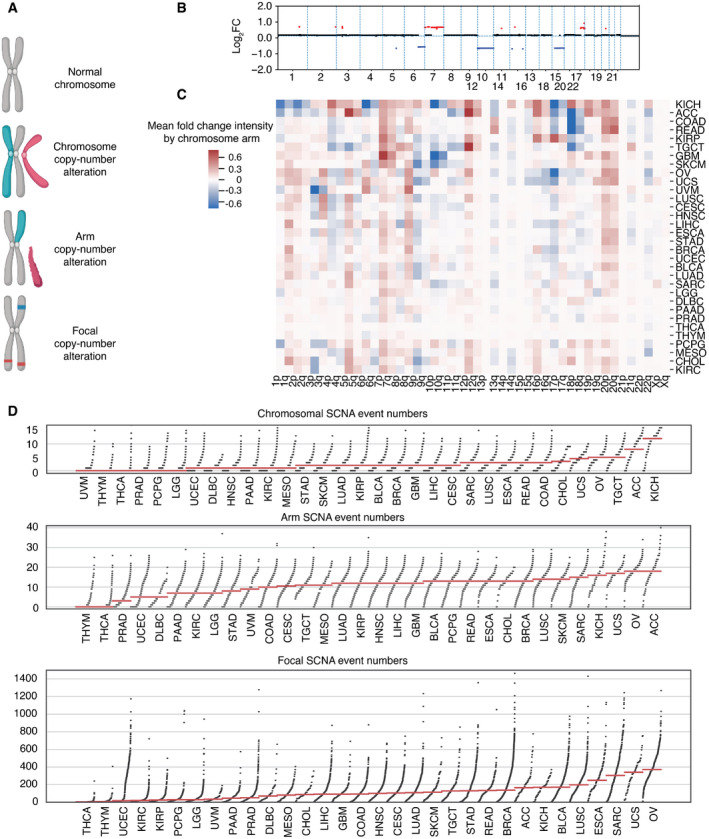
Characterizing aneuploidy across 32 tumor types An illustration on the definition of focal‐, arm‐, and chromosome‐level somatic copy number alterations (SCNAs).Example of somatic copy number alterations detected in a single TCGA sample (TCGA‐02‐0003, GBM) with copy number gain in red and copy number loss in blue. The *x*‐axis represents the chromosomal location, and the *y*‐axis shows the log_2_ fold change in the intensity of the corresponding region relative to diploid.Heatmap showing the average fold change of copy number gain (red) and loss (blue) of chromosomal p and q arm events across 32 tumor types in TCGA.Visualization of the distribution of total SCNA events (loss and gain) by category across tumors grouped by 32 tumor types in TCGA. From top to bottom, plots depict events of chromosomal‐, arm‐, and focal‐level SCNA, respectively.

SCNA has been previously grouped into three categories: whole chromosome, arm, and focal (Beroukhim *et al*, 2010; Davoli *et al*, 2017). Whole‐chromosome copy number alteration refers to a duplication or loss of an entire chromosome (canonical aneuploidy), arm copy number alteration refers to the duplication or loss of an entire chromosome arm, and focal copy number alteration refers to the duplication or loss of a discrete region of the chromosome not spanning the length of the entire chromosome arm (Fig 1A). Arm and focal SCNA have been estimated at 25 and 10% of the genome, respectively (Beroukhim *et al*, 2010; Gordon *et al*, 2012). Here, we studied the three types of SCNA in tumors from The Cancer Genome Atlas (TCGA), quantifying them from segmented SNP array intensity data (see Materials and Methods). An example of a segmented SNP profile of a single tumor harboring all three event categories is shown (Fig 1B).

We first studied the distribution of SCNAs across thirty‐two different tumor types (*n* = 9,375) from TCGA. Recurrent SCNAs affecting specific chromosome arms have been reported, and it is apparent that there is considerable variation in the chromosome arms that are most affected across different tumor types (Fig 1C). For example, 3p arm losses and 3q arm gains were evident in lung squamous cell carcinoma (LUSC) (Fig 1C), consistent with a previous report (Zabarovsky *et al*, 2002). We further compared the number of each SCNA category per tumor across the thirty‐two tumor types (Fig 1D). Some tumor types such as thyroid carcinoma (THCA) and thymoma (THYM) show low SCNA frequency, while others such as ovarian serous adenocarcinoma (OV) and kidney chromophobe cancer (KICH) carry heavy SCNA burdens (Fig 1D).

### Aggregating tumor aneuploidy into a single SCNA score

We first sought to determine whether the three SCNA types could be used and expressed collectively as a single SCNA score. We used pairwise correlation to evaluate the relationship between whole‐chromosome, arm, and focal SCNA categories (raw event count; Spearman correlation, Appendix Fig S1A) and found a strong, positive inter‐category correlation (Spearman *r* = 0.548–0.627). We then derived aggregate scores for each category separately and compared them to a single combined SCNA score (see also Materials and Methods). The combined SCNA score showed consistently high correlation with all three categories considered independently (Spearman *r* = 0.735–0.866) with focal SCNA being the least correlated (Spearman *r* = 0.735) (Appendix Fig S1B–D).

To further assess the combined score, we revisited major genomic correlates of aneuploidy, including mutational burden and TP53 mutation status. Early studies suggested an inverse correlation between the number of non‐synonymous mutations and copy number alterations (Ciriello *et al*, 2013). This was later found to result from a strong inverse correlation between mutation and SCNA in a subset of microsatellite instability high (MSI‐H) tumors (Taylor *et al*, 2018). As reported, microsatellite stable (MSS) tumors showed positive correlation between mutational burden and SCNA arm‐level events (Appendix Fig S2A). The combined SNCA score recapitulates this finding (Appendix Fig S2B).

We also found a positive association between *TP53* mutations and combined SCNA score (Appendix Fig S3A), consistent with the finding of Soto *et al* that *TP53* plays a role in preventing propagation of chromosome segregation errors (Soto *et al*, 2017). We computed *TP53* activity scores using ten *TP53*‐repressed genes (Cancer Genome Atlas Research Network. Electronic address and Cancer Genome Atlas Research, 2017) and found a significant negative correlation between *TP53* activity and SCNA score in seventeen out of thirty‐two cancer types (Appendix Fig S3B). Among these, only THYM showed a significant positive correlation. Across tumors, TP53 activity was negatively correlated with each of the three SCNA categories (Appendix Fig S3C). Our results support the view that inactivation and mutations of *TP53* associate with high SCNA scores in most solid tumors (Zack *et al*, 2013).

### SCNA score negatively correlates with immune‐mediated cytotoxicity

**Figure 2:**
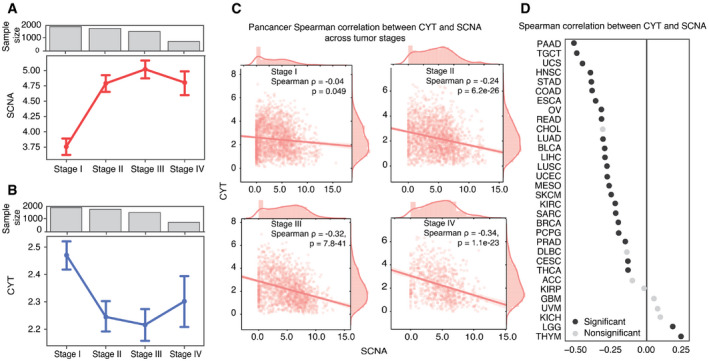
SCNA accumulates as tumor stage progresses and negatively correlates with immune cytolytic activity Mean and 95% confidence interval is shown for SCNA scores among samples at each tumor stage across 6,298 TCGA samples with stage annotation and SCNA score available.Mean and 95% confidence interval for CYT scores among samples at each tumor stage across 6,458 TCGA samples with stage annotation and CYT score available.Pan‐cancer correlation between SCNA score and CYT score with tumors grouped by stage. Spearman rank correlation coefficient and statistical significance (calculated using the student’s *t* distribution with degrees of freedom = *n* – 2) are shown at the top right of each panel.Spearman correlation coefficients linking SCNA and CYT scores across 32 tumor types. Black circles denote significant correlation (FDR < 0.05) after Benjamini–Hochberg multiple testing correction.

In a pan‐cancer analysis of tumors with stage information (*n* = 6,298, 25 tumor types), we found that as tumor stage increased, the single combined SCNA score (aneuploidy score) also increased (Fig 2A). We also measured perforin (*PRF1*) and granzyme A (*GZMA*) gene expression as representations of cytolytic activity (CYT) in tumors (Rooney *et al*, 2015) and found that CYT was inversely correlated with tumor stages across all cancer types (*n* = 6,458, 25 tumor types) (Fig 2B). To account for a potential bias due to differences in stage and SCNA distribution across tumor types (Appendix Fig S4A), we included tumor type as a covariate in an Ordinary Least Squares (OLS) linear regression model. We defined separate models to predict SCNA scores and CYT scores from tumor stage, using Stage I as a baseline for comparison (Table 1). SCNA scores were significantly higher in stages II‐IV relative to stage I (*P* = 1.39e‐09, *P* = 3.77e‐10, *P* = 2.01e‐11) (Table EV1). For CYT, we observed a near significant negative coefficient for Stage II (*P* = 0.075) and a significant negative coefficient for Stage IV (*P* = 9.74e‐5) (Table 1), but no significant reduction in stage III relative to stage I. Nonetheless, we observed a significant inverse correlation between CYT score and SCNA score in all stages (Fig 2C; Table 1).

**Table 1:** Significant accumulation of SCNA correlates with decreasing of CYT with tumor stage progression. An OLS model coefficient showing a significant accumulation of SCNA and decreasing of CYT with increasing tumor stages (*n* = 6,495), including 25 tumor types as a covariate (ACC, BLCA, BRCA, CESC, CHOL, COAD, ESCA, HNSC, KICH, KIRC, KIRP, LIHC, LUAD, LUSC, MESO, OV, PAAD, READ, SKCM, STAD, TGCT, THCA, UCEC, UCS, and UVM).

	SCNA coeff	SCNA *P*‐value	CYT coeff	CYT *P*‐value
Stage II	0.546	1.39e‐09	−0.069	0.075
Stage III	0.566	3.77e‐10	−0.032	0.407
Stage IV	0.791	2.01e‐11	−0.197	9.74e‐05

Further investigating the relationship between SCNA level and CYT score in individual tumor types, we found significant negative correlation between SCNA scores and CYT levels in 23 out of 32 tumor types (Spearman correlation test; Fig 2D). Although we noted correlation between TP53 activity and SCNA score, the tumor type‐specific correlation between SCNA score and CYT was independent of TP53 activity score by partial correlation analysis (Appendix Fig S4B). Surprisingly, thymoma (THYM) and low‐grade glioma (LGG) showed a significant positive correlation (Fig 2C). We note that THYM had lower SCNA scores than other tumor types, and when we restricted analysis to THYM tumors with an SCNA score > 1, there was a trend toward inverse correlation between SCNA score and CYT (*r* = −0.325, *P* = 0.096). In low SCNA THYM tumors, other factors appear to influence CYT levels, which may reflect the association of THYM with autoimmunity (Shelly *et al*, 2011) and altered immune surveillance and potentially promoting alternative mechanisms immune evasion and increased cytotoxic T cells (Hoffacker *et al*, 2000; Bando *et al*, 2017). Trends in LGG and GBM may reflect differences in the tumor microenvironment in the brain; LGG in particular is characterized by low levels of CD8 T‐cell infiltration (Weenink *et al*, 2019). Together, these observations suggest that as most tumors progress, they accumulate SCNAs and evade immunity.

**Figure EV1:**
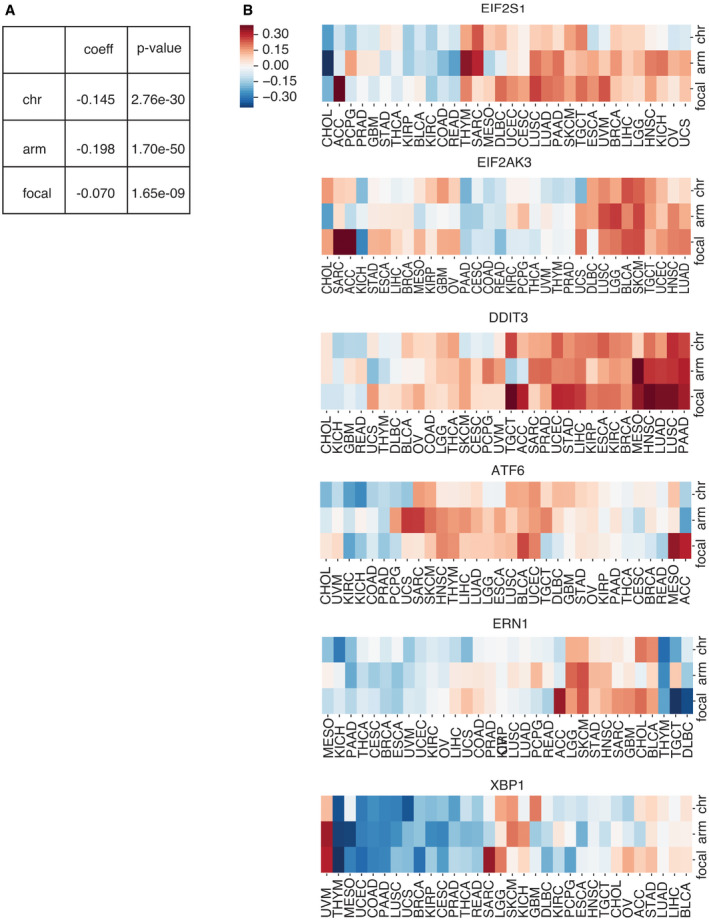
Three categories of SCNA event show similar trends in correlation with CYT score UPR gene expression Coefficients and p‐values of an ordinary least square (OLS) linear model using 3 categories of SCNA to predict CYT score, with tumor type as a covariate.Heatmaps showing correlation between the three categories of SCNA event (rows) and UPR parental genes for 32 tumor types (columns). Color intensity indicates magnitude of Spearman correlation, with red indicating positive and blue indicating negative correlation, respectively.

A previous report suggested that among SCNA categories, whole chromosome‐ and arm‐level event burden are predictive of immune evasion whereas focal event burden is associated with cell cycle (Davoli *et al*, 2017). We found that focal SCNAs also inversely correlate with CYT levels, albeit more weakly than chromosome‐ or arm‐level SCNAs (Fig EV1A) in an OLS model using all three categories of SCNA to predict CYT score and including tumor type as a covariate. This supported the use of the combined SCNA score to analyze the impact of chromosomal abnormalities during tumor progression on immune dysregulation (i.e., decrease cytolytic activity) leading to progressive immune incompetence and immune evasion. In considering the effects of SCNA levels on UPR maintenance of proteostasis, we also did not expect the three SCNA categories to substantially differ from one another. Indeed, they all showed similar Spearman correlation to parental UPR gene expression across all 32 tumor types (Fig EV1B). In light of this, all subsequent analyses were performed using the single SCNA score as a simplified measure of aneuploidy burden.

### UPR gene expression correlates with SCNAs

**Figure 3:**
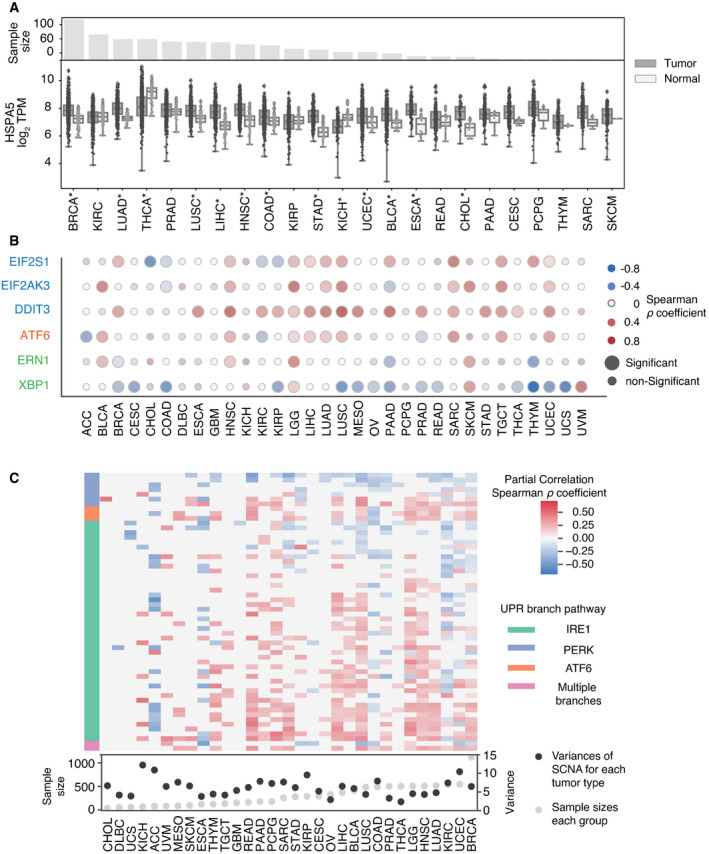
The unfolded protein response is influenced by SCNA levels A boxplot showing log_2_ TPM values of HSPA5 gene expression compared between tumors and matched normal samples across 23 tumor types from TCGA with normal tissue data available. The boxplot indicates the median (central band) and the interquartile range (boxes) of the expression value, and whiskers indicate values that outside of the middle 50% (interquartile). The barplot on the top shows the number of normal tissue samples for each corresponding tumor type. Asterisks indicate significant differences by Student’s *t*‐test after Benjamini–Hochberg multiple testing correction (FDR < 0.05).A heat map showing the Spearman correlation coefficient between the log_2_ TPM expression of three UPR branch pathway parental genes (rows) and SCNA scores across 32 tumor types (columns). Red indicates positive correlation coefficients (*r* > 0), and blue indicates negative correlation coefficients (*r* < 0). Size indicates significance level of correlation. Gene names colored in blue belong to the PERK pathway, orange to the ATF6 pathway, and green to the IRE1 pathway.A heatmap showing the partial correlation between the expression of UPR branch pathway genes with SCNA levels controlling for the effect of the gene copy number change using data from GISTIC2.0. Rows depict genes from the UPR branch pathways from REACTOME, and columns depict the 32 tumor types. Cells are colored in red or blue if the gene showed significant correlation with SCNA level in that tumor type (partial correlation test setting copy numbers for genes as control, after Benjamini–Hochberg multiple hypothesis correction, FDR < 0.05). Color intensity corresponds to the partial correlation coefficient between gene expression and SCNA level. The left side bar indicates pathway membership of the genes. The bottom panel shows the variances of SCNA scores for each tumor type and the number of normal tissue samples available for partial correlation analysis.

The UPR is an adaptive survival mechanism used by mammalian cells in response to environmental perturbations, cell‐autonomous, and cell‐nonautonomous signaling to alleviate the burden of excess client proteins in the ER (Walter & Ron, 2011). To investigate the relation between SCNA and the UPR, we first examined the expression of a few representative genes from each major UPR pathway. We compared gene expression levels for the master regulator of the UPR, heat‐shock protein family A member 5 (*HSPA5*) in tumors, and matched normal tissues. Out of the twenty‐three tumor types with available matched normal samples in TCGA, all except three (THCA, KICH, and KIRP) showed greater *HSPA5* expression in tumors, and thirteen of these showed statistical significance (FDR < 0.05) (Fig 3A). Notably, small sample sizes for matched normal tissues limited the statistical power in a few cancer types: skin cutaneous melanoma (SKCM, *n* = 1), thymoma (THYM, *n* = 2), brain tumors (GBM, LGG, *n* = 0), and pheochromocytoma or paraganglioma (PCPG, *n* = 3). We next evaluated the Spearman correlation between SCNA score and parent genes for the three branches of the UPR (IRE1α, PERK, and ATF6) across all thirty‐two tumor types in TCGA (Fig 3B). Three genes from the PERK pathway (*EIF2S1*, *EIF2AK3*, and *DDIT3*) showed a positive correlation with the SCNA score across almost every tumor type. *ATF6* also showed a mild positive correlation with SCNA scores across the majority of tumor types (Fig 3B). In contrast, *ERN1* (the gene coding for IRE1α) showed no consistent correlation, and *XBP1* had a mild negative correlation with SCNA score (Fig 3B). This analysis of transcriptional regulation of sensor genes suggests that SCNA levels correlate with activation of UPR branch pathways, mainly the PERK pathway. A positive correlation with *ATF6* is not entirely surprising given its role in targeting stress response genes to cope with a greater client protein burden resulting from SCNAs and facilitating tolerance to chronic stress (Wu *et al*, 2007). On the other hand, the lack of a positive correlation with *ERN1* motivated further analysis given that this pathway has been implicated in tumor survival (Logue *et al*, 2018; Xie *et al*, 2018), macrophage polarization (Batista *et al*, 2020), and T‐cell dysregulation (Song *et al*, 2018).

Some UPR activity, and IRE1α activity in particular, is regulated by post‐translational modifications which may not be reflected in expression levels of UPR branch pathway parent genes. Based on this reasoning, we performed an analysis of genes downstream of each of the three main branches of the UPR, assuming that they would collectively be more indicative of an association with SCNA levels than the parent genes. We first collected gene sets for the IRE1α, XBP1S, PERK, and ATF6 pathways from REACTOME, a curated database of biological pathways (Jassal *et al*, 2020) (Table EV1). These gene sets include members of the branch pathways, but also downstream effector genes that reflect branch pathway activities. We then compared the expression of these genes in each UPR branch in tumor samples with SCNA score using partial correlation analysis to account for contributions of amplifications or deletions affecting each gene (Fig 3C). Of note, inadequate coverage of samples and insufficient variation in SCNA levels posed limitations in this analysis (Appendix Fig S5). For example, CHOL, DLBC, UCS, KICH, ACC, and MESO all have particularly low numbers of samples in each group (*n* < 85, with median sample size = 267), while GBM, OV, CESC, and THCA all have little variation in SCNA levels (variance < 5.48, with mean variance among all tumor types = 6.24). Despite this, we found that over half of the thirty‐two tumor types showed significant correlation between SCNA score and the expression of the majority of downstream genes in all three UPR branch pathways (Fig 3C). Collectively, this broad analysis shows that SCNA is associated with altered gene expression of each of the three branches of the UPR.

### Changes in differential co‐expression of UPR genes between SCNA^low^ and SCNA^high^ tumors

**Figure EV2:**
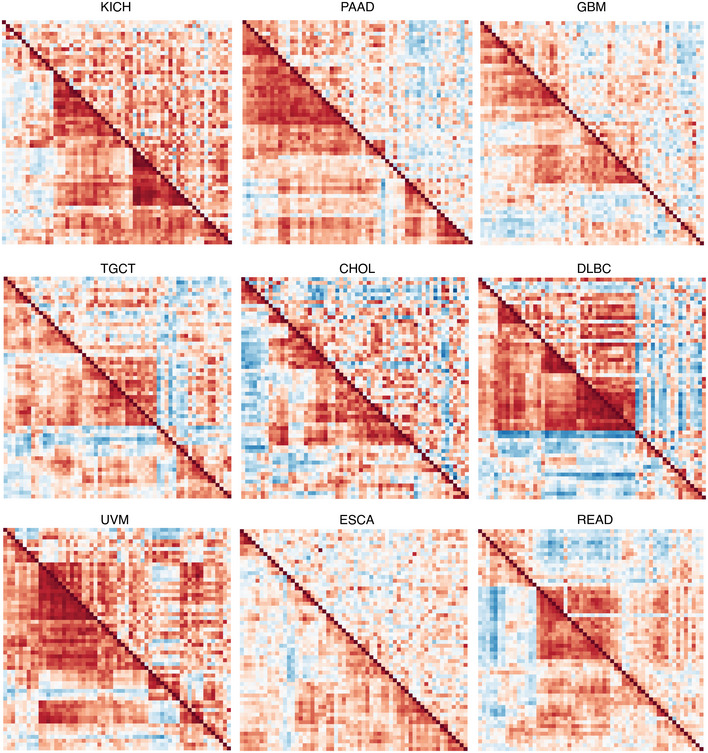
Heatmaps showing UPR gene co‐expression patterns for high and low SCNA groups for nine tumor types Bottom left triangles represent the co‐expression of UPR genes in the SCNA^low^ group, and top right triangles represent the co‐expression of UPR genes in the SCNA^high^ group.

**Figure 4:**
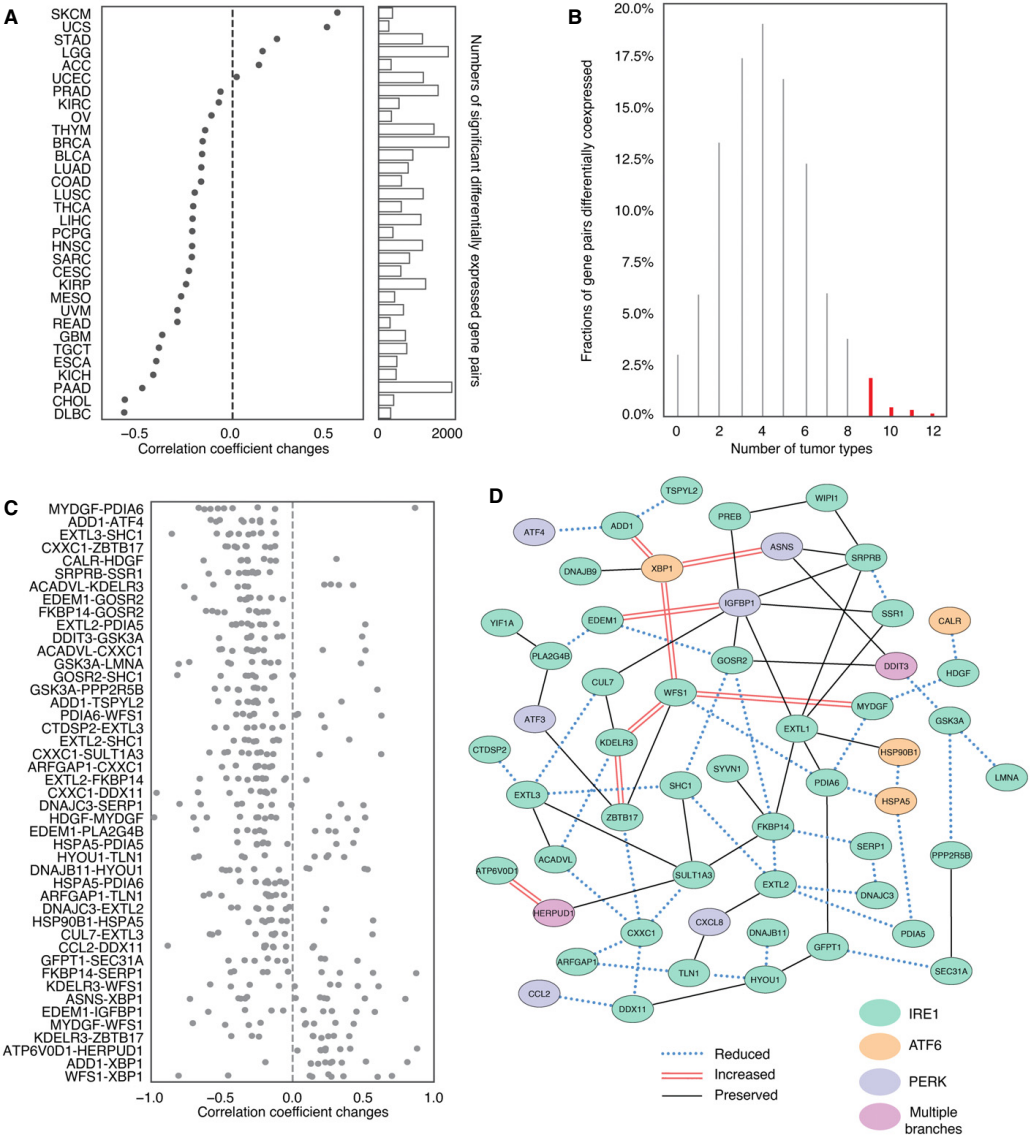
Co‐expression analysis of UPR genes comparing low and high SCNA tumors A strip plot summarizing differences in co‐expression (*x*‐axis) of pairwise combinations of 58 UPR genes (*n* = 3,364) between low and high SCNA groups across 32 tumor types (*y*‐axis) as quantified by the change in Spearman correlation coefficient. The side bar indicates the number of gene pairs with significant co‐expression change relative to a null distribution obtained from 1,000 permutations of SCNA status (see Materials and Methods for detail).A histogram showing the percentage of gene pairs (*y*‐axis; *n* = 3,364) that have significant co‐expression change according to the number of types (*x*‐axis) in which each gene pair was significant. Colored bars indicate the 2.68% of gene pairs (*n* = 45) that were significant in at least nine tumor types and that were selected for more in‐depth analysis.Change in Spearman correlation coefficient between SCNA high and low conditions for 45 gene pairs with significant co‐expression changes across more than nine tumor types (*n* = 45). Each point indicates the difference in correlation for one tumor type where the gene pair was significant.Network plot showing top UPR gene pairs with reduced, preserved or augmented co‐expression. Each node represents a UPR gene, and each edge represents a co‐expression relationship between a gene pair. Red double line edges depict increased co‐expression in SCNA^high^ tumors compared with SCNA^low^. Solid lines depict preserved co‐expression between gene pairs, and blue dotted lines depict reduced co‐expression between gene pairs. Node colors represent the UPR branch pathway membership of genes, with green representing the IRE1α pathway, blue representing the PERK pathway, orange representing the ATF6 pathway, and purple representing membership in more than one branch pathway.

Next, we considered that UPR branch pathway activities themselves could be directly or indirectly affected by SCNAs. Because signaling requires the coordinated activity of multiple proteins, genes within pathways are often more highly co‐expressed (Wolfe *et al*, 2005; Komili & Silver, 2008). Therefore, to assess the impact of SCNA levels on UPR signaling, we evaluated the differential co‐expression of all UPR genes in low and high SCNA groups across tumor types. We divided samples into SCNA^low^ and SCNA^high^ groups using the 30^th^ and 70^th^ percentiles for each tumor type and assessed differences in the pairwise correlation coefficients for all UPR genes between these two groups. We found that almost universally the co‐expression patterns of UPR genes were visibly different between SCNA^low^ and SCNA^high^ groups (Fig EV2), with most tumor types showing less co‐expression in the SCNA^high^ compared with the SCNA^low^ group (Figs 4 and EV2) consistent with general perturbation of the transcriptome by SCNAs. In general, the SCNA^high^ condition showed loss of coordination of UPR genes relative to the SCNA^low^ condition (Fig EV2). The strongest effects were observed in PAAD, GBM, KICH, CHOL, UVM, and ESCA.

**Figure EV3:**
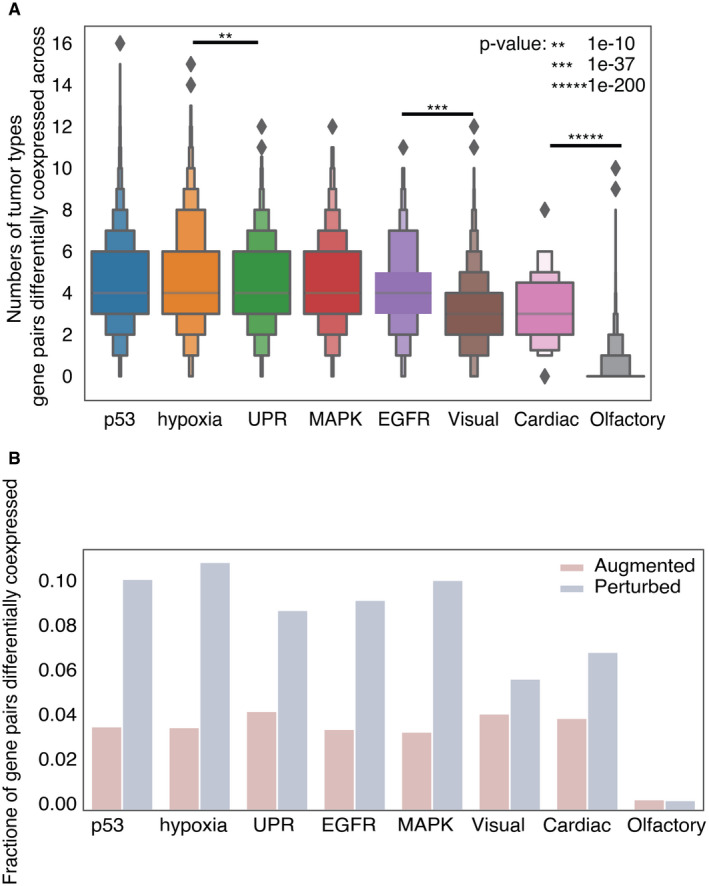
Comparison of differential co‐expression of gene pairs under high and low SCNA conditions across the UPR and seven control pathways Gene pairs are more consistently perturbed across tumor types for the UPR pathway and cancer pathways (*n* = 3) than for control pathways (*n* = 3). The *x*‐axis represents the numbers of tumor types that where a given gene pair was significantly differentially co‐expressed. The *y*‐axis represents the specific pathway each gene pairs belongs to. Statistical significance was calculated using the student’s *t*‐test.UPR and cancer‐associated pathways show more perturbed gene pairs than non‐cancer control pathways.

We speculated that in the SCNA^high^ setting, the oncogenic effects of UPR would be preserved or amplified while tumor‐suppressive aspects would be reduced. Therefore, we assessed whether loss of coordination of gene expression under SCNA^high^ conditions appeared random by comparing to permuted data. Interestingly, forty‐five gene pairs showed a significant propensity to co‐expression change (permutation‐based FDR <0.05) in at least nine tumor types (Fig 4B). To aid interpretation, we repeated this analysis for three oncogenic pathways (TP53, EGFR, and MAPK) and three pathways selected for lack of association with tumorigenesis (olfaction, cardiac conduction, and visual phototransduction). We noted that the fraction of gene pairs perturbed in association with higher SCNA levels was consistently higher in oncogenic as compared with control pathways (Fig EV3A). Perturbations were also observed more consistently across multiple cancer types for oncogenic versus control pathways (Fig EV3B). We used the control pathways to estimate an empirical false discovery rate of 0.0995 (˜10%) for detection of recurrently perturbed genes. This may be conservative as we found literature evidence that the genes in the top perturbed gene pair of the visual phototransduction control pathway, METAP2‐FNTA, play roles in oncogenic progression in rhabdomyosarcoma (Nielsen *et al*, 2012) and pancreatic endocrine tumors (Larghi *et al*, 2012), respectively.

Among consistently differentially co‐expressed UPR gene pairs, the co‐expression changes were predominately negative (*n* = 37), suggesting a pattern of loss of coordination (Fig 4C). Some genes were included in multiple perturbed pairs. Among highly perturbed gene pairs across multiple tumor types, we noted *CXXC1, HSPA5, GSK3A, SERP1, PDIA6, FKBP14,* and *SCH1*, which showed multiple co‐expression changes. Most of these genes (*HSPA5*, *CXXC1*, *SERP1*, *SCH1, PDIA6*) encode proteins that confer resistance to various forms of stress. GSK3A additionally functions as an oncogene by stabilizing β‐catenin and promoting self‐renewal. These genes have been associated with unfavorable prognosis in a cancer type‐related manner (Appendix Fig S6) (Uhlen *et al*, 2017). On the other hand, co‐expression of some gene pairs was preserved across all tumor types despite increased SCNA. We identified 34 gene pairs involving 35 genes that showed significant correlation in all tumor types (FDR < 0.05; Table EV2). Gene ontology analysis of genes with reduced, augmented, or preserved co‐expression suggested that genes with preserved or augmented co‐expression, but not those with perturbed co‐expression, were associated with negative regulation of apoptosis (Appendix Fig S7, Table EV3, GO:1902236, GO:2001243, GO:2001234, GO:0043066).

**Figure EV4:**
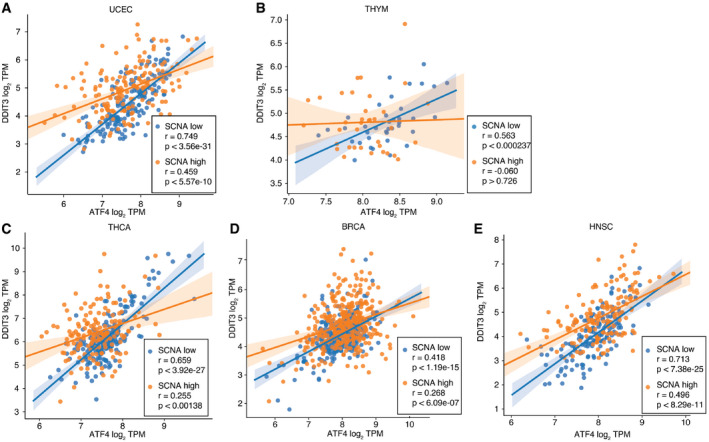
ATF4 and DDIT3 gene loss of coordination in four tumor types A–EScatter plots showing the log_2_ TPM expression level of ATF4 (*x*‐axis) and DDIT3 (*y*‐axis) in SCNA^low^ (blue) and SCNA^high^ (orange) conditions for tumors from four different tumor types: (A) UCEC—endometrial cancer; (B) THYM—thymoma; (C) THCA—thyroid cancer; (D) BRCA—breast adenocarcinoma; (E) HNSC—head and neck squamous cell carcinoma. Regression lines indicate the strength of correlation between the expression of the two genes. Spearman correlation coefficients and associated p‐values are provided.

**Figure EV5:**
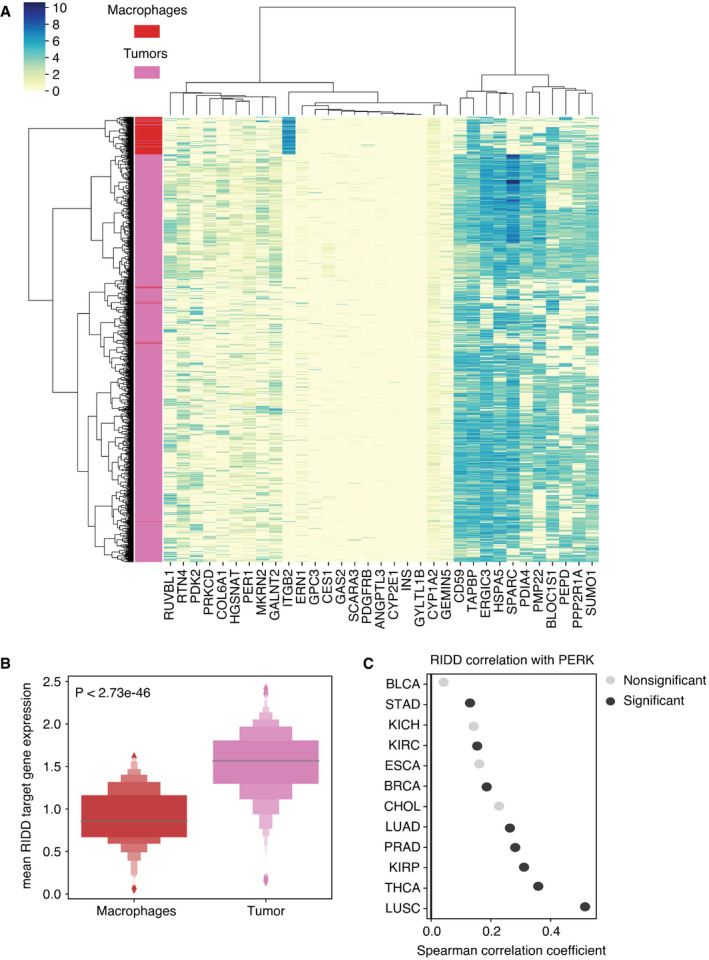
Analysis of RIDD target gene expression in single‐cell data showed reduced expression in tumor‐associated macrophages compared with tumor cells Heatmap showing the unsupervised clustering of 1,257 tumor cells and 119 macrophages (rows) according to expression in log_2_(TPM/10 + 1) of 33 RIDD target genes (columns). The left sidebar indicates cell type: red—macrophages; pink—tumor cells.Boxplots showing the distribution of mean RIDD target gene expression in macrophages (blue) and tumor cells (orange), *P* = 2.73e‐46, Wilcoxon rank‐sum test. ITGB2 and TAPBP were excluded as their behavior is counter to regulation by RIDD.Spearman correlation coefficients linking PERK pathway score and RIDD activity score for 12 tumor types for which both pathway scores could be calculated. Black color indicates correlations that were statistically significant after multiple hypothesis testing correction using the Benjamini–Hochberg procedure (FDR < 0.05). Gray dots represent non‐significant cases.

We summarized perturbed, augmented, and preserved gene co‐expression relationships with a network (Fig 4D). This highlights more preserved relationships among genes involved in core activities of the ER such as cellular metabolism and co‐translational translocation to the ER (IGFBP1‐SRPRB, IGFBP1‐SRR1) and pairs with at least one member involved in protein trafficking (PREB‐WIPI1, KDELR3‐CUL7, GOSR2‐IGFBP1, SYVN1‐FKBP14, YIF1A‐PLA2G4B). We note that the relationship between ATF4 and DDIT3, while not consistently perturbed—ATF4 and DDIT3 co‐expression was only significantly perturbed in 5 tumor types (Fig EV4, EV5; UCEC, THYM, THCA, BRCA, and HNSC, respectively)—was also not preserved. Interestingly *DDIT3* co‐expression with *GOSR2* (protein transport) and *ASNS* (asparagine synthetase) remained coordinated, suggesting that some less known aspects of DDIT3 activity may benefit tumor cells. DDIT3 is frequently thought of as the major executioner of apoptosis downstream of irrecoverable ER stress, but it may be required for other functions, for example, the induction of the pro‐inflammatory/tumorigenic cytokine IL‐23 (Goodall *et al*, 2010). While a deeper analysis of the coordinated activities of UPR proteins is merited, overall, this pattern is consistent with promoting positive aspects of UPR signaling. These may include regulation of metabolism, transport, and bioenergetics, favoring cell survival while diminishing effects disadvantageous to the cell such apoptosis. Preservation of the UPR in SCNA^high^ tumors argues therefore for an active stress response to proteostasis. Since UPR signaling is known to affect immune cells, we next interrogated the UPR as the link between SCNA and reduced CYT.

### UPR activity links SCNA and CYT

Given that overall the UPR is activated in tumors relative to normal tissues (Fig 3A and C), but increasing SCNA levels make expression‐based assessment of pathway activity from individual genes ambiguous, we developed a strategy to measure pathway activation from the combined effects of multiple genes. To establish a gene expression‐based method to assess UPR branch pathway activity in tumors, we adapted the pathway measurement method of Schubert *et al* (2018) applying a regression model to assign coefficients for genes within pathways and then deriving aggregate pathway activation scores by matrix multiplication. We used a Lasso regression model to remove redundant genes from each pathway, to avoid overfitting and capture dominant differences (Appendix Fig S8, Table EV4). We applied this method using gene sets from REACTOME (58 genes) (Jassal *et al*, 2020) as previously described, further distinguishing IRE1α into its known functions, XBP1 splicing and RIDD, as these are non‐overlapping activities. Our final scores represent differential activity in each UPR branch based on contrasting expression of genes in tumors and matched normal tissues (*n* = 23). Due to the limitation imposed by lack of matched normal tissues, we were only able to acquire pathway scores for twenty‐three tumor types (see Materials and Methods).

**Figure 5:**
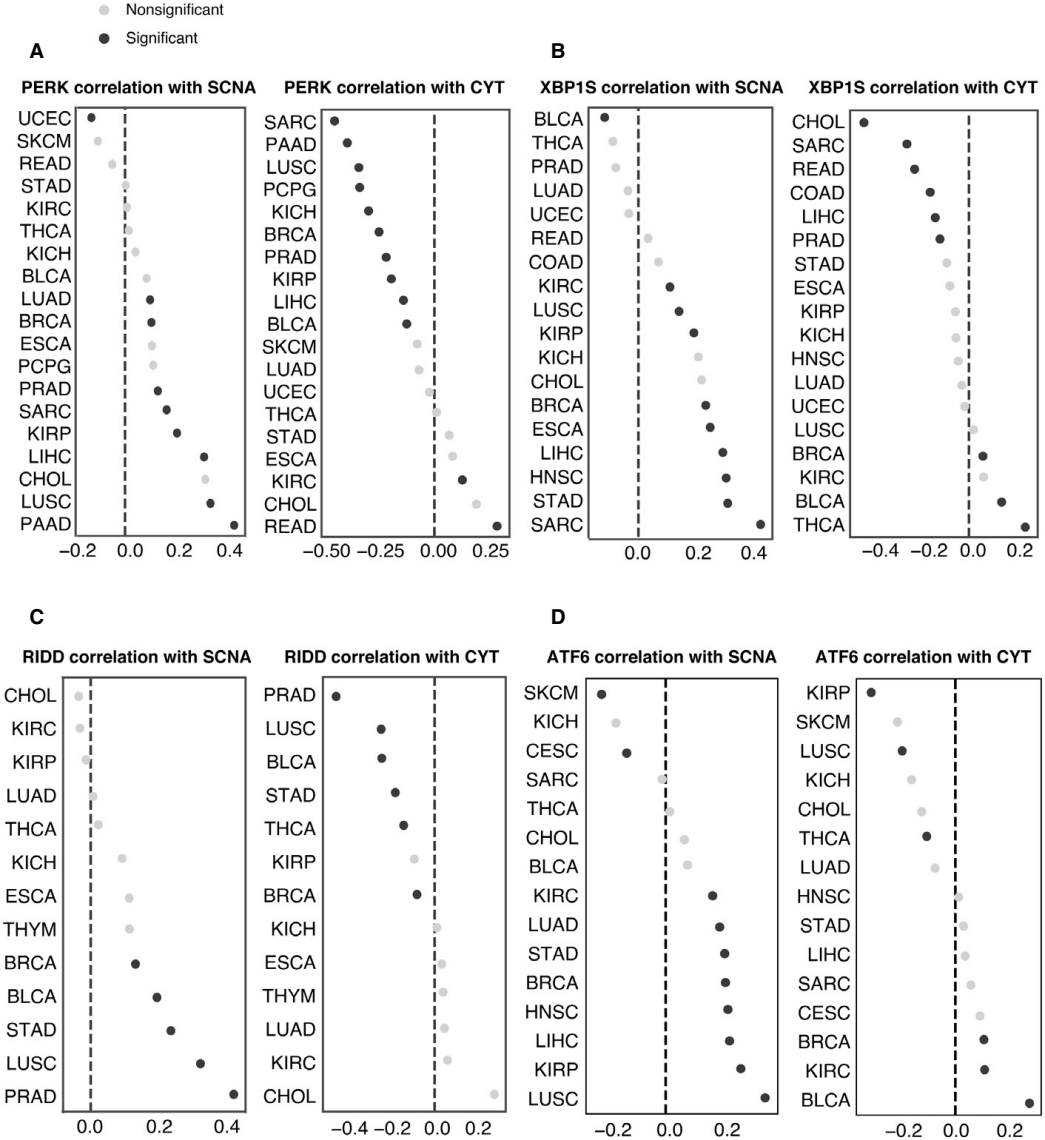
RIDD and PERK pathway activity scores show an inverse correlation with CYT score Spearman correlation coefficients (x‐axis) linking PERK pathway score with SCNA score (left), and CYT score (right) across 19 tumor types for which PERK pathway scores could be calculated.Spearman correlation coefficients (*x*‐axis) linking XBP1S pathway score with SCNA scores (left), and CYT score (right) across 18 tumor types for which XBP1S pathway scores could be calculated.Spearman correlation coefficients (*x*‐axis) linking RIDD activity score with SCNA score(left), and CYT score (right) across 13 tumor types for which RIDD activity scores could be calculated.Spearman correlation coefficients (*x*‐axis) linking ATF6 pathway score with SCNA score (left), and CYT score (right) across 15 tumor types for which ATF6 pathway scores could be calculated.

Among UPR branch pathways, we found that the PERK pathway had a strong inverse correlation with CYT (Fig 5A, *n* = 19 tumor types with non‐zero pathway score). We then interrogated the IRE1α pathway by looking at XBP1, the canonical target of IRE1α endonuclease activity. The pathway score for spliced XBP1 (XBP1s) trended toward a mild negative correlation with CYT score and a positive correlation with SCNA (Fig 5B, *n* = 18 tumor types with non‐zero pathway score).

The IRE1α pathway has a second downstream activity besides XBP1 splicing: the regulated IRE1α‐dependent decay of mRNA or RIDD (Hollien *et al*, 2009). Because of this functional duality, we decided to fully explore the signal from IRE1α by extracting thirty‐three RIDD target genes (Maurel *et al*, 2014). The RIDD pathway score was both significantly positively correlated with SCNA and negatively correlated with CYT in five tumor types (BRCA, BLCA, STAD, LUSC, and PRAD) (Fig 5C). We observed largely positive correlation between ATF6 and SCNA level but little correlation with CYT score (Fig 5D). Collectively, our analysis suggests that both PERK and IRE1α (through RIDD) are associated with mechanisms of immune evasion in the tumor microenvironment.

We next evaluated the three UPR pathways relative to the effect of SCNA on the CYT score controlling for tumor type and purity in a single model. Tumor purity was included as a possible confounding factor since SCNA scores could be underestimated for lower purity tumors, and higher levels of immune infiltrate could inflate CYT scores. We obtained IHC‐based estimates of tumor purity for TCGA from (Aran *et al*, 2015). We then applied an OLS linear model to evaluate the relative contributions of SCNA together with all UPR branches and IHC score in predicting CYT, including tumor type as a covariate, and limiting analysis to samples from the sixteen tumor types for which IHC scores were available. We found that SCNA had a highly significant negative coefficient (coefficient = −0.302, *P* < 1.16e‐118) in predicting CYT (Table 2). Similar to SCNA, both RIDD and PERK had negative coefficients (RIDD coefficient = −0.038, *P* > 0.434; PERK coefficient = −0.278 *P* < 2.11e‐10), though only PERK was significant, suggesting that these UPR branches are associated with reduced immune activity. In contrast, ATF6 had a positive effect on CYT levels (coefficient = 0.252, *P* < 8.14e‐6) and XBP1s were not associated with CYT scores (coefficient = 0.058, *P* > 0.194). In a model without IHC, RIDD reached statistical significance (coefficient = −0.146, *P* < 7.70e‐3, Table 3), pointing to infiltrating immune cells as the likely source of CYT suppressive RIDD signaling. XBP1 activity remained unassociated (coefficient = 0.083, *P* > 0.093).

**Table 2:** Coefficient of an OLS regression model using UPR pathway scores and SCNA scores. Coefficient of an OLS regression model using 4 UPR pathway scores and SCNA scores, including 16 tumor types (ACC, BLCA, BRCA, CESC, COAD, GBM, HNSC, KICH, KIRC, KIRP, LGG, LIHC, LUAD, LUSC, OV, PRAD, READ, SKCM, THCA, UCEC, and UCS) with IHC data available as covariate to predict CYT, *n* = 7,802.

	Coeff	*P*‐value	95% CI
SCNA	−0.302	1.16e‐118	−0.327, −0.277
XBP1S	0.058	0.194	−0.029, 0.145
PERK	−0.278	2.11e‐10	−0.363, −0.192
ATF6	0.252	8.14e‐06	0.141, 0.362
RIDD	−0.038	0.434	−0.134, 0.057
IHC	−0.092	2.09e‐16	−0.114, −0.070

**Table 3:** Regression model coefficients, *P*‐values, and 95% confidence intervals for a pan‐cancer OLS model linking SCNA and four UPR pathway activity scores to CYT levels, including tumor type as a covariate, but excluding IHC level.

	Coeff	*P*‐value	95% CI
SCNA	−0.300	8.92e‐120	−0.324, −0.275
XBP1S	0.083	0.093	−0.014, 0.180
PERK	−0.382	3.72e‐13	−0.485, −0.279
ATF6	0.259	8.67e‐05	0.130, 0.389
RIDD	−0.146	7.70e‐03	−0.253, −0.039

Lack of correlation with XBP1 in tumor cells is not surprising given the demonstration that XBP1 in immune cells (dendritic cells and T cells) plays a tumor‐promoting role (Cubillos‐Ruiz *et al*, 2017), hence highlighting the relevance of cell types and lineages in defining the role of UPR branches in the tumor microenvironment. Remarkably, RIDD gene expression was more suppressed in infiltrating macrophages than in tumor cells in single‐cell RNA expression data from (Tirosh *et al*, 2016) (Fig EV5A and B), in agreement with recent findings in murine macrophages (Batista *et al*, 2020). Collectively, the fact that RIDD and PERK have a similar relationship to CYT is not surprising since RIDD activity was shown to be PERK dependent (Moore & Hollien, 2015). To assess this dependent relationship, we evaluated the Spearman correlation between the PERK pathway and RIDD across the same sixteen tumor types. We found significant positive correlation in eight out of twelve tumor types (Fig EV5C) where both RIDD and PERK pathway scores were available, consistent with the possibility of functional interdependence (Moore & Hollien, 2015). Thus, we conclude that among the UPR branch pathways, PERK and RIDD likely exert a negative effect on immune cells in the tumor microenvironment.

### Experimental aneuploidy induces the UPR

**Figure 6:**
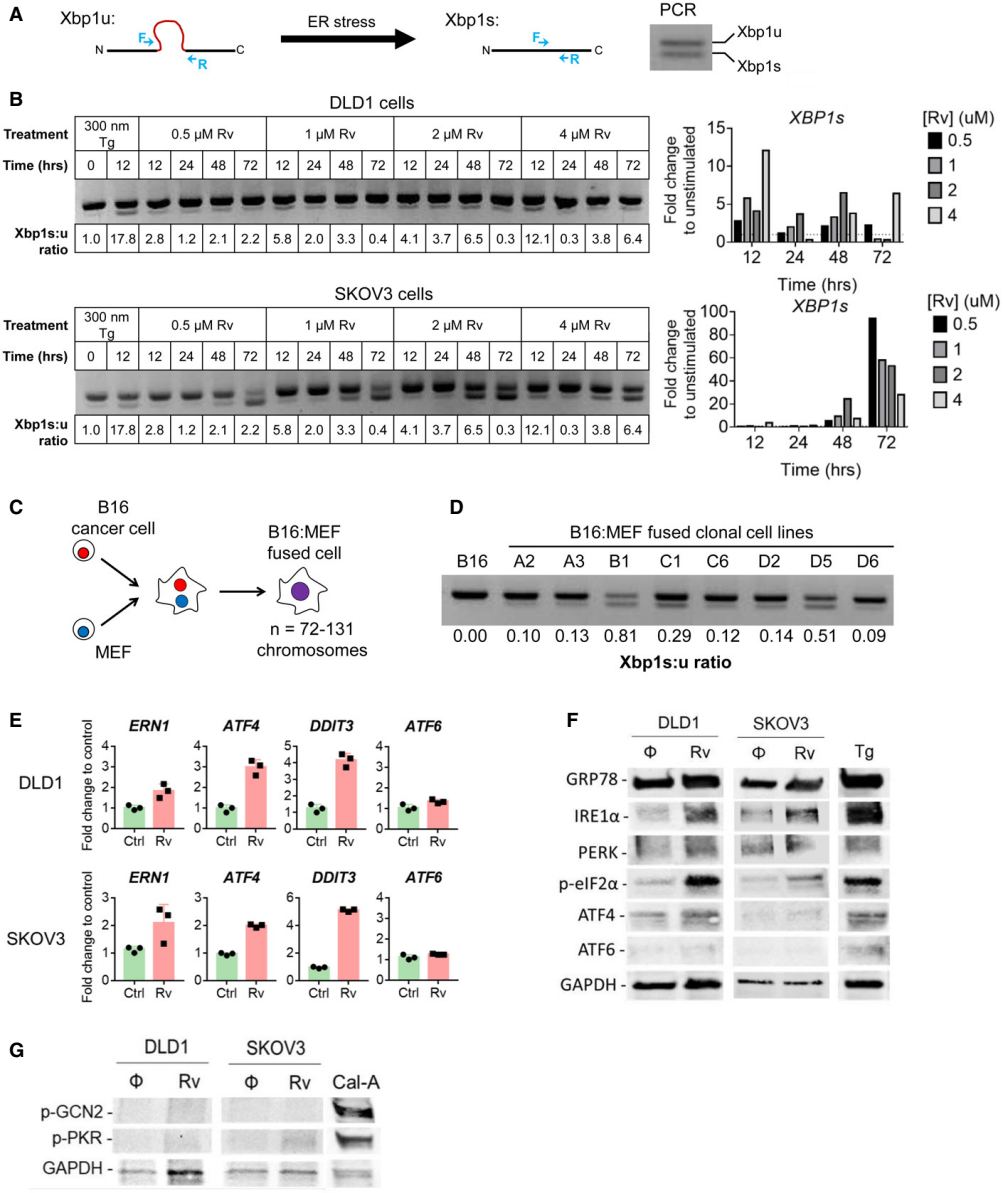
Experimental aneuploidy triggers activation of the UPR pathways in cancer cells Schematic representation of PCR‐based analysis of Xbp1 splicing. During conditions of ER stress, a 26 base pair fragment is spliced from Xbp1 mRNA. Forward and reverse PCR primers (shown in blue) were designed to span the splice site. PCR amplification distinguishes between unspliced (Xbp1‐u, upper band) and spliced (Xbp1‐s, lower band) Xbp1 mRNA. To quantify ER stress, a ratio of spliced: unspliced Xbp1 was calculated.Analysis and quantification of Xbp1 splicing in DLD1 and SKOV3 cells treated with varying concentrations of reversine (Rv) for 12, 24, 48, and 72 h. Thapsigargin (Tg) is shown as positive control.Schematic representation depicting cell–cell fusion between a B16 melanoma cell and mouse embryonic fibroblasts (MEF).Xbp1 splicing in B16 melanoma cells and eight B16:MEF fused clonal cell lines.Detection of mRNA expression (by RT–PCR) of UPR‐associated genes (*ERN1, AF4, DDIT3 and ATF6*) in tumor cells treated with reversine (Rv) at 4 µM (DLD1 cells) or 0.5 µM (SKOV3 cells) for 6 h. Data points refer to triplicate samples collected at the same time point, run in duplicate, and expressed as means ± SD.Western blot analysis of the activation of IRE1, PERK, and ATF branches of the UPR in DLD1 and SKOV3 cells treated or not with Rv at 4 µM for 24H (DLD1 cells) or 0.5 µM for 3 days (SKOV3 cells). Thapsigargin (Tg) was used as positive control.Western blot analysis of phosphorylation of GCN2 and PKR defining the integrated stress response (ISR) in DLD1 and SKOV3 cells treated or not with Rv as in F). Cells starved for 24 h then treated for 30 min with Calyculin‐A (Cal‐A) were used as positive control.

A mechanistic link between aneuploidy and the UPR in cancer cells was sought using reversine (Rv), a small molecule known to induce aneuploidy through inhibition of the mitotic spindle (Santaguida *et al*, 2015, 2017). To maximize the effect of Rv, we used two human cancer cell lines reported to be “quasi‐diploid”: DLD1 (colon cancer; 2*n* = 46) and SKOV3 (ovarian cancer; 2*n* = 46) (Buick *et al*, 1985a; Knutsen *et al*, 2010). Digital karyotyping was performed as previously described (D'Antonio *et al*, 2017). In untreated DLD1 cells that present trisomy on 11p, Rv treatment promoted additional abnormalities (trisomy of 11q and of chromosome 20) (Appendix Fig S9). We treated semi‐confluent cells with varying concentrations of Rv for up to 72 h and measured *XBP1* mRNA splicing by PCR as an indicator of an ER stress response (Fig 6A). After treatment, both cell lines showed demonstrable ER stress with varying kinetics. A quantification of *XBP1* splicing revealed that maximal effect in DLD1 occurs at 12 h while in SKOV3 the effect is maximal at 72 h (Fig 6B). This shows that both cell lines respond to short‐term Rv treatment activating the UPR, albeit with slightly different kinetics.

To determine whether the effect of Rv on *XBP1* splicing was sustained, we performed a second experiment with “long‐term” Rv exposure (14 days) followed by a wash‐out period (no Rv) for up to 3 weeks (Appendix Fig S10A). We found that both DLD1 and SKOV3 cells had a sustained ER stress response for up to 16 days after Rv removal; by day 21 *XBP1* splicing was no longer detected (Appendix Fig S10B). Thus, prolonged treatment with Rv induces a UPR lasting several weeks after Rv removal linking aneuploidy and UPR both in acute and chronic conditions.

We sought independent validation by testing a panel of eight clonal cell lines derived through cell–cell fusion between B16 melanoma cells and mouse embryonic fibroblasts (MEF) (Searles *et al*, 2018) (Fig 6C). The chromosome numbers in these fused cell lines range from 72 to 131 (Searles *et al*, 2018). We tested *Xbp1* splicing in each of the fused clones at baseline and compared it to the parental B16 cell line to see whether fusion‐driven aneuploidy induces the UPR. All (8/8) fused cell lines had higher amounts of *Xbp1* spliced isoform compared with unfused B16 cells (Fig 6D). Thus, two independent models of experimental aneuploidy—Rv treatment and cell–cell fusion—both point to a mechanistic link between aneuploidy and UPR induction.

We performed an analysis of the three branches of the UPR by PCR and Western blotting in DLD1 and SKOV3 cells treated with Rv to determine whether aneuploidy triggers a global UPR. We noted an upregulation of GRP78, the master regulator of the UPR, and CHOP (*DDIT3*). Overall, both the IRE1α and the PERK branches were activated (Fig 6E and F), with phosphorylation of eIF2α downstream of PERK being clearly discernable. We did not detect transcription or translation of ATF6.

Because phosphorylation of eIF2α at Ser^51^ is a convergent regulatory hub of both the UPR and the integrated stress response (ISR), we asked the question as to whether aneuploidy also activates the ISR. The four members of the ISR family include PERK, the double‐stranded RNA‐dependent protein kinase (PKR), the general control non‐repressible 2 (GCN2), and the heme‐regulated eIF2α kinase (HRI) (Reis *et al*, 2012). Although all four eIF2α kinases share extensive homology in their kinase catalytic domains, each responds to distinct environmental and physiological stresses to reflect their unique regulatory mechanisms (Reis *et al*, 2012). Specifically, PKR responds to dsRNA during viral infections and GCN2 responds to amino acid deprivation and glucose deprivation. As shown in Fig 6G, neither kinase was phosphorylated in Rv‐treated DLD1 cells and only a faint p‐PKR band was observed in SKOV3 cells. Treatment with Calyculin‐A, protein phosphatase inhibitor, served as a positive control. Collectively, these results suggest that aneuploidy induced by short‐term Rv treatment mainly drives eIF2α phosphorylation via canonical UPR.

### Aneuploid cells polarize bone marrow‐derived macrophages and negatively affect T‐cell activation

**Figure 7:**
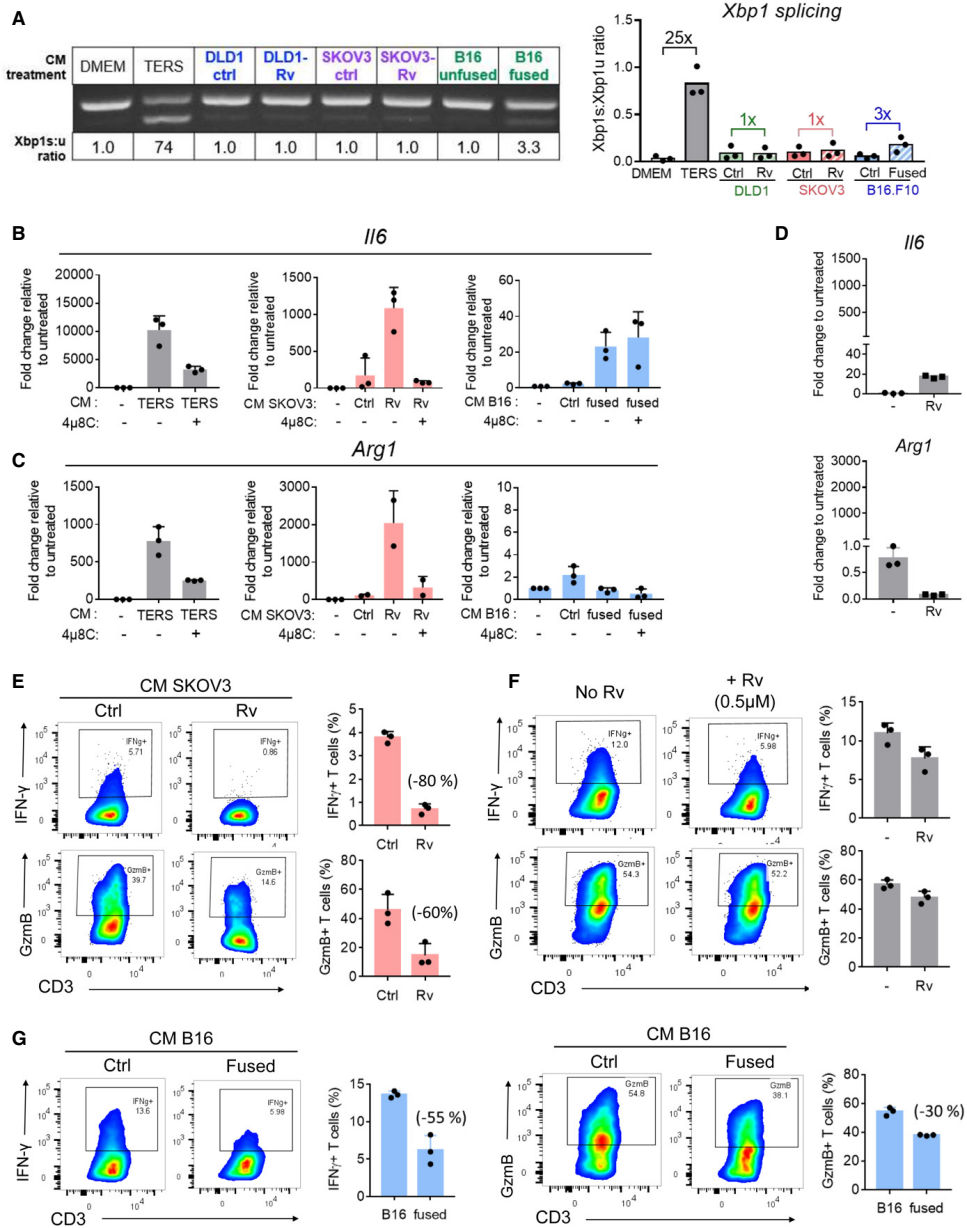
Cell‐nonautonomous effects of aneuploid cancer cells on bone marrow‐derived macrophages and T cells AXbp1 splicing analysis (left panel) and quantification (right panel) of bone marrow‐derived macrophages (BMDM) cultured in conditioned medium (CM) of Rv‐treated DLD1 and SKOV3 cells or respective control medium (culture medium of cancer cells not treated with Rv), and CM of fused B16 melanoma cells or their nonfused parental cells.B, CmRNA expression (RT–PCR) of *Il6* (B) and *Arg1* (C) in BMDM cultured in the CM of Rv‐treated SKOV3 cells (0.5 µM for 3 days) or control cells, and CM of fused B16 cells or their nonfused parental cells. Data points refer to triplicate experiments expressed as means ± SD.DmRNA expression of *Il6* (upper panel) and *Arg1* (lower panel) in BMDM cultured in complete medium spiked with Rv (0.5 µM). Untreated BMDM were used as control. Data representative triplicate samples run in duplicate at the same time point repeated at least twice and expressed as means ± SD.EFlow cytometry analysis of IFN‐γ and granzyme B production by human T cells activated by anti‐CD3/anti‐CD28 Dynabeads in the presence of CM from Rv‐treated SKOV3 cells or control SKOV3 cells (cultured without Rv). Column graphs represent the percentage expression of IFN‐γ (upper panel) and granzyme B (lower panel). Percentages in parenthesis refer to change from control. Data representative triplicate samples at the same time point repeated at least twice. Data are expressed as means ± SD.FFlow cytometry analysis of IFN‐γ and granzyme B production by human T cells activated by anti‐CD3/anti‐CD28 Dynabeads in complete medium spiked with Rv (0.5 µM). Column graphs represent the percentage expression of IFN‐γ (upper panel) and granzyme B (lower panel). Data representative triplicate samples at the same time point repeated at least twice. Data are expressed as means ± SD.GIFN‐γ (left) and granzyme B (right) production by human T cells activated by anti‐CD3/anti‐CD28 Dynabeads cultured in CM from fused B16 melanoma cells or their parental nonfused cells. Percentages in parenthesis refer to change from parental nonfused cells. Data representative triplicate samples at the same time point repeated at least twice. Data are expressed as means ± SD.

Previously, we demonstrated that the conditioned medium (CM) of ER‐stressed cancer cells polarizes macrophages and dendritic cells to a pro‐inflammatory/immune‐suppressive phenotype, impairing antigen‐specific activation of T cells (Mahadevan *et al*, 2011, 2012). Subsequently, we demonstrated that these effects are operational *in vivo* and contribute to tumor development in a IRE1α‐dependent manner (Batista *et al*, 2020). The present TCGA analysis showed an inverse correlation between single SCNA score and CYT across disease stages, suggesting that tumor cells with experimentally induced aneuploidy could also dysregulate immune cells through a cell‐nonautonomous mechanism. To this end, the CM of aneuploid cells collected at the time of maximal *XBP1* splicing was added to cultures of murine bone marrow‐derived macrophages (BMDM) for 24 h. We then isolated their total RNA and analyzed the expression of the canonical pro‐inflammatory cytokine (*Il6*) and the immune‐suppressive enzyme Arginase 1 (*Arg1*) (Rodriguez *et al*, 2005). A schematic representation of the workflow for the experiment is shown in Appendix Fig S11. Definitive *Xbp1* splicing was observed in BMDM treated with the CM of fused B16 cells but only slightly in BMDM treated with the CM Rv‐treated cells suggesting that established aneuploid cells are more efficient at inducing a UPR in BMDM transcellularly (Fig 7A). Next, we looked at *Il6* gene expression, a pro‐inflammatory/tumorigenic cytokine (Grivennikov *et al*, 2009). The CM of Rv‐treated SKOV3 and fused B16 cells yielded high *Il6* induction relative to respective CM controls (6‐fold and 10‐fold, respectively) (Fig 7B). The CM of Rv‐treated SKOV3 also yielded high *Arg1* expression levels compared with control cultures (23‐fold and 3‐fold, respectively) (Fig 7C). Fused B16 cell CM was ineffective at inducing *Arg1*. To exclude a bias due to Rv, BMDM were cultured in DMEM spiked with Rv (0.5 µM) (Fig 7D). Although Rv was found to upregulate *Il6* compared with untreated BMDM, the increase was minimal compared with that induced by the CM from Rv‐treated SKOV3 cells (Fig 7B). Paradoxically, Rv decreased *Arg1* transcription over control (Fig 7C and D) ruling out the possibility that the effect of CM from Rv‐treated SKOV3 was due to Rv carryover.

Previously, we reported that cross‐priming of CD8 T cells by dendritic cells treated with the CM of ER‐stressed tumor cells leads to defective T‐cell activation and clonal expansion (Mahadevan *et al*, 2012). Here, we interrogated the possibility of cell‐nonautonomous effects of aneuploid cells on two key functional parameters (IFN‐γ and granzyme B) of human T cells isolated from normal blood and activated using anti‐CD3 plus anti‐CD28 Dynabeads in undiluted CM. Activation in the presence of CM from Rv‐treated (0.5 µM) SKOV3 cells showed marked reduction of both IFN‐γ and granzyme B (80% and 60%, respectively) relative to control CM (untreated SKOV3 cells) (Fig 7E). The effect could not be attributed to Rv carryover since treatment of T cells with complete medium spiked with Rv (0.5 µM) had only modest effects on both targets (Fig 7F). A similar reduction of IFN‐γ and granzyme B (55% and 30%, respectively) was observed with fused B16 cells compared to their parental nonfused cells (Fig 7G).

Taken together, these data suggest a functional link between aneuploidy, UPR, and a dysregulation of both macrophages and T cells similar to those characteristics of these cells in the tumor microenvironment. In macrophages, which represent the major population infiltrating most solid tumors in humans (Gentles *et al*, 2015) and often display a mixed pro‐inflammatory/immune‐suppressive phenotype (Mahadevan & Zanetti, 2011), we found that the CM of aneuploid cells also imparted IRE1α‐dependent transcription of *Il6* and *Arg1*. This is consistent with previous reports demonstrating the induction of this phenotype in BMDM transcellularly (Mahadevan *et al*, 2011) which appears to be IRE1α regulated (Batista *et al*, 2020). In T cells, we demonstrated that the CM of RV‐treated or fused B16 cells markedly reduced the production of IFN‐γ and granzyme B in T cells activated through the T‐cell receptor mimicking the dysregulation state of tumor‐infiltrating T cells in humans.

## Discussion

This study was set to test the hypothesis that cellular stress resulting from aneuploidy would trigger the UPR and negatively affect key components of local cellular immunity in human cancers, macrophages, and T cells (Zanetti, 2017). Using a single SCNA score inclusive of whole chromosome, arm, and focal SCNA (aneuploidy burden) across 9,375 TCGA samples across 32 tumor types, we confirm a previous report (Davoli *et al*, 2017) and provide clear evidence for an inverse correlation between SCNA and intratumor cytolytic activity (CYT), a proxy of local immune competence, in progressive stages of disease. In the same set of tumor samples, we found that activation of the UPR correlated with both SCNA levels and local immune dysregulation, implicating it as the likely causal link.

At the pan‐cancer level, we showed that UPR gene expression correlates positively with aneuploidy, with genes of the PERK pathway showing strong positive correlation with SCNA across almost every tumor type. An analysis of DLD1 and SKOV3 cells treated with Rv by PCR and Western blotting showed that Rv‐induced aneuploidy triggers a global UPR with upregulation of GRP78, the master regulator of the UPR, and activation of both the IRE1α and PERK branches (Fig 6E and F). We detected clear phosphorylation of eIF2α downstream of PERK but did not detect transcription or translation of ATF6. This may indicate that ATF6 activation is delayed relative to IRE1α and PERK (Durose *et al*, 2006), or simply insufficient, providing for a pro‐survival dynamics since ATF6 is an important regulator of the pro‐apoptotic factor CHOP downstream of ATF4 (Yang *et al*, 2020) and a negative regulator of stemness (Spaan *et al*, 2019). This scenario is consistent with previous work from this laboratory showing that the UPR induced through transcellular transmission results in reduced activation of ATF4 (Rodvold *et al*, 2017) to favor cell survival and self‐renewal.

PERK's homeostatic role in response to stress is the phosphorylation of eIF2α (eIF2α‐P) to inhibit global translation and attenuate the impact of client proteins inside the ER. Therefore, a positive correlation between SCNA and PERK is not surprising. Furthermore, since the UPR is an adaptive response, it follows that tolerance of aneuploidy predisposes to an adaptive UPR, which heightens cellular fitness and dysregulates local immune cells. PERK engagement in tumor promotion can be a response to cell‐autonomous (Bi *et al*, 2005; Hart *et al*, 2012) as well as cell‐nonautonomous (Rodvold *et al*, 2017) stress signals enabling cancer cell survival. We previously showed that cell‐nonautonomous signaling among cancer cells drives, paradoxically, a reduction of ATF4 and CHOP activation downstream of eIF2α (Rodvold *et al*, 2017), hence avoiding apoptosis under condition of acute stress.

Our analysis also shows that PKR and GCN2 are not phosphorylated in Rv‐treated cells. This is not surprising in that these two kinases phosphorylate eIF2α in response to dsRNA and nutrient starvation (Donnelly *et al*, 2013), i.e., exogenous stressors, whereas aneuploidy is an endogenous stressor. Furthermore, the modest activation of ATF4 in Rv‐treated cancer cells is consistent with lack of detectable activation of PKR and GCN2 since ATF4 is an important effector of the ISR (Pakos‐Zebrucka *et al*, 2016). Although the UPR and the ISR share the common hub eIF2α, our data suggest that aneuploidy phosphorylates eIF2α via the UPR as the main homeostatic regulatory mechanism. Arguably, the GCN2‐eIF2α‐ATF4 pathway, which is critical for maintaining metabolic homeostasis in tumor cells (Ye *et al*, 2010), is apparently not a characteristic of aneuploid cells. Therefore, a logical conclusion is that aneuploid cells are effectively avoiding apoptosis through full activation of ATF4 (Han *et al*, 2013; Hiramatsu *et al*, 2020) to their survival advantage.

eIF2α‐P also regulates the translation of molecules relevant to immune dysregulation and tumorigenicity. For instance, eIF2α‐P post‐translationally regulates PD‐L1 expression in MYC transgenic/KRAS mutant murine tumor (Xu *et al*, 2019). Furthermore, eIF2α‐P redirects the translation of 5’‐untranslated regions (5’‐UTRs) (Sendoel *et al*, 2017). The data on co‐expression presented here point to increased negative regulation of the apoptotic program together with an enhancement of metabolic/bioenergetic fitness of the cell. Thus, it is tempting to speculate that through the induction of the UPR, aneuploidy regulates the translational machinery of the cancer cell in a more complex way than just through a gene dose effect. Studies in preneoplastic cells show that eIF2α‐P can direct the translational machinery toward eIF2A‐dependent uORF translation increasing ribosome occupancy of 5’‐UTRs and augmenting protein synthesis (Sendoel *et al*, 2017). The extent to which this phenomenon is exploited by SCNA in cancer cells will need future exploration.

A weak negative correlation between SCNA and IRE1α expression does not preclude an involvement of IRE1α in response to aneuploidy. In fact, we found that the IRE1α‐dependent RIDD activity correlates positively with SCNA and negatively with CYT in several tumor types. RIDD activity degrades selected target mRNAs halting their translation (Hollien & Weissman, 2006), a function somehow complementary to eIF2α‐P in that both RIDD and eIF2α‐P regulate proteostasis to diminish the workload of client proteins in the endoplasmic reticulum during times of stress (Maurel *et al*, 2014). Of interest RIDD also degrades miRNAs (Upton *et al*, 2012; Wang *et al*, 2018) including miR‐34a which negatively regulates PD‐L1 expression (Wang *et al*, 2015), induces cellular senescence by modulating telomerase activity (Xu *et al*, 2015), and inhibits adrenergic transdifferentiation of tumor‐associated sensory nerves in a p53‐dependent manner (Amit *et al*, 2020). Our PCR analysis of BLOC1S1, a conserved RIDD target (Bright *et al*, 2015), in Rv‐treated SKOV3 and DLD1 cells shows mRNA degradation in SKOV3 only at the 72‐h time point, a finding reflecting cell type variability in RIDD activation and/or low IRE1α activation in aneuploid cells. In fact, it has been shown that BLOC1S1 is specifically cleaved by IRE1α only under conditions of IRE1α hyperactivation (Bright *et al*, 2015).

The apparent discrepancy between pan‐cancer analysis and PCR data in Rv‐treated tumor cells can be explained considering that the pan‐cancer analysis most likely reflects RIDD activation in infiltrating myeloid cells rather than the tumor cells *per se*, with macrophages representing the major population infiltrating most solid tumors in humans (Gentles *et al*, 2015). In agreement with this interpretation, we observed reduced expression of RIDD target genes in tumor‐infiltrating macrophages versus tumor cells (Fig EV5). Furthermore, we recently reported that RIDD activity is readily induced in wild type but not in *Ern1*‐conditional knock‐out bone marrow‐derived macrophages treated with the CM of ER‐stressed tumor cells, and IRE1α but not PERK predicts *CD274 (PD‐L1)*, a gene under regulation miR‐34a itself a RIDD target (Upton *et al*, 2012), in macrophages isolated from human endometrial or breast cancers (Batista *et al*, 2020). Collectively, these considerations suggest that aneuploidy directly effects the UPR in tumor cells, mainly targeting the PERK pathway and transcellularly tumor‐infiltrating macrophages via IRE1α and RIDD in tumor. This dichotomy is obviously relevant to novel therapies targeting the UPR in cancer patients.

In macrophages and dendritic cells, an unintended consequence of transcellular UPR signaling is the acquisition of a pro‐inflammatory/immune‐suppressive phenotype that has been shown in both tumor‐bearing mice and cancer patients (Chittezhath *et al*, 2014; Sousa *et al*, 2015). Here, we show that aneuploidy is a sole trigger of the UPR in cancer cells (Fig 6) and modulates the phenotype of macrophages transcellularly leading to the transcriptional activation of *Il6,* the gene coding for a pro‐inflammatory/tumorigenic cytokine, and *Arg1*, the gene coding for a T‐cell‐suppressive enzyme. A small molecule inhibitor of IRE1α RNAse activity markedly diminished *Il6* and *Arg1* transcription, suggesting that transcellular regulation of myeloid immune cells by aneuploid cancer cells is also IRE1α‐XBP1 dependent (Cubillos‐Ruiz *et al*, 2015; Batista *et al*, 2020).

Our data show that IFN‐γ and granzyme B are both down‐regulated in T cells activated in the presence of CM from aneuploid cells. This effect is new and consistent with our pan‐cancer analysis showing a significant inverse correlation between the CYT score and the SCNA score in all cancer stages (Fig 2 and Table 1). It is also consistent with reports showing that IFN‐γ and granzyme B are down‐regulated in exhausted intratumoral T cells compared with non‐exhausted T cells or with peripheral blood T cells (Chauvin *et al*, 2015; Iga *et al*, 2019; Wu *et al*, 2014). Although the mechanism underlying this dysregulation of T cells is presently unknown, the phenomenon is relevant since it provides a new and additional explanation for the hypofunctionality of tumor‐infiltrating T cells (Thommen & Schumacher, 2018). Congruently, previous reports showed that CD8 T cells cross‐primed by dendritic cells pretreated with the CM of ER‐stressed tumor cells are severely impaired in antigen‐driven clonal expansion by Mahadevan *et al* (2012) and T cells treated with ascitic fluid of ovarian cancers have reduced IFN‐γ production (Song *et al*, 2018).

Collectively, the data add a new layer of complexity to our understanding of the origin of immune dysregulation in the tumor microenvironment. If in fact signals emanating from aneuploid cells impart a pro‐tumorigenic phenotype to macrophages, and by extension to dendritic cells, focus should be placed on blocking community effects rather than cognate cell–cell interactions. For instance, establishing the role of IRE1α in macrophages and dendritic cells isolated from human cancers should be prioritized as this could lead to a new therapeutic angle to subvert local immune dysregulation.

Cell‐nonautonomous signaling through the UPR has been documented in *C*. *elegans,* increasing longevity and establishing neuroimmune axis communication (Taylor & Dillin, 2013; van Oosten‐Hawle *et al*, 2013; O'Brien *et al*, 2018; Frakes *et al*, 2020). A UPR‐based transcellular communication has also been documented between cancer cells and bone marrow‐derived myeloid cells (macrophages and dendritic cells) (Mahadevan *et al*, 2011, 2012; Cubillos‐Ruiz *et al*, 2015; Rodvold *et al*, 2017). Both in *C*. *elegans* (Taylor & Dillin, 2013; van Oosten‐Hawle *et al*, 2013; O'Brien *et al*, 2018; Frakes *et al*, 2020) and in myeloid cells (Cubillos‐Ruiz *et al*, 2015; Batista *et al*, 2020), the cell‐nonautonomous effects appear to depend on the IRE1α ‐XBP1 axis in receiver cells. Although the nature of the transmitting factor(s) has remained elusive in most reports, a UPR‐driven transcellular communication is of clear relevance to the immunobiology of tumor‐infiltrating myeloid cells (macrophages and dendritic cells) and T cells.

An unanswered question raised by the present study is *when* aneuploidy exerts its effects on the UPR relative to tumor history. It is known that aneuploidy increases during tumor evolution (Newburger *et al*, 2013; Ben‐David & Amon, 2020) and correlates with poor prognosis (Owainati *et al*, 1987; Stopsack *et al*, 2019). Here, we show that aneuploidy increases as the tumor progresses from stage I through stage IV (Fig 2A). As shown, SCNA^high^ tumors differ drastically in gene co‐expression patterns relative to SCNA^low^ tumors, suggesting that SCNA also drives loss of connectivity among genes (Fig 4C). Compared to other genomic alterations timed to early cancer evolution such as driver mutations (Vogelstein *et al*, 2013) and chromothripsis (Consortium ITP‐CAoWG, 2020), the impact of aneuploidy on the UPR may be stochastic as suggested by a loss of connectivity among UPR genes in the SCNA^high^ group across tumor types. Paradoxically, ovarian cancer, a tumor with the highest aneuploidy burden, shows only a weak correlation with the UPR. However, all ovarian tumors have high levels of aneuploidy, making it difficult to measure variation.

Aneuploidy is an early event that determines genomic instability (Duesberg *et al*, 1998). Our data suggest that a single SCNA score (aneuploidy burden) encompassing whole‐chromosome, arm, and focal aneuploidy is sufficient to establish a positive correlation with the UPR and an inverse correlation with intratumor T‐cell immunity. Current predictors of the response to immune checkpoint blockade include tumor mutational burden and DNA hypomethylation, which itself correlates with aneuploidy (Jung *et al*, 2019; Tripathi *et al*, 2019). Therefore, standardized methods to assess aneuploidy burden on an individual basis (Douville *et al*, 2018) could help better stratify patients likely to respond to immune checkpoint blockade therapies. Remarkably, aneuploidy‐driven UPR propagates its effects transcellularly suggesting that an unappreciated consequence of aneuploidy in cancer cells is to polarize macrophages to a pro‐tumorigenic phenotype, hence remodeling the tumor immune microenvironment to evade immune surveillance. In conclusion, we suggest that aneuploidy exacts a two‐pronged toll on tumor evolution: one by providing fitness advantage to cancer cells (Pavelka *et al*, 2010) and the other in initiating/amplifying local immune cell dysregulation promoting immune evasion.

## Materials and Methods

### Reagents and Tools table

**Table:** 

Reagent/Resource	Reference or source	Identifier or Cat. No.
**Chemicals & Cytokines**		
Reversine	Abcam	ab120921
Thapsigargin	Tocris	1138
Calyculin‐A	Cell signaling	9902S
M‐CSF	R&D Systems	416‐ML
4µ8C inhibitor	Sigma	SML0949
**Cell lines**		
DLD1	ATCC	CCL‐221
SKOV3	ATCC	HTB‐77
Polyploid B16	(Searles *et al*, 2018)	
**Antibodies**		
PercP‐Cy5.5 anti‐human CD3	BioLegend	300328
PE anti‐human IFN‐γ	BioLegend	502509
Alexa Fluor 647 anti‐human granzyme B	BioLegend	515406
Fixable Viability Dye eFluor 780	Thermo Fisher Scientific	65‐0865‐14
ATF4 (C‐20) antibody	Santa Cruz	sc‐200
ATF6 (D4Z8V) antibody	Cell Signaling	65880
IRE1a (14C10) antibody	Cell Signaling	3294
GAPDH (A‐14)	Santa Cruz	sc‐20358
GCN2 (phospho T899) antibody	Abcam	ab75836
GRP78/BIP antibody	BD Bioscience	610978
PERK (C33E10) antibody	Cell Signaling	3192
phospho‐eiF2a (D9G8) antibody	Cell Signaling	3398
PKR (phosphor T446) antibody	Abcam	ab32036
Rabbit IgG‐HRP antibody	Cell Signaling	7074
Mouse‐IgGκ BP‐HRP antibody	Santa Cruz	sc‐516102
Donkey anti‐goat IgG‐HRP antibody	Santa Cruz	sc‐2020
**Oligonucleotides**		
XBP1 forward 5′‐AGGGGAATGAAGTGAGGCCA‐3′	Integrated DNA Technologies	
XBP1 reverse 5′‐TGTGGTCAAAACGAATTAGT‐3’	Integrated DNA Technologies	
ERN1 forward proprietary	Thermo Fisher Scientific	Cat no. 4331182 assay Hs00980097_m1
ERN1 reverse proprietary	Thermo Fisher Scientific	Cat no. 4331182 assay Hs00980097_m1
ATF4 forward proprietary	Thermo Fisher Scientific	Cat no. 4331182 assay Hs00909569_g1
ATF4 reverse proprietary	Thermo Fisher Scientific	Cat no. 4331182 assay Hs00909569_g1
ATF6 forward proprietary	Thermo Fisher Scientific	Cat no. 4331182 assay Hs00232586_m1
ATF6 reverse proprietary	Thermo Fisher Scientific	Cat no. 4331182 assay Hs00232586_m1
DDIT3 forward proprietary	Thermo Fisher Scientific	Cat no. 4331182 assay Hs01090850_m1
DDIT3 reverse proprietary	Thermo Fisher Scientific	Cat no. 4331182 assay Hs01090850_m1
*IL‐6* forward proprietary	Thermo Fisher Scientific	Cat no. 4331182 assay Mm00446190_m1
*IL‐6* reverse proprietary	Thermo Fisher Scientific	Cat no. 4331182 assay Mm00446190_m1
*Arg1 reverse* *proprietary*	Thermo Fisher Scientific	Cat no. 4331182 assay Mm00475988_m1
*Arg1 reverse* *proprietary*	Thermo Fisher Scientific	Cat no. 4331182 assay Mm00475988_m1

### Methods and Protocols

#### Data

The TCGA files were downloaded from the gdc portal on 12/27/2017, using gdc‐client v1.3.0. TCGA RNA‐seq alignment files were reprocessed using sailfish software version 0.7.4 and the GRCh38 reference genome with default parameters, and including all read sequence duplicates. Associated metadata were downloaded using TCGA REST API interface https://api.gdc.cancer.gov/files/. The MSI data were downloaded from (Kautto *et al*, 2017) supplementary data. We used a threshold of 0.4 as the cutoff for distinguishing MSI‐H and MSS as suggested in this paper. Annotated somatic mutation calls from TCGA Pan‐Cancer were downloaded from the GDC on 12/17/2016. TCGA Segmented SNP6 array data were downloaded from Broad Firehose (release stddata_2016_01_28, file extension: segmented_scna_hg19).

#### Somatic copy number alteration quantification

We considered three categories of SCNA as described previously (Davoli *et al*, 2017): whole chromosome, chromosome arm, and focal copy number alterations. SCNAs were detected by comparing Affymetrix SNP data between tumor and paired normal samples. Based on the SNP intensity at the corresponding genomic position, we define a region as a contiguous set of SNPs with a shared log_2_ fold change in intensity. A region was designated an event if the log_2_ fold change exceeded certain thresholds. A log_2_ fold change greater than 0.1 or less than −0.1 was defined as a single event, and a log_2_ fold change greater than 1 or less than −1 as two events (equation 1) (Beroukhim *et al*, 2010). Thus,

(1) $$Events_{i}=\left\{\begin{array}{cc} 1, & if\;\left(0.1<\log_{2}FC\right)\;or\;\left(\log_{2}FC<‐0.1\right)\\ 2, & if\;\left(1<\log_{2}FC\right)\;or\;\left(\log_{2}FC<‐1\right)\\ 0, & \mathrm{else} \end{array}\right.,$$

where *i* indexes regions of contiguous SNPs with the same intensity. Most regions are small, thus to score whole‐chromosome arms using equation (1), we used the fractional length weighted sum of log_2_ fold changes across the regions within a chromosome arm *j* (equation 2).

(2) $$ArmIntensity_{j}=\sum_{i\in j}\log_{2}FC_{i}\cdot \frac{length\left(i\right)}{length\left(\mathrm{\backslash hskip-.1ptj}\right)}$$

An event was designated whole chromosome if both chromosome arms met the Equation 1 criteria such that both arms were affected in the same direction, chromosome arm if one arm met the Equation 1 criteria or the arms were affected in different directions, and focal otherwise. Chromosomal and arm events were only counted once, in the largest category that applied. As focal events can happen subsequent to loss or gain of a chromosome or arm, we did not constrain counting of focal events based on the larger categories.

Events of each category were then summed for each sample. Whole chromosome and focal events were summed across 23 chromosomes, and chromosome arm‐level events were summed across 46 possible chromosome arms. As the resulting scores have very different ranges, (0–46 for chromosomal events, 0–92 for arm‐level events, and much larger values for focal events), we scaled each of these values before combining them into a single SCNA score (Equation 3) using sklearn.preprocessing. MinMaxScaler, with a feature range from 0 ˜ 1.

(3) $$\begin{array}{cc} SCNA= & scaled\left(\mathrm{FocalSCNA}\right)+scaled\left(\mathrm{ArmSCNA}\right)\\ & +scaled\left(\mathrm{ChromosomalSCNA}\right) \end{array}$$

Since the FocalSCNA, ArmSCNA, and ChromosomalSCNA were all transformed to the same scale before aggregating, we interpret this SCNA score as a general reflection of genome abnormality, considering the 3 categories as contributing equally.

#### Cytolytic activity

The cytolytic activity (CYT) score was calculated as the geometric mean of log_2_ TPM expression values of granzyme A (*GZMA*) and perforin (*PRF1*) as described in Rooney *et al* (2015).

#### SCNA correlation with non‐silent mutation

We partitioned the TCGA samples into MSS (*n* = 8,536) and MSI‐H (*n* = 373) using the MSI data downloaded from Kautto *et al* (2017). We then removed silent mutations and computed the total number of mutations per sample. The relationship between the aggregated SCNA score and total number of non‐silent somatic mutations was evaluated by Spearman correlation coefficient in MSS and MSI‐H samples separately.

#### *Tp53* mutations and P53 activity analysis

TCGA samples were partitioned into *TP53* wild‐type and *TP53*‐mutated groups. Twenty‐five tumor types included at least one sample with *TP53* mutation (Fig S2A). The Wilcoxon rank‐sum test was applied to test the aggregated SCNA score differences between *TP53* wild‐type and *TP53*‐mutated groups within each tumor type (Fig S2A). P53 activity was calculated as the sum of z‐scored log_2_ TPM expression values of 10 P53 downstream repressed genes, including *CCNB1, PLK1, EED, CDK1, EZH2, CCNB2, E2F3, MYBL2, FOXM1, and E2F2* (Cancer Genome Atlas Research Network. Electronic address and Cancer Genome Atlas Research, 2017). Since P53 repression of these genes indicates P53 activity, the inverse of this value was used as the score representing P53 activity. This was done using sklearn.preprocessing.MinMaxScaler. The relationship between the P53 activity score and SCNAs score was assessed by Spearman correlation coefficient.

#### OLS models fitting SCNA and CYT with tumor stages

An ordinary least square (OLS) linear model (equation 4) was used to relate SCNA and CYT scores to tumor stage, including tumor type as a covariate to predict the independent variable using 6495 samples with stage information from 25 tumor types (ACC, BLCA, BRCA, CESC, CHOL, COAD, ESCA, HNSC, KICH, KIRC, KIRP, LIHC, LUAD, LUSC, MESO, OV, PAAD, READ, SKCM, STAD, TGCT, THCA, UCEC, UCS, and UVM).

(4) y∼TumorType+TumorStage

In equation (4), *y* represents SCNA score or CYT score. Tumor stages and tumor types were encoded as categorical variables.

#### Effects of SCNA score on UPR gene expression

TCGA samples were divided into three groups, SCNA^high^, SCNA^low^ and neither using the 30^th^ and 70^th^ percentiles of SCNA level within each tumor type. UPR gene expression levels were compared between SCNA^low^ and SCNA^high^ groups using the Wilcoxon rank‐sum test to determine whether there was a significant shift in expression between groups. Multiple hypothesis testing correction was performed using the Benjamini–Hochberg method with alpha = 0.05.

#### Differential co‐expression analysis of UPR pathway genes

Differential co‐expression analysis was applied to test for pairwise co‐expression changes between SCNA^high^ and SCNA^low^ samples (as defined above), using the method from Tesson *et al* (2010). First, the adjacency matrix for each phenotype was constructed by the following formula (equation 5), where $c_{\mathrm{ij}}^{\mathrm{phenotype}}$ represents the Spearman correlation coefficient between gene *i* and *j* in a specified phenotype.

(5) $$c_{\mathrm{ij}}^{\mathrm{phenotype}}=cor\left(gene_{i},gene_{j}\right)$$

Then, the adjacency matrix difference is computed as follows (equation 6), with the β parameter set to 4.

(6) $$D_{\mathrm{ij}}={\left(\sqrt{\frac{1}{2}\left|\text{sign}\left(c_{\mathrm{ij}}^{\text{low}}\right)*{\left(c_{\mathrm{ij}}^{\text{low}}\right)}^{2}‐\text{sign}\left(c_{\mathrm{ij}}^{\text{high}}\right)*{\left(c_{\mathrm{ij}}^{\text{high}}\right)}^{2}\right|}\right)}^{\beta }$$

We then permuted SCNA group membership 1,000 times within each tumor type to generate a null distribution for evaluating the significance of the pairwise correlation. This analysis included all 58 UPR genes, resulting in 3,364 gene pairs. We identified gene pairs that showed less correlation than expected across more than nine tumor types as recurrently perturbed, and pairs that showed more correlation than expected across all tumor types as preserved. Conserved gene pairs were further assessed by Spearman correlation pan‐cancer, and only pairs that showed significant Spearman correlation (FDR < 0.05, multiple correction after Benjamini–Hochberg) were retained. The median co‐expression change was calculated for each tumor type by summing the spearman correlation coefficient differences between SCNA^low^ and SCNA^high^ groups ($c_{\mathrm{ij}}^{\text{low}}‐c_{\mathrm{ij}}^{\text{high}}$) for each gene pair and taking the median (Fig 4A). The number of gene pairs significant after permutation testing is shown in the side bar of Fig 4A. The differences in correlation coefficient for selected gene pairs between SCNA^low^ and SCNA^high^ are shown in Fig S6.

#### GO Enrichment Analysis for selected gene pairs

We performed GO biological process analysis separately for 41 genes with recurrently perturbed co‐expression patterns, 11 genes with augmented co‐expression, and 35 genes with conserved co‐expression patterns identified from differential co‐expression analysis (above). GO Enrichment Analysis was performed using the online server http://geneontology.org/, using the “biological process complete” annotation data set with Homo sapiens reference list. The test result is calculated using Fisher’s exact, with FDR cutoff < 0.05.

#### UPR branch pathway score quantification

Gene sets representing PERK (Reactome id R‐HSA‐381042.1), XBP1s (Reactome id R‐HSA‐381038.2), and ATF6 (Reactome id R‐HSA‐381183.2) branch pathway downstream activity were extracted from the Reactome pathway database (Fabregat *et al*, 2018). The RIDD pathway downstream gene set was obtained from Maurel *et al* (2014). We implemented the pathway score quantification method from Schubert *et al,* 2018; however instead of applying non‐regularized linear regression as in their work, we used Lasso regression to avoid overfitting and reduce redundant features. We built Lasso regression models using 10‐fold cross‐validation to select the lambda parameter. In order to fit models that would represent the extent of induction of UPR branch pathways, we modeled the dependent variable using paired tissue‐matched samples in TCGA such that *y* = 0 for tissue‐matched normal samples and *y* = 1 for tumor samples. For each pathway, log_2_ TPM expression values of genes downstream of the branch pathway served as the independent variables. Models were fit in each tumor type separately. Matrix multiplication between the UPR branch gene expression matrix and the model coefficient matrix was applied to quantify pathway scores of individual pathways (XBP1s, PERK, ATF6, and RIDD) for each sample. Because the coefficient matrix represents the vector of corresponding genes in the plain of expression space, the pathway score is a meaningful representation of the distance from the origin (Schubert *et al*, 2018). Using this method, we obtained pathway scores for 7998 samples, across 23 tumor types that had normal tissue RNA‐seq data available. Pathway scores were compared to SCNA and CYT scores by Spearman correlation.

#### OLS model fitting UPR pathways, tumor types, and SCNA to predict CYT

We fit an OLS model with XBP1s, PERK, ATF6, RIDD branch pathway scores, tumor type, tumor purity, and SCNA scores as independent variables *x* to predict the dependent variable CYT score, *y* (equation 7). Tumor purity was approximated by immunohistochemistry (IHC) measures obtained from Tirosh *et al* (2016). These data were available for 16 tumor types (*n* = 7,802; 16 tumor types: BLCA, BRCA, CESC, COAD, HNSC, KICH, KIRC, KIRP, LIHC, LUAD, LUSC, PRAD, READ, SKCM, THCA, and UCEC).

(7) CYT∼XBP1s+PERK+ATF6+RIDD+SCNA+IHC+TumorType

A second model was fit excluding IHC (*n* = 8,488; 23 tumor types: BLCA, BRCA, CESC, CHOL, COAD, ESCA, HNSC, KICH, KIRC, KIRP, LIHC, LUAD, LUSC, PAAD, PCPG, PRAD, READ, SARC, SKCM, STAD, THCA, THYM, and UCEC) using the formula.

(8) CYT∼XBP1s+PERK+ATF6+RIDD+SCNA+TumorType

#### Single‐cell analysis or RIDD pathway genes

We retrieved the single‐cell data from Tirosh *et al* (2016) GSE72056. This dataset included measurements for 33 RIDD target genes. We performed hierarchical clustering of single cells using the 33 RIDD target genes with the Ward variance minimization algorithm using python package Scipy. We focused the analysis on tumor cells and macrophages as macrophages are the most abundant immune cell‐infiltrating tumors (Cassetta *et al*, 2019) and are involved in mediating cell‐nonautonomous effects that dysregulate the tumor microenvironment (Mahadevan *et al*, 2011) including RIDD activity (Batista *et al*, 2020). We excluded *ITGB2* and *TAPBP*, since *ITGB2* and *TAPBP* from further analysis, as they did not behave in the same way as other RIDD target genes (Batista *et al*, 2020). Mean expression of RIDD target genes was further compared using the Wilcoxon rank‐sum test.

#### Software version, packages, and code availability

Computational analysis was performed using Python version 2.7.15. The OLS regression models used statsmodels.formula.api, version 0.9.0. Wilcoxon rank‐sum tests, Spearman correlation analysis, hierarchical clustering, and z‐score calculations used scipy version 1.1.0. The LASSO regression model with cross‐validation was applied using sklearn.linear_model.LassoCV, version 0.20.3. All rescaling was done using sklearn.preprocessing.MinMaxScaler, version 0.20.3. Plots were generated using matplotlib version 2.2.3 and seaborn version 0.9.0. Data representation used pandas version 0.24.2. Code and data to reproduce the analysis are available at https://github.com/cartercompbio/SCNA_score_analysis.

#### Digital Karyotyping

Digital Karyotyping analysis was performed using Illumina Infinium Core‐24 Beadarrays, which allow interrogation of >500,000 SNPs at single‐nucleotide resolution. These arrays produce data from intensity signals corresponding to the presence of allele A and allele B at a given SNP. Using GenomeStudio (Illumina), we calculated the mean log‐R ratio, a measure of copy number as a ratio of observed to expected intensities, and the B‐allele frequency, the proportion of allele calls at each genotype with respect to allele B (1.0 for B/B, 0.5 for A/B, and 0.0 for A/A). We created plots using these metrics to visually inspect each chromosome for abnormalities. For each kit, we used 200 ng of DNA, which was processed according to manufacturer instructions. Following hybridization, BeadChips were scanned using the Illumina iScan System.

#### Cell lines and culture conditions

The quasi‐diploid cell lines DLD1 (colorectal adenocarcinoma) (Knutsen *et al*, 2010) and SKOV3 (ovarian carcinoma) (Buick *et al*, 1985b) were grown in complete DMEM (Corning) supplemented with 10% FBS (HyClone). Polyploid B16 × MEF fused clonal lines (Searles *et al*, 2018) and were kindly provided by Dr. Jack Bui (Department of Pathology, UCSD), and grown in complete RPMI media (Corning). All cells were maintained at 37°C with 5% CO_2_ and were mycoplasma free as determined a PCR assay (Southern Biotech).

#### BMDM generation in culture

Bone marrow‐derived macrophages (BMDM) were generated by isolating the femur and tibia of C57Bl/6 mice (8–12 weeks old) and flushing out the bone marrow using cold, unsupplemented RPMI growth medium (Corning) using a 27‐gauge needle and syringe. Red cells were lysed using ACK Lysis buffer (Bio Whittaker). Macrophage differentiation from bone marrow cells was obtained by culture in standard growth medium supplemented with m‐CSF (R&D Systems) at 30 ng/ml for 7 days. Bone marrow donor mice were housed in the UCSD vivarium. Femurs were removed per UCSD IACUC‐approved protocol in compliance with animal welfare standards.

#### RNA isolation and cDNA synthesis

RNA was harvested from cells using Nucleospin II Kit (Macherey‐Nagel). Concentration and purity of RNA were quantified the NanoDrop (ND‐1000) spectrophotometer (Thermo Scientific) and analyzed with NanoDrop Software v3.8.0. RNA was normalized between conditions and cDNA generated using the High Capacity cDNA Synthesis Kit (Life Technologies).

#### XBP1 splicing assay

cDNA was subjected to the Xbp1 splicing assay as a surrogate outcome measure for ER stress. Primers were developed flanking the region of Xbp1 excised following UPR activation: Forward—5′‐AGGGGAATGAAGTGAGGCCA‐3′, Reverse—5′‐TGTGGTCAAAACGAATTAGT‐3’. PCR was run on a Thermocycler (Thermo Scientific) using under the following conditions: 30 s at 94°C, 40 s at 55°C, 30 s at 72°C for 35 cycles and 5 min at 72°C. PCR products were run overnight on a 3% agarose gel at 30V for separation. Unspliced Xbp1 appears as the “upper band” at 358 bp, while the spliced isoform appears as the “lower band” at 332 bp. Data analysis and quantification of Xbp1 splicing was performed using ImageJ software.

#### RT–qPCR

cDNA was subjected to RT–qPCR using an ABI 7300 Real‐Time PCR system and TaqMan reagents for 50 cycles under universal cycling conditions. Cycling conditions followed manufacturer’s specifications (KAPA Biosystems). Target gene expression was normalized to β‐actin and relative expression determined by using the ‐ΔΔCt relative quantification method. Primers for RT–qPCR were purchased from Life Technologies: Arg1 (Mm00475988_m1) and IL‐6 (Mm99999064_m1).

## Author contributions

MZ contributed to original concept; HC and MZ contributed to project supervision; HC, SX, SCS, and MZ involved in project planning and experimental design; TCW involved in statistical advising; MD, GA, SCS, and PS contributed to in vitro experiments; SX, MD, GA, SCS, KJ, AC, and MZ contributed to data acquisition, processing, and analysis; SX, MD, GA, SCS, KJ, AC, and MZ analyzed the data; HC and MZ wrote the manuscript.

## Conflict of interest

The authors declare that they have no conflict of interest.

## Supporting information



Appendix

Expanded View Figures PDF

Dataset EV1

Table EV1

Table EV2

Table EV3

## Data Availability

Bioinformatic data have been deposited in https://github.com/cartercompbio/SCNA_score_analysis.
