# Supplementary material for: The unfolded protein response links tumor aneuploidy to local immune dysregulation
Source: EMBO Rep. 2021 Oct 26;22(12):e52509. doi: 10.15252/embr.202152509 (PMC8647024; doi:10.15252/embr.202152509)
Supplement: Supplementary file 1 — Appendix [file EMBR-22-e52509-s006.pdf]

Appendix

Supplementary Figures

The unfolded protein response links tumor aneuploidy to local immune dysregulation

Su Xian, Magalie Dosset, Gonzalo Almanza, Stephen Searles, Paras Sahani, T. Cameron Waller, Kristen Jepsen, Hannah Carter and Maurizio Zanetti

Table of Contents

	Page
**Appendix Fig. S1**	2
**Appendix Fig. S2**	4
**Appendix Fig. S3**	5
**Appendix Fig. S4**	7
**Appendix Fig. S5**	8
**Appendix Fig. S6**	9
**Appendix Fig. S7**	11
**Appendix Fig. S8**	12
**Appendix Fig. S9**	14
**Appendix Fig. S10**	15

Appendix Fig. S1

A

	Chr	Arm	Focal	SCNA
Chr	1.000	0.548	0.612	0.860
Arm	0.548	1.000	0.627	0.866
Focal	0.612	0.627	1.000	0.735
SCNA	0.860	0.866	0.735	1.000

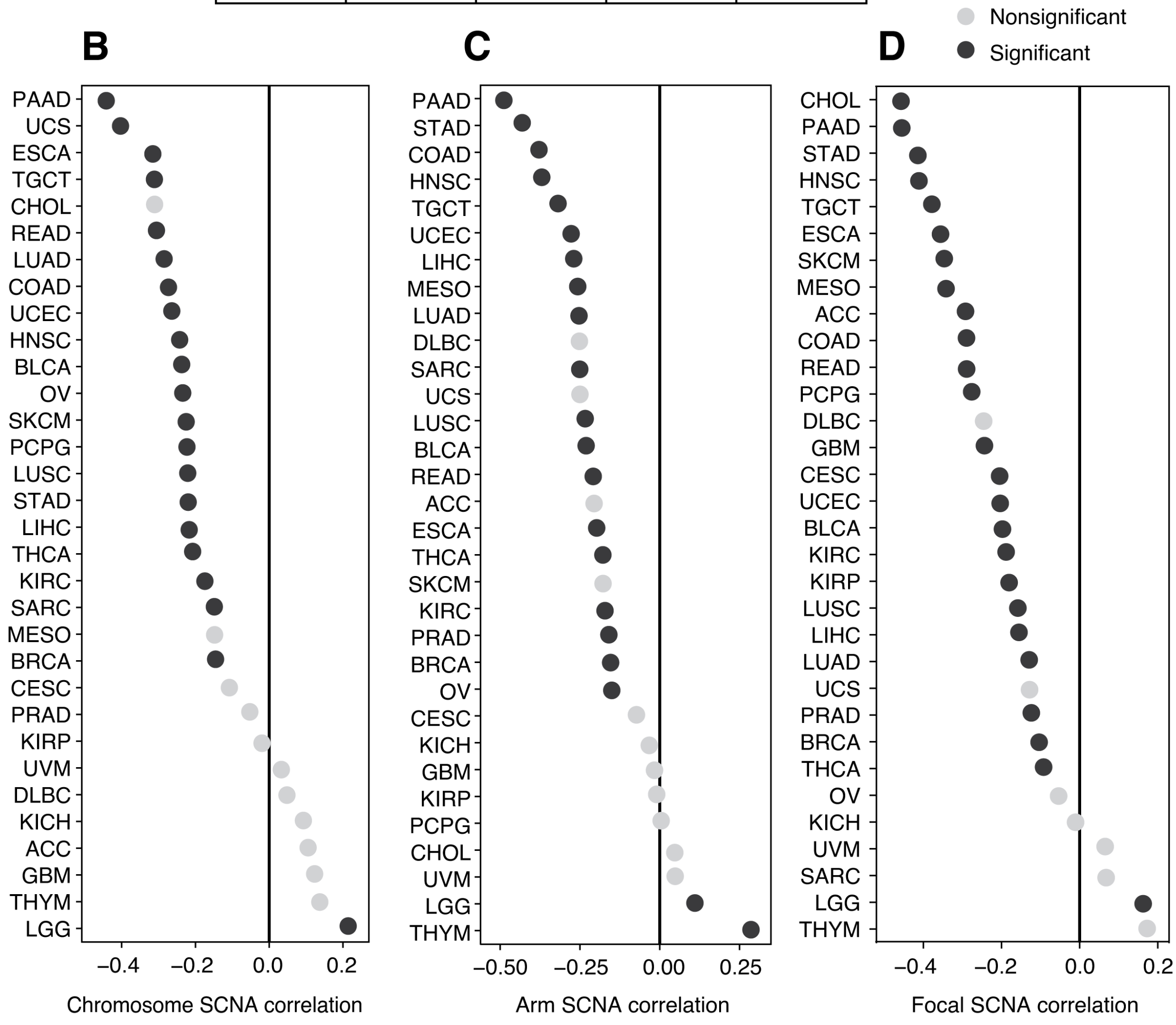

Appendix Figure S1. Three categories of SCNA show inverse correlation with CYT score across tumor types

(A) Pairwise Spearman correlation coefficients show that the 3 categories of SCNA and the combined single SCNA score are positively correlated pancancer.

(B) Chromosome copy-number alteration count correlation with CYT score across 32 tumor types.

(C) Chromosome arm copy-number alteration count correlation with CYT score across 32 tumor types.

(D) Focal copy-number alteration count correlation with CYT score across 32 tumor types.

Appendix Fig. S2

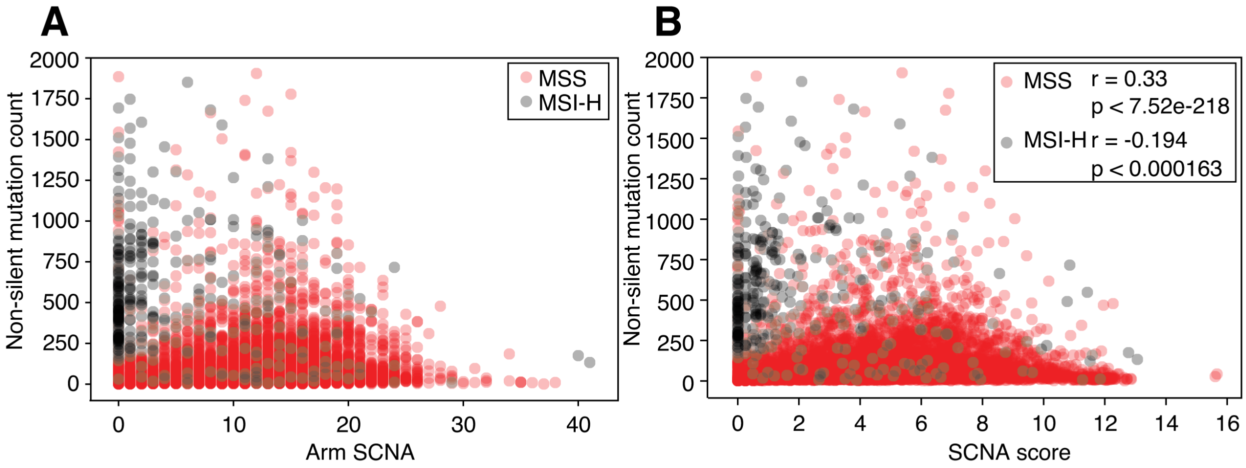

Appendix Figure S2. Pan-cancer analysis of somatic copy number alteration (SCNA) correlation with non-silent mutation burden

(A) Scatter plot of arm level somatic copy-number alteration count (x axis) versus non-silent mutation count (y axis). Red dots represent microsatellite stable (MSS) samples (N = 8536) and black dots represent microsatellite instability high (MSI-H) samples (N = 373).

(B) Scatter plot of tumor SCNA score (x axis) versus non-silent mutation count (y axis). Black dots represent MSI-H samples (N = 373) and red dots represent MSS samples (N = 8536). Spearman correlation for MSS samples is 0.33 ($p < 7.52e-218$). Spearman correlation for MSI-H samples is -0.194 ($p < 0.000163$).

Appendix Fig. S3

A

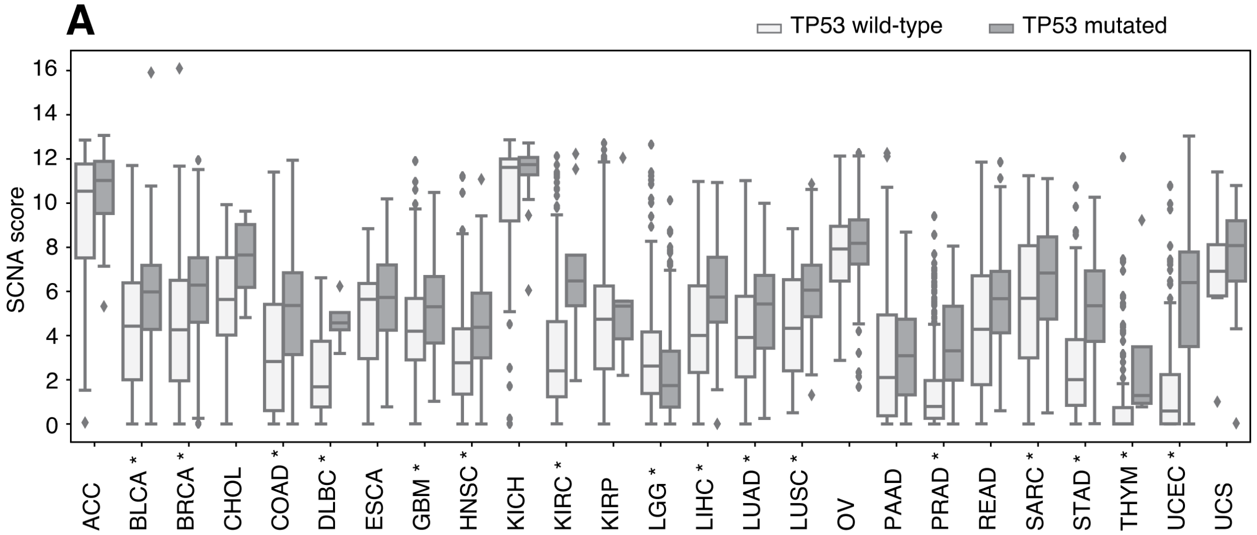

B

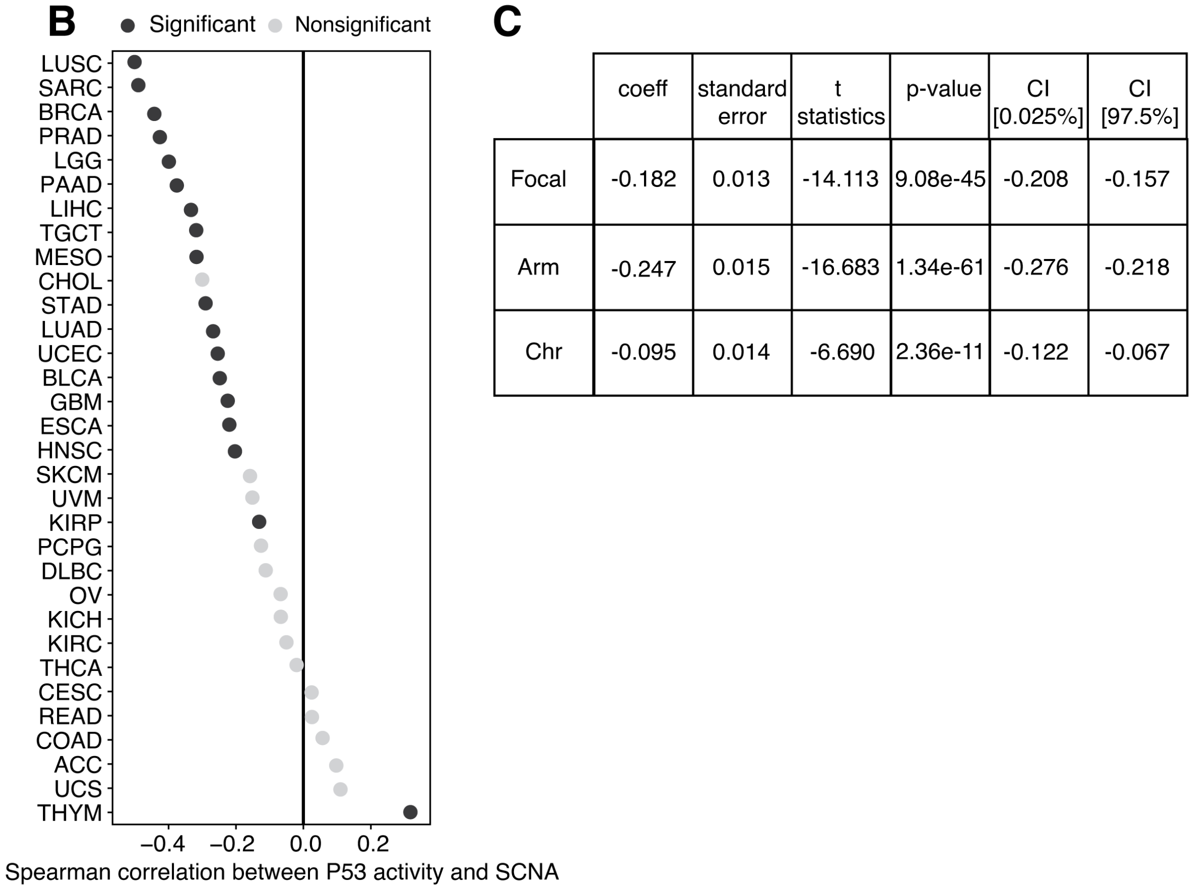

C

	coeff	standard error	t statistics	p-value	CI [0.025%]	CI [97.5%]
Focal	-0.182	0.013	-14.113	9.08e-45	-0.208	-0.157
Arm	-0.247	0.015	-16.683	1.34e-61	-0.276	-0.218
Chr	-0.095	0.014	-6.690	2.36e-11	-0.122	-0.067

Appendix Figure S3. p53 activity correlates with SCNA score.

(A) Boxplots showing SCNA score distribution differences between TP53 mutated samples and TP53 wild-type samples across 25 tumor types with TP53 mutations.

(B) Spearman correlation between p53 activity and SCNA score across 32 tumor types. Black dots represent statistically significant values (FDR < 0.05) and gray dots represents non-significant values (FDR > 0.05).

(C) Coefficients for focal, arm and chromosome level SCNA events in am OLS regression model predicting TP53 activity score across multiple tumor types, with tumor type as a covariate.

Appendix Fig. S4

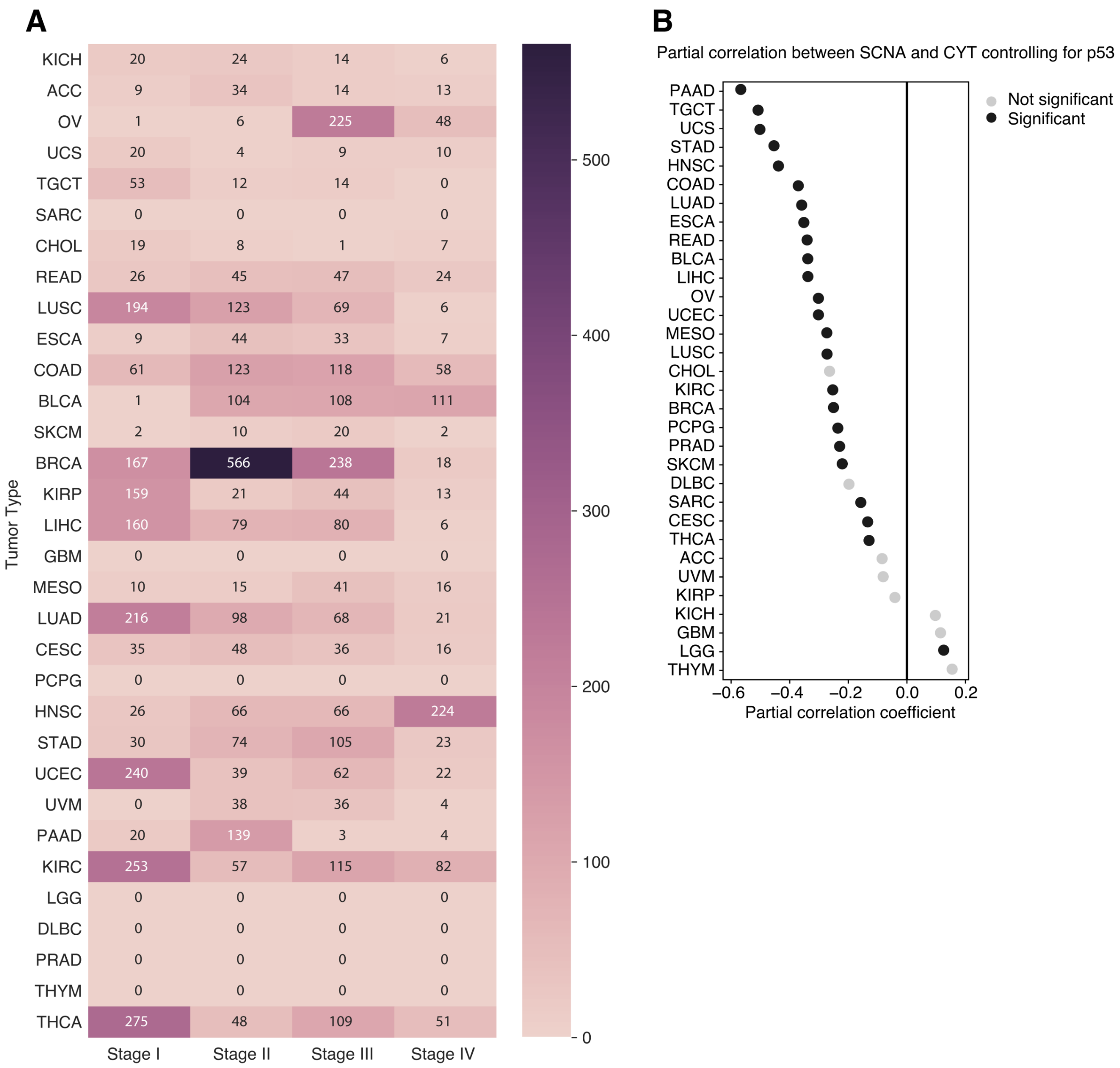

Appendix Figure S4. (A) Heatmap showing the sample size imbalances across 4 tumor stages in 32 tumor types. (B) Partial correlation between SCNA and CYT across 32 tumor types, conditioning on p53 pathway scores. Black dots represent statistically significant values (FDR < 0.05) and gray dots represents non-significant values (FDR > 0.05).

Appendix Fig. S5

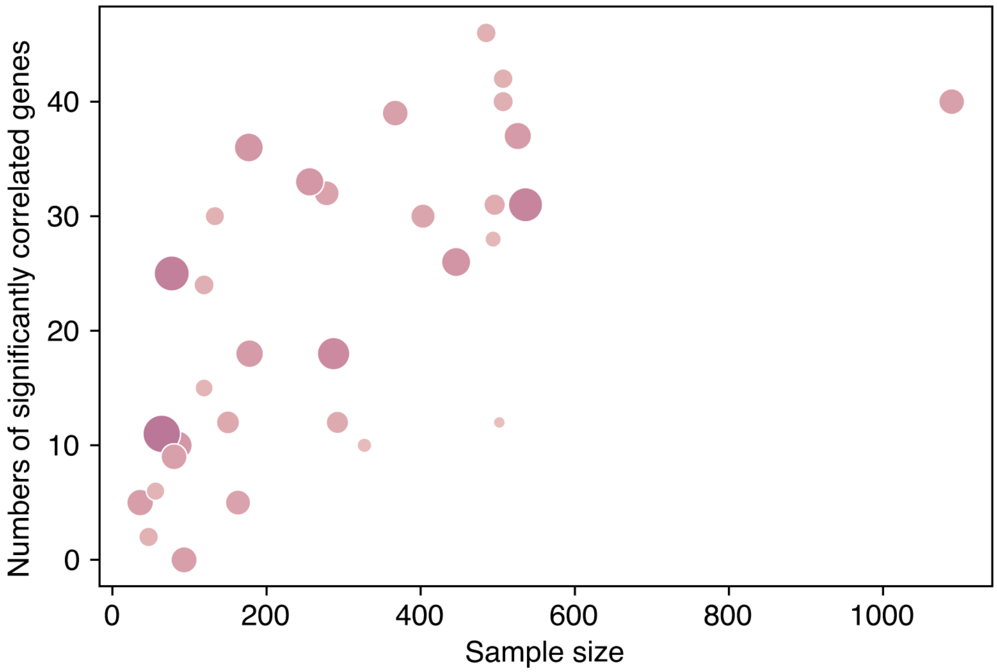

Appendix Figure S5. Sample size limits the number of significant correlations between genes and SCNA scores across 32 tumors

A scatter plot showing the number of samples (x axis) versus the number of UPR genes that significantly correlated with SCNA score after multiple hypothesis testing (y axis). Each dot represents a tumor type in TCGA. The size and color intensity of the dots depicts the variance of the SCNA score across a tumor type.

Appendix Fig. S6

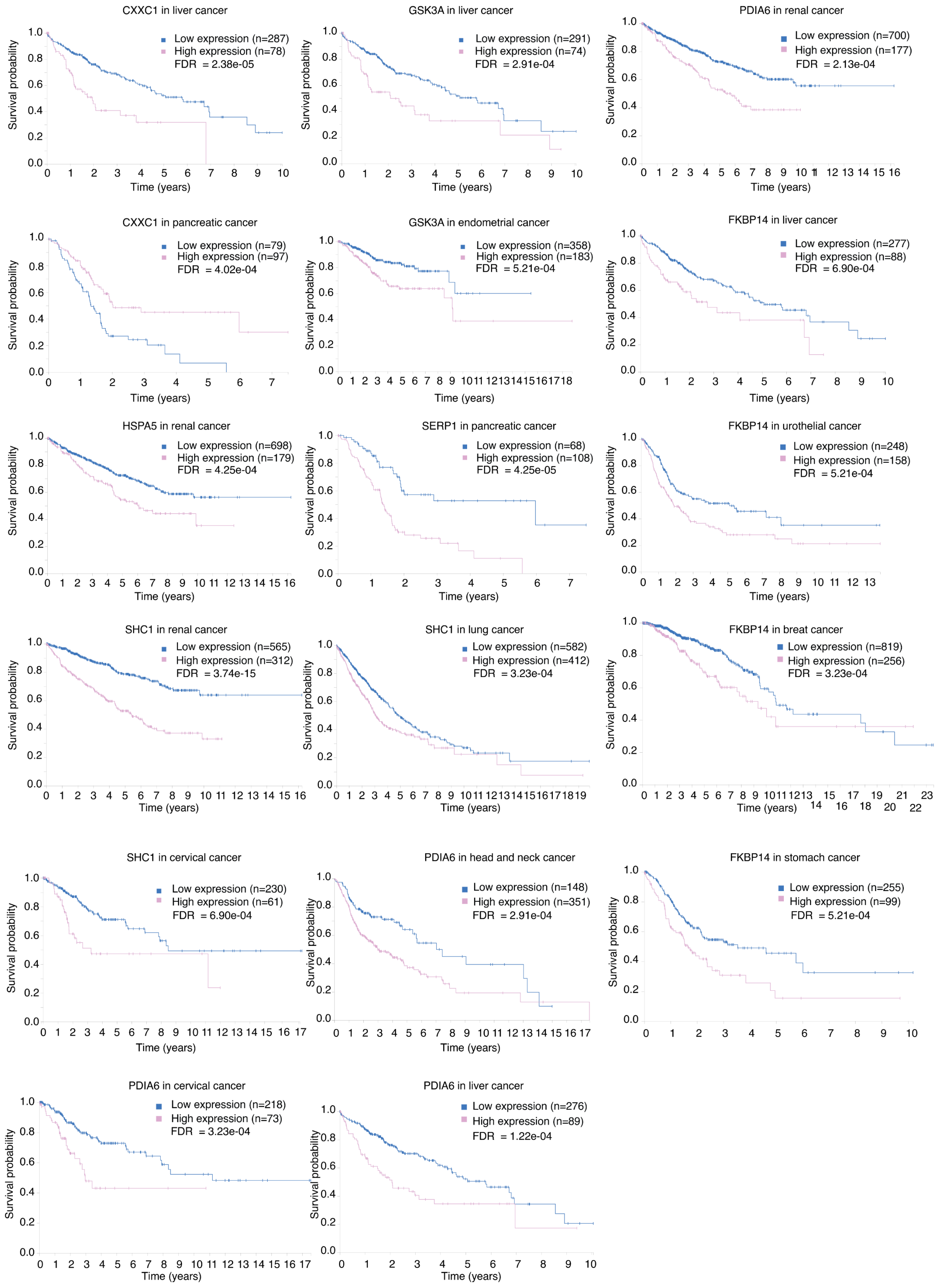

Appendix Figure S6. Poor prognosis linked to genes with loss of coordination under SCNA^{high} conditions. Genes with reduced coordination in expression with three or more partners (*CXXC1, GSK3A, HSPA5, SERP1, SHC1, PDIA6* and *FKBP14)* were input to the HUMAN PROTEIN ATLAS (version 19.3) at http://www.proteinatlas.org, DOI: 10.1126/science.aan2507. This site performs a Kaplan-Meier analysis in 17 tumor types from TCGA, stratifying samples into high or low expression level based on predetermined FPKM cutoffs (55). Plots are returned only if the log-rank test was significant. P values and multiple testing adjusted p values are shown with each plot. In general, higher expression of UPR genes was associated with poorer outcome.

Appendix Fig S7

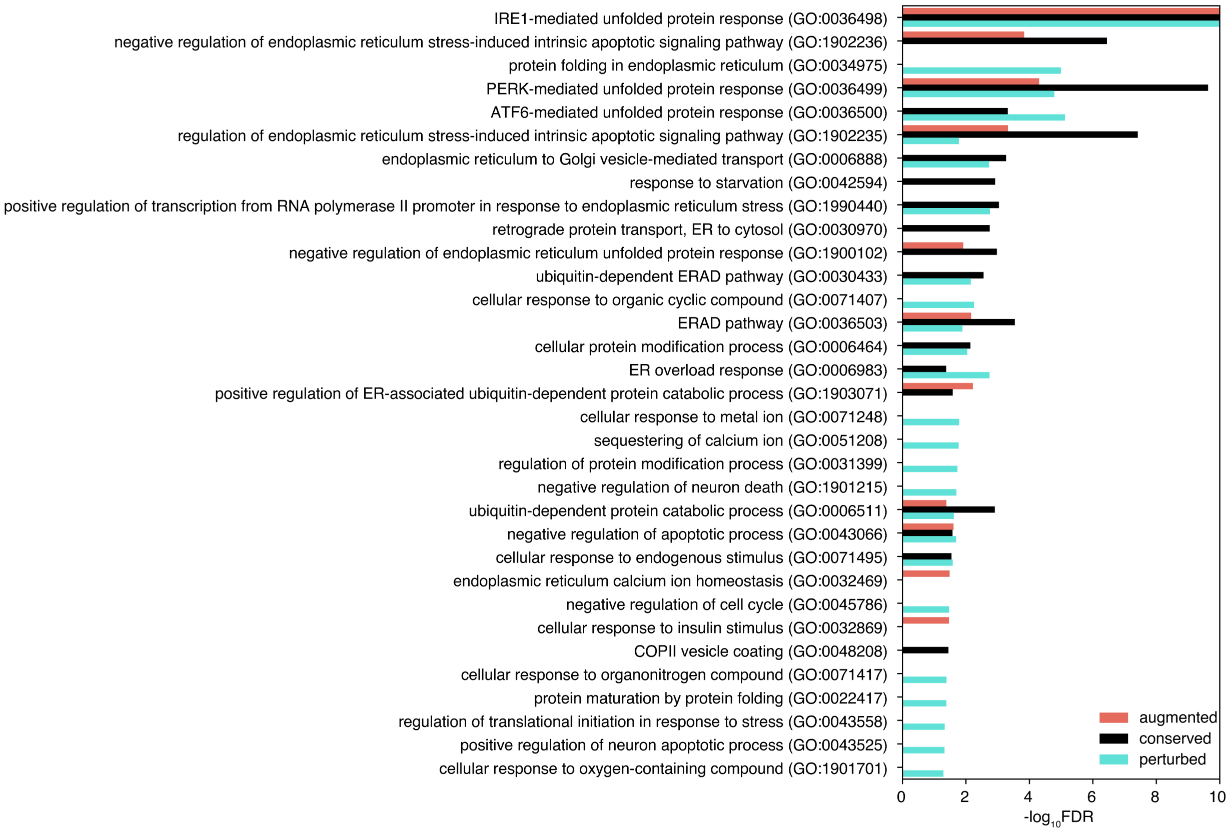

Appendix Figure S7. Gene Ontology Analysis for perturbed, preserved and augmented gene pairs, showing only parental Gene Ontology terms. Negative log10 adjusted p-values of GO terms enriched for preserved, augmented or perturbed genes ordered by median across categories, focusing on parental terms from the GO hierarchy tree. The complete list is available in Table EV3.

Appendix Fig. S8

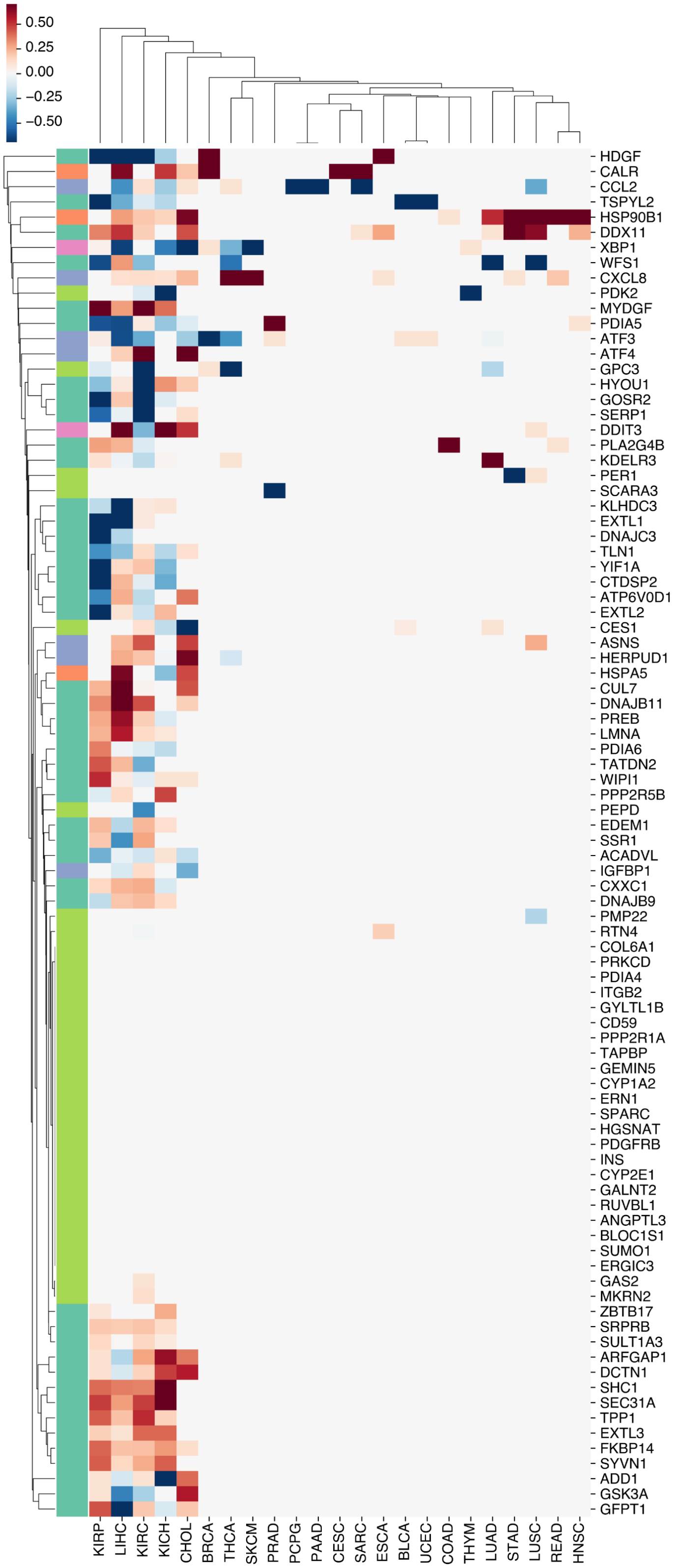

Appendix Figure S8 Genes selected to represent UPR pathway activity by Lasso regression for 23 tumor types. A heatmap showing genes selected by Lasso regression models for three UPR pathways and 23 tumor types during pathway score quantification. Each column represents a tumor type and each row represents a gene. The left color bar indicates gene membership in UPR branch pathways: IRE1 – green; PERK – blue; ATF6 – orange; RIDD – light green; multiple pathways – purple. Each cell in the heatmap is colored according to the magnitude of the coefficient of the gene in the lasso regression model within each tumor type, with red depicting positive coefficients and blue depicting negative coefficients. To achieve a better visualization, coefficients were scaled within each tumor type to fall in the -1 to 1 range and thus represent the relative strength of gene contribution for different tumor types.

Appendix Fig. S9.

Chromosome 11, DLD1

Unstimulated

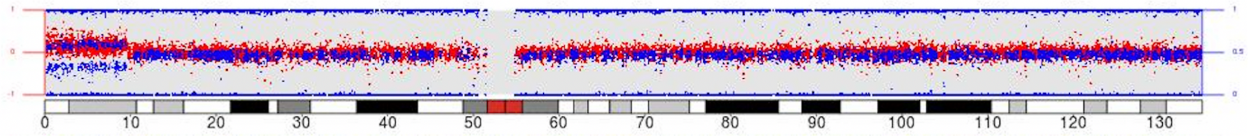

+Rv (14 days)

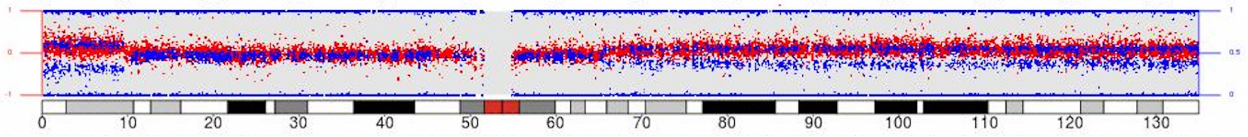

Chromosome 20, DLD1

Unstimulated

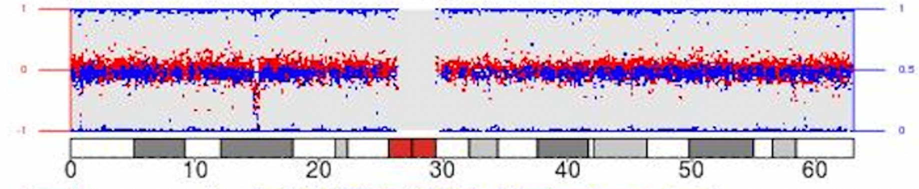

+Rv (14 days)

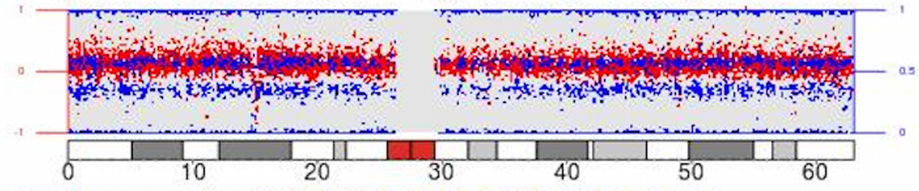

Appendix Figure S9. Digital Karyotyping for Detecting Chromosome Alterations. Mean log R ratio (LRR, in red) and B-allele frequency (BAF, in blue). A. AAB/ABB trisomy specific BAF signal (the two blue horizontal bands) at 11p is present both before and after stimulation, while only stimulated cells show AAB/ABB trisomy specific BAF signal for 11q. On chromosome 20, a small deletion is observed in both untreated and treated cells at 20p, while AAB/ABB trisomy for the entirety of chromosome 20 was observed only in treated cells.

Appendix Fig. S10.

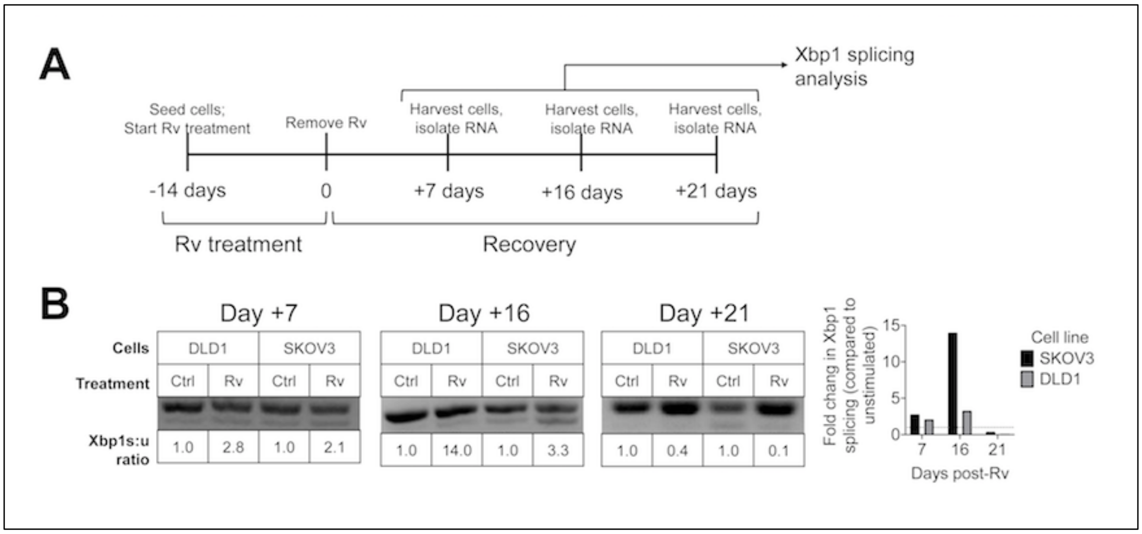

Appendix Figure S10.

(A) Schematic representation of workflow of long-term effects of Rv treatment *in vitro*.

(B) Analysis (left panel) of XBP1 splicing in DLD1 and SKOV3 cells treated with Rv for 14 days and then allowed to recover for additional 7, 16, and 21 days. Bars (right panel) refer to quantification of XBP1 splicing of the same experiment expressed as ratio of spliced: unspliced XBP1 as determined using ImageJ software. Data of one experiment representative of two independent experiments.
