# Supplementary material for: The unfolded protein response links tumor aneuploidy to local immune dysregulation
Source: EMBO Rep. 2021 Oct 26;22(12):e52509. doi: 10.15252/embr.202152509 (PMC8647024; doi:10.15252/embr.202152509)
Supplement: Supplementary file 2 — Expanded View Figures PDF [file EMBR-22-e52509-s002.pdf]

Expanded View Figures

Figure EV1. Three categories of SCNA event show similar trends in correlation with CYT score UPR gene expression.

A Coefficients and p-values of an ordinary least square (OLS) linear model using 3 categories of SCNA to predict CYT score, with tumor type as a covariate.
B Heatmaps showing correlation between the three categories of SCNA event (rows) and UPR parental genes for 32 tumor types (columns). Color intensity indicates magnitude of Spearman correlation, with red indicating positive and blue indicating negative correlation, respectively.

© 2021 The Authors

A

	coeff	p-value
chr	-0.145	2.76e-30
arm	-0.198	1.70e-50
focal	-0.070	1.65e-09

B

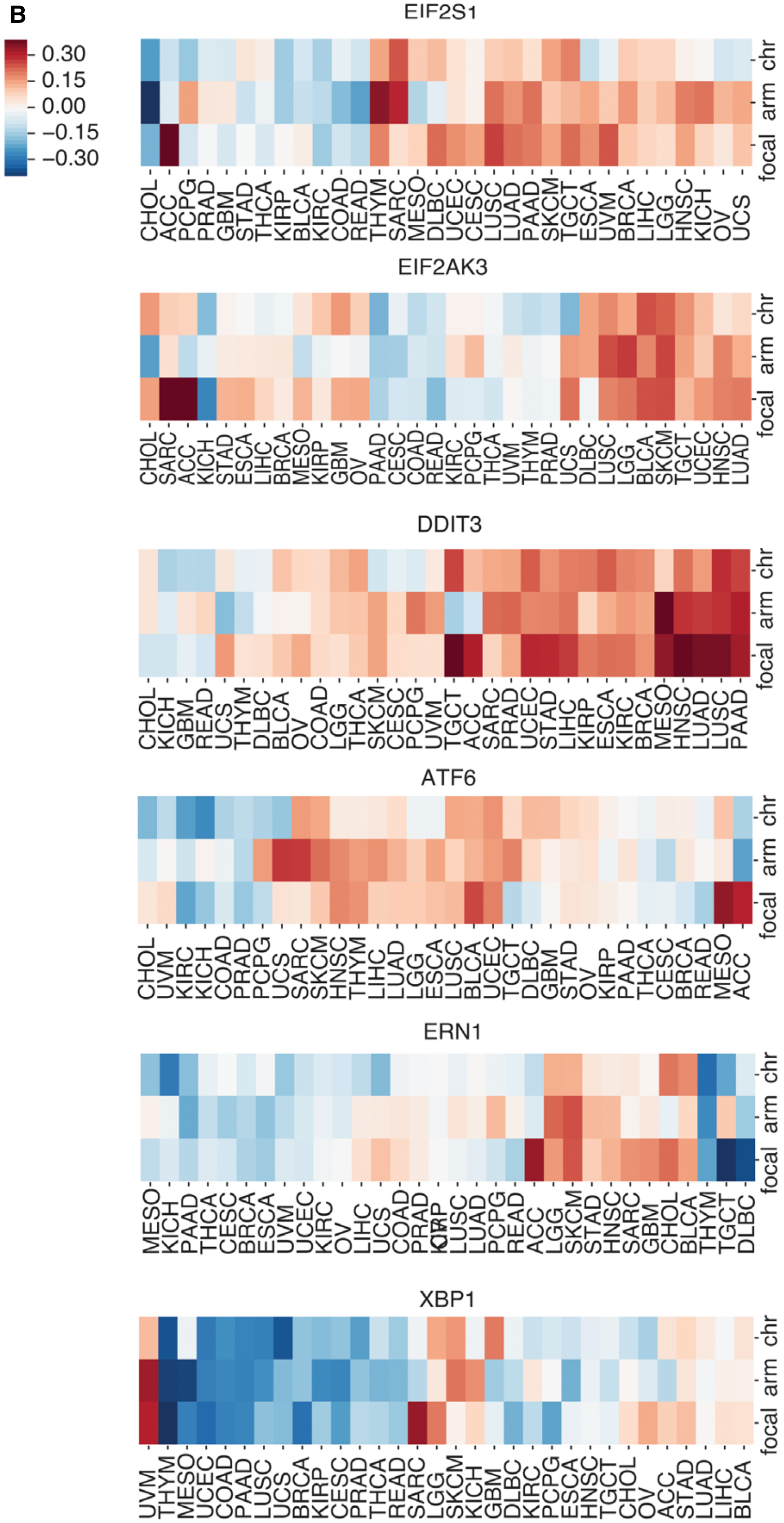

Figure EV1.

 © 2021 The Authors

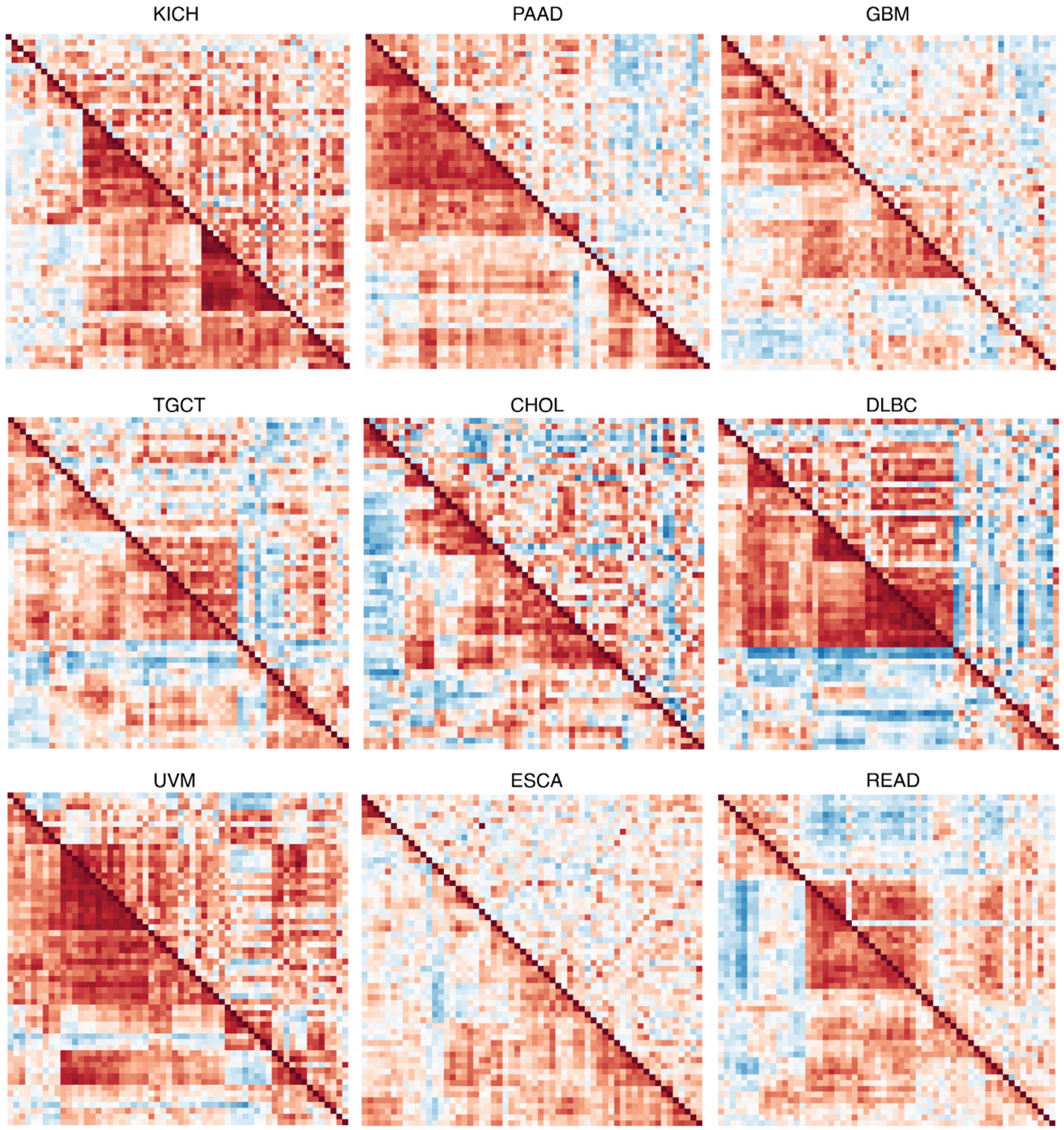

Figure EV2. Heatmaps showing UPR gene co-expression patterns for high and low SCNA groups for nine tumor types.

Bottom left triangles represent the co-expression of UPR genes in the $SCNA^{low}$ group, and top right triangles represent the co-expression of UPR genes in the $SCNA^{high}$ group.

© 2021 The Authors

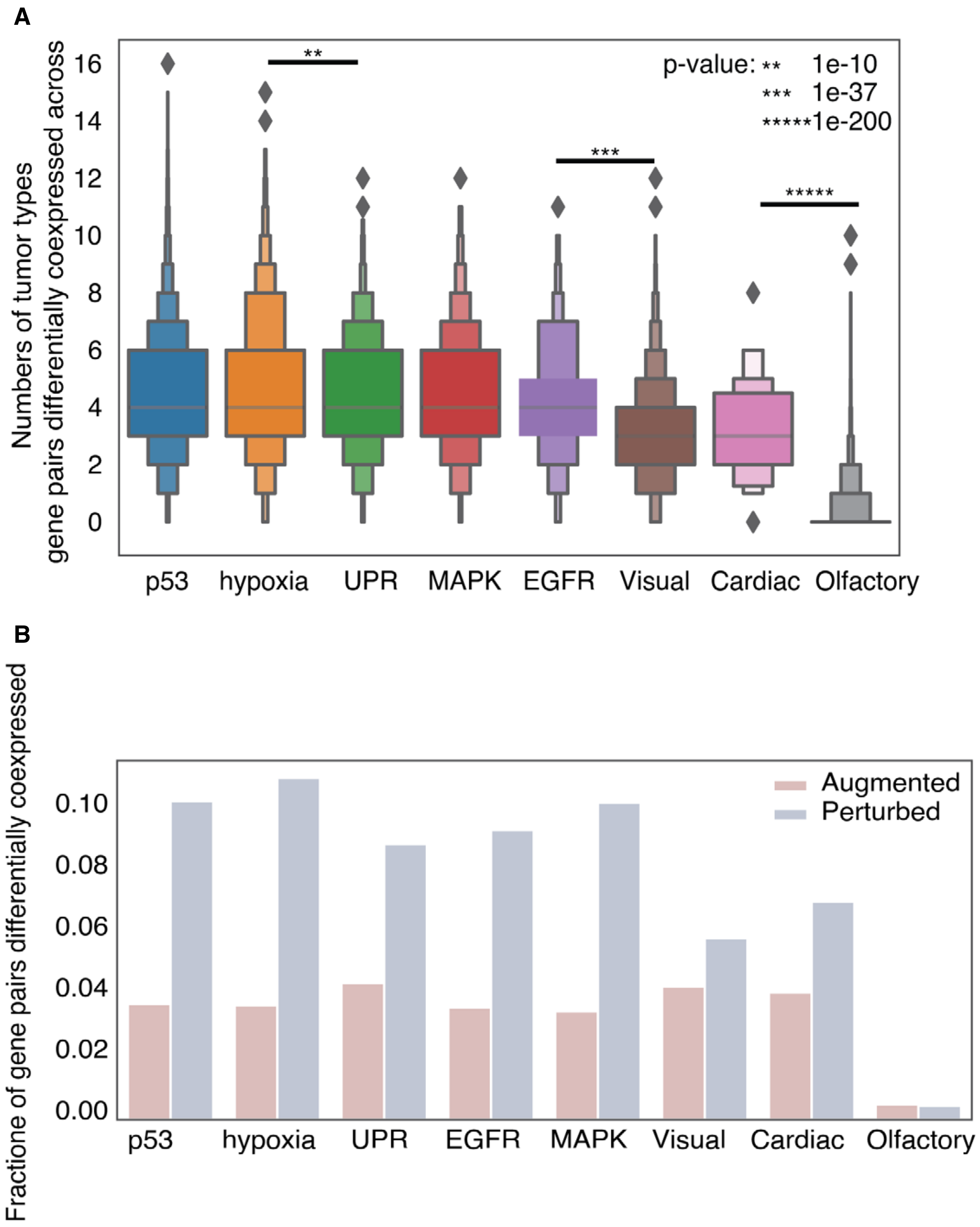

Figure EV3. Comparison of differential co-expression of gene pairs under high and low SCNA conditions across the UPR and seven control pathways.

A Gene pairs are more consistently perturbed across tumor types for the UPR pathway and cancer pathways ($n = 3$) than for control pathways ($n = 3$). The x-axis represents the numbers of tumor types that where a given gene pair was significantly differentially co-expressed. The y-axis represents the specific pathway each gene pairs belongs to. Statistical significance was calculated using the student's t-test.

B UPR and cancer-associated pathways show more perturbed gene pairs than non-cancer control pathways.

 © 2021 The Authors

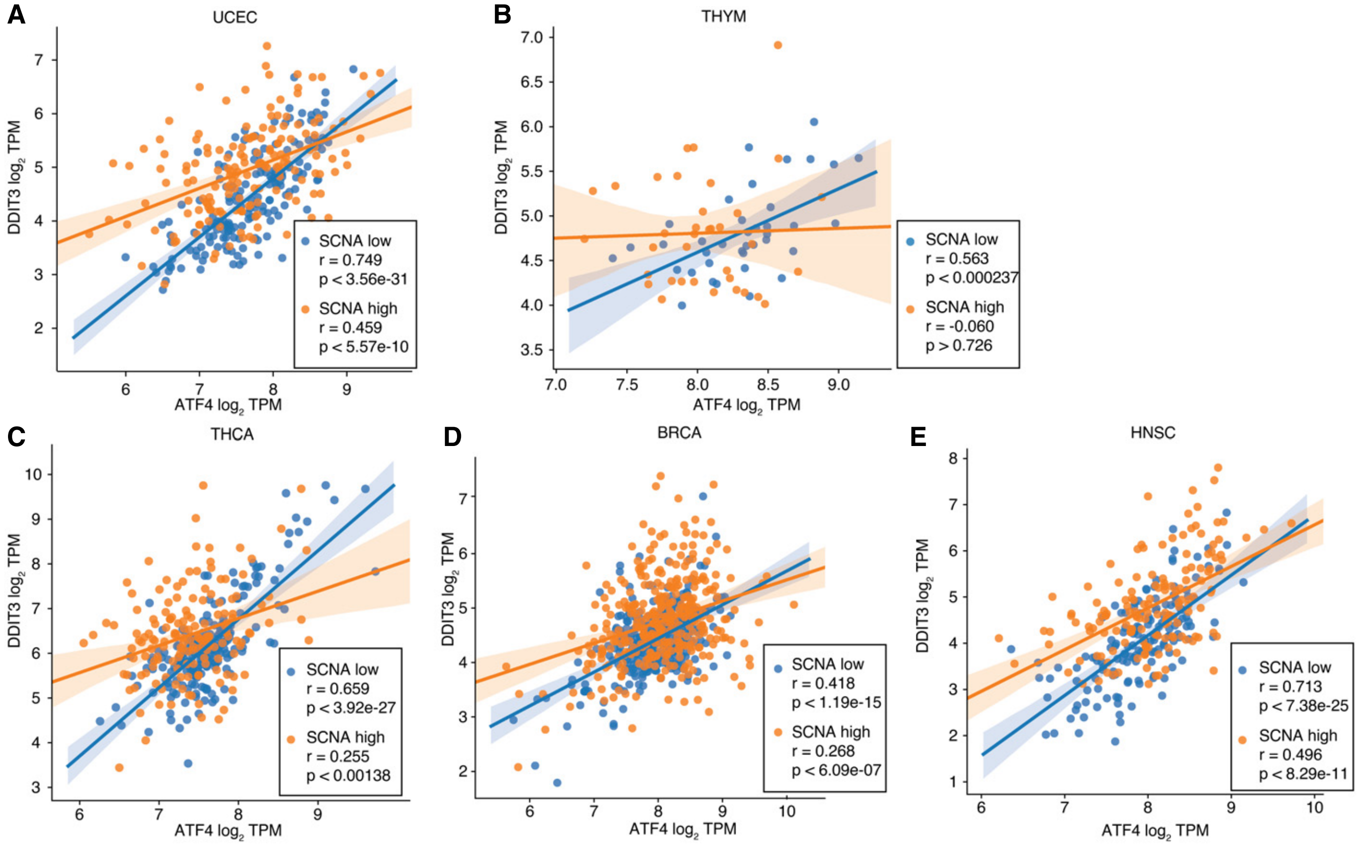

Figure EV4. ATF4 and DDIT3 gene loss of coordination in four tumor types.

A–E Scatter plots showing the log_2 TPM expression level of ATF4 (*x*-axis) and DDIT3 (*y*-axis) in $SCNA^{low}$ (blue) and $SCNA^{high}$ (orange) conditions for tumors from four different tumor types: (A) UCEC—endometrial cancer; (B) THYM—thymoma; (C) THCA—thyroid cancer; (D) BRCA—breast adenocarcinoma; (E) HNSC—head and neck squamous cell carcinoma. Regression lines indicate the strength of correlation between the expression of the two genes. Spearman correlation coefficients and associated p-values are provided.

Figure EV5. Analysis of RIDD target gene expression in single-cell data showed reduced expression in tumor-associated macrophages compared with tumor cells.

A Heatmap showing the unsupervised clustering of 1,257 tumor cells and 119 macrophages (rows) according to expression in $log_2(TPM/10 + 1)$ of 33 RIDD target genes (columns). The left sidebar indicates cell type: red—macrophages; pink—tumor cells.

B Boxplots showing the distribution of mean RIDD target gene expression in macrophages (blue) and tumor cells (orange), $P = 2.73e\text{-}46$, Wilcoxon rank-sum test. ITGB2 and TAPBP were excluded as their behavior is counter to regulation by RIDD.

C Spearman correlation coefficients linking PERK pathway score and RIDD activity score for 12 tumor types for which both pathway scores could be calculated. Black color indicates correlations that were statistically significant after multiple hypothesis testing correction using the Benjamini–Hochberg procedure (FDR < 0.05). Gray dots represent non-significant cases.

© 2021 The Authors

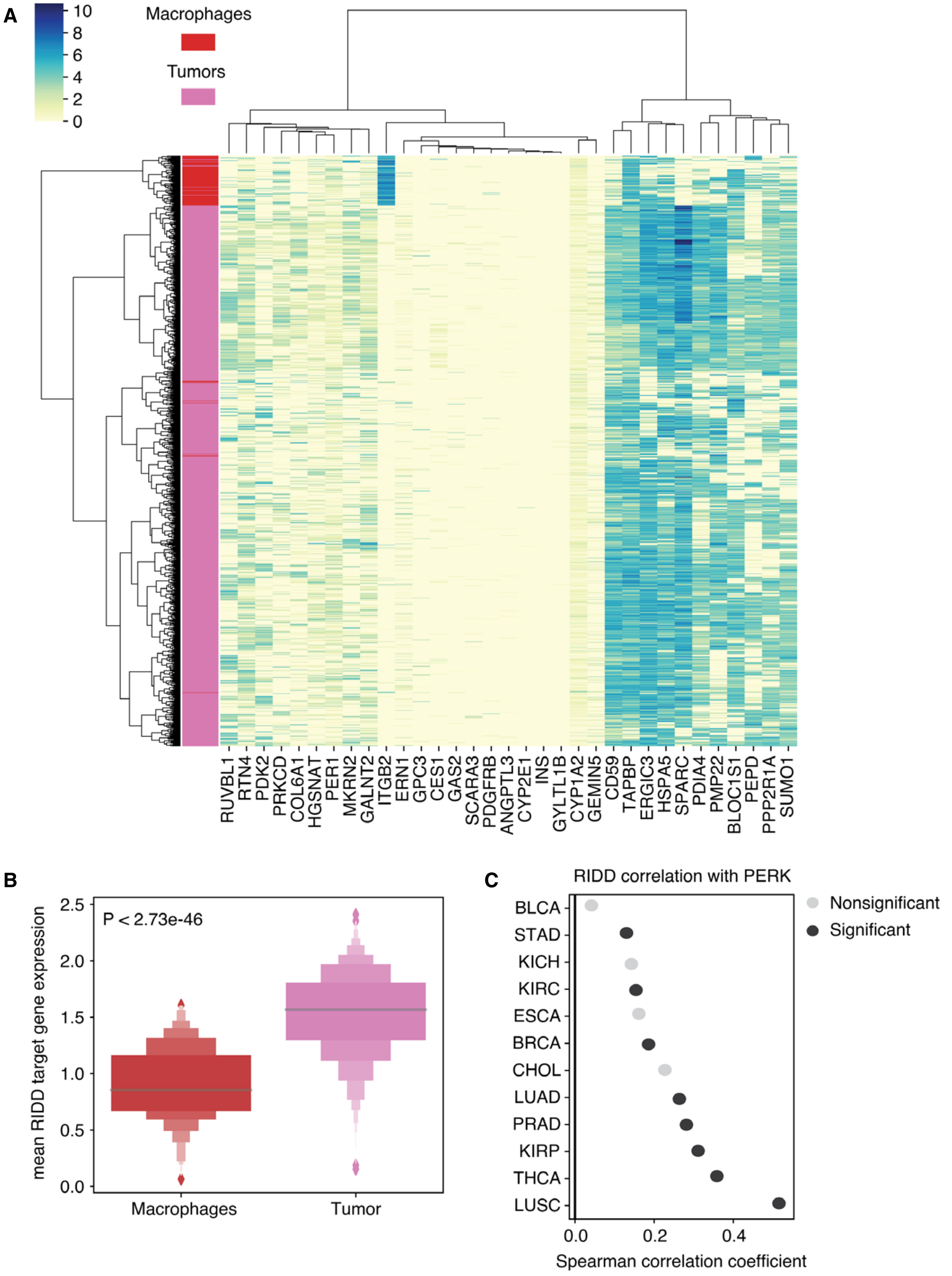

Figure EV5.

 © 2021 The Authors
