# Supplementary material for: The unfolded protein response links tumor aneuploidy to local immune dysregulation
Source: EMBO Rep. 2021 Oct 26;22(12):e52509. doi: 10.15252/embr.202152509 (PMC8647024; doi:10.15252/embr.202152509)
Supplement: Supplementary file 7 [file EMBR-22-e52509-s003.pdf]

EMBO reports

The unfolded protein response links tumor aneuploidy to local immune dysregulation

Su Xian, Magalie Dosset, Gonzalo Almanza, Stephen Searles, Paras Sahani, T Cameron Waller, Kristen Jepsen, Hannah Carter, and Maurizio Zanetti
DOI: 10.15252/embr.202152509

Corresponding author(s): Maurizio Zanetti (mzanetti@health.ucsd.edu) , Hannah Carter (hkcarter@ucsd.edu)

Review Timeline:

Submission Date:	27th Jan 21
Editorial Decision:	9th Mar 21
Revision Received:	9th Aug 21
Editorial Decision:	10th Sep 21
Revision Received:	14th Sep 21
Accepted:	22nd Sep 21

Editor: Martina Rembold

Transaction Report:

(Note: With the exception of the correction of typographical or spelling errors that could be a source of ambiguity, letters and reports are not edited. Depending on transfer agreements, referee reports obtained elsewhere may or may not be included in this compilation. Referee reports are anonymous unless the Referee chooses to sign their reports.)

2nd Editorial Decision 9th Mar 2021

Dear Dr. Zanetti

Thank you for the submission of your research manuscript to our journal. We have now received the two enclosed reports on it.

As you will see, the referees acknowledge that the findings are potentially interesting. However, referees 1 and 2 also point out several technical concerns and have a number of suggestions for how the study should be strengthened, and I think that all of them should be addressed. In particular, both referees point out that the link between the UPR and SCNA is largely correlative and that copy number changes leading to altered gene expression (referee 1) and the mutational status (referee 2) need to be considered, also experimentally. The functional work should be expanded by using additional cell lines, reversine concentrations or other drugs and the involvement of the other UPR branches should be considered.

Given these constructive comments, we would like to invite you to revise your manuscript with the understanding that the referee concerns (as detailed above and in their reports) must be fully addressed and their suggestions taken on board. Please address all referee concerns in a complete point-by-point response. Acceptance of the manuscript will depend on a positive outcome of a second round of review. It is EMBO reports policy to allow a single round of revision only and acceptance or rejection of the manuscript will therefore depend on the completeness of your responses included in the next, final version of the manuscript.

We invite you to submit your manuscript within three months of a request for revision. This would be June 9th in your case. However, we are aware of the fact that many laboratories are not fully functional due to COVID-19 related shutdowns and we have therefore extended the revision time for all research manuscripts under our scooping protection to allow for the extra time required to address essential experimental issues. Please contact us to discuss the time needed and the revisions further.

****
IMPORTANT NOTE: we perform an initial quality control of all revised manuscripts before re-review. Your manuscript will FAIL this control and the handling will be DELAYED if the following APPLIES:

1) A data availability section is missing.
2) Your manuscript contains error bars based on n=2. Please use scatter blots showing the individual datapoints in these cases. The use of statistical tests needs to be justified.

When submitting your revised manuscript, please carefully review the instructions that follow below. Failure to include requested items will delay the evaluation of your revision.
****

When submitting your revised manuscript, we will require:

1) a .docx formatted version of the manuscript text (including legends for main figures, EV figures and tables). Please make sure that the changes are highlighted to be clearly visible.

2) individual production quality figure files as .eps, .tif, .jpg (one file per figure).
Please download our Figure Preparation Guidelines (figure preparation pdf) from our Author Guidelines pages https://www.embopress.org/page/journal/14693178/authorguide for more info on how to prepare your figures.

3) a .docx formatted letter INCLUDING the reviewers' reports and your detailed point-by-point responses to their comments. As part of the EMBO Press transparent editorial process, the point-by-point response is part of the Review Process File (RPF), which will be published alongside your paper.

4) a complete author checklist, which you can download from our author guidelines (). Please insert information in the checklist that is also reflected in the manuscript. The completed author checklist will also be part of the RPF.

5) Please note that all corresponding authors are required to supply an ORCID ID for their name upon submission of a revised manuscript (). Please find instructions on how to link your ORCID ID to your account in our manuscript tracking system in our Author guidelines
()

6) We replaced Supplementary Information with Expanded View (EV) Figures and Tables that are collapsible/expandable online. A maximum of 5 EV Figures can be typeset. EV Figures should be cited as 'Figure EV1, Figure EV2" etc... in the text and their respective legends should be included in the main text after the legends of regular figures.

- For the figures that you do NOT wish to display as Expanded View figures, they should be bundled together with their legends in a single PDF file called *Appendix*, which should start with a short Table of Content. Appendix figures should be referred to in

the main text as: "Appendix Figure S1, Appendix Figure S2" etc. See detailed instructions regarding expanded view here:

- Additional Tables/Datasets should be labeled and referred to as Table EV1, Dataset EV1, etc. Legends have to be provided in a separate tab in case of .xls files. Alternatively, the legend can be supplied as a separate text file (README) and zipped together with the Table/Dataset file.

7) Please note that a Data Availability section at the end of Materials and Methods is now mandatory. In case you have no data that requires deposition in a public database, please state so instead of refereeing to the database.
See also < https://www.embopress.org/page/journal/14693178/authorguide#dataavailability>). Please note that the Data Availability Section is restricted to new primary data that are part of this study.

8) We would also encourage you to include the source data for figure panels that show essential data. Numerical data should be provided as individual .xls or .csv files (including a tab describing the data). For blots or microscopy, uncropped images should be submitted (using a zip archive if multiple images need to be supplied for one panel). Additional information on source data and instruction on how to label the files are available .

9) Our journal encourages inclusion of *data citations in the reference list* to directly cite datasets that were re-used and obtained from public databases. Data citations in the article text are distinct from normal bibliographical citations and should directly link to the database records from which the data can be accessed. In the main text, data citations are formatted as follows: "Data ref: Smith et al, 2001" or "Data ref: NCBI Sequence Read Archive PRJNA342805, 2017". In the Reference list, data citations must be labeled with "[DATASET]". A data reference must provide the database name, accession number/identifiers and a resolvable link to the landing page from which the data can be accessed at the end of the reference. Further instructions are available at .

10) Regarding data quantification
The following points must be specified in each figure legend:
- the name of the statistical test used to generate error bars and P values,
- the number (n) of independent experiments (please specify technical or biological replicates) underlying each data point,
- the nature of the bars and error bars (s.d., s.e.m.)
Discussion of statistical methodology can be reported in the materials and methods section, but figure legends should contain a basic description of n, P and the test applied.
- Please also include scale bars in all microscopy images.

11) As part of the EMBO publication's Transparent Editorial Process, EMBO reports publishes online a Review Process File to accompany accepted manuscripts. This File will be published in conjunction with your paper and will include the referee reports, your point-by-point response and all pertinent correspondence relating to the manuscript.

You are able to opt out of this by letting the editorial office know (emboreports@embo.org). If you do opt out, the Review Process File link will point to the following statement: "No Review Process File is available with this article, as the authors have chosen not to make the review process public in this case."

We would also welcome the submission of cover suggestions, or motifs to be used by our Graphics Illustrator in designing a cover.

I look forward to seeing a revised version of your manuscript when it is ready. Please let me know if you have questions or comments regarding the revision.

Yours sincerely,

Martina Rembold, PhD
Senior Editor
EMBO reports

****

Referee #1:

The unfolded protein response links tumor aneuploidy to local immune dysregulation
Su Xian, Stephen Searles, Paras Sahani, T. Cameron Waller, Kristen Jepsen, Hannah Carter and Maurizio Zanetti

In this manuscript, Xian et al suggest that the unfolded protein response (UPR) connects aneuploidy to the disruption of local immunity in multiple cancer types and contributes to immune evasion. They suggest that the aneuploidy-generated UPR is a

new variable linking cancer and immunity. The authors propose an interesting model whereby two seemingly distinct UPR pathways work together to oppose local immune surveillance which is achieved through reduced cytolytic activity of intratumor T cells and also dysregulation of macrophages. Interestingly, they also demonstrate that trans regulation of myeloid immune cells by aneuploid cancer cells is dependent on IRE1-XBP1. However much of the analysis is restricted by limited data analyses, poor statistical assessments with weak significance and incomplete figure descriptions hindering solid conclusions to be made. These issues will need to be improved prior to publication in EMBO journal.

Major comments

1. Considering the pan cancer nature of this study, the frequencies of chromosome, arm and focal SCNA should be described more extensively, highlighting how these estimates vary between cancer types. How is the data in figure 1D meant to be interpreted, how is it normalised/adjusted. Many of the figures lack statistical analysis and legends are insufficient to determine the conclusion. No scale is shown to indicate what the intensities indicate.

2. The negative correlation between p53 activity and SCNA scores is only evident in half the tumour types. Clearly additional factors influence the tolerance of SCNAs. The authors should extend this analysis and dissect out whether these differences are attributed to the prevalence of specific types of SCNA or attempt to derive additional SCNA tolerance mechanisms from their data. Alternatively, the p53 activity score could be redefined to better reflect samples where p53 pathway defects are allowing tolerance of SCNAs.

3. In Figure 2, the authors suggest a significant negative correlation between SCNA and CYT. Firstly, can the authors speculate as to why this correlation is positive in LGG and THYM? Is this because of a different type or SCNA in these two cancer types? This finding suggests again that additional factors are not being considered which is not surprising. Secondly, what is the cause of the reduced SCNA at stage IV compared to stage III. Finally, why is the correlation not significant in stage III when SCNA levels are maximal?

4. On a similar note, it would be interesting to extend this analysis to look at the level of instability in these tumours to see if this correlates better with the CYT score than their aneuploidy score.

5. As the manuscript is currently written, the link between SCNA and the UPR is not intuitive. The text would benefit from a more thorough citing of relevant papers, which would serve to make the flow more logical.

6. In Figure 3B, this link between UPR and SCNA is again largely correlative, n numbers are small and many of the correlations are not significant (or stats not even shown) which makes it very difficult to ascertain a link between the SCNA scores and UPR pathways. Furthermore, in correlating the SCNA level with the gene expression profiles, it is not clear what expression normalisation/adjustments have been done to factor in the contribution of copy number changes leading to altered gene expression, independent of the UPR. Furthermore, what was the rationale for switching to 30% and 70% quantiles for figure 3C? Can this data be plotted using similar correlations as was used in figure 3B?

7. The coordinated activity of UPR proteins on page 19 in relation to figure 4 is conceptually interesting and is a key finding, but the section is poorly written again making it difficult to determine the contribution of the co expression relationships. Furthermore, has the specificity of this analysis this been controlled for by assessing other pairs of genes unrelated to the UPR?

8. Considering that this paper is largely correlative, the functional work needs to be more extensive. With the very different response kinetics to reversine in the two tested cell lines and the excessively high concentrations of reversine used, this analysis could benefit from using additional cell lines, lower reversine doses that are sufficient to inhibit mps1 or additional drugs. Alternatively, an additional readout of UPR in the fusion experiments could suffice as this spicing XBP1 readout seems not very sensitive.

Minor comments

In Supplementary figure 2A, this result could be interpreted as p53 tolerating SCNA rather than having a protective role as described

In figure 7D, for DLD1 and SKOV cell lines, please include triplicate experiments.

Referee #2:

General comments
In their study the authors highlight that the UPR constitutes a link between tumour aneuploidy and local immune dysregulation. To do so, the authors first highlight the inverse correlation between a somatic copy-number alteration (SCNA)/aneuploidy score

(which increases with tumour stage) and a cytotoxicity score (granzyme-perforin expression) in TCGA data across 23 different tumour types. They further report a correlation between SCNA score and expression of genes downstream of UPR sensors. The authors also highlight UPR target gene pairs which co-expression is regulated by the SCNA score. They also report that the activation of the PERK and IRE1 (via RIDD) branches negatively correlates with the cytotoxicity score and positively correlate with SCNA score. Using Reversine and cell-cell fusions, the authors also report that aneuploidy induces XBP1 splicing. Lastly, the authors describe that the transfer of aneuploid cells CM to BMDMs promotes production of cytokines like IL6 or Arg1. I think several points need to be experimentally addressed to allow the authors to draw their current conclusions, as detailed below.
Aneuploidy has been linked in many instances to proteotoxic stress as illustrated previously by the seminal work of Angelika Amon. Conceptually the work presented in this manuscript is of interest but remains very correlative. In addition, the mutational burden is not considered at all in the conclusions raised by this manuscript and this could also be a cause of UPR activation and immune landscape modulation. As such the authors should find a way to deconvolute both aspects in their in silico approach first and second experimentally. Regarding the latter, the cellular models developed in the Amon lab could be considered as relevant tools.
Defining the link that the UPR (and the specific branches of the UPR) could constitute between tumour cell aneuploidy and immune microenvironment modulation is an interesting topic. However, some of the results are a bit over-interpreted and further experiments are needed for the authors to draw their current conclusions. The first 5 figures remain somehow correlative. The two last figures, which aim at proving the link identified through these correlations, need to be improved. In particular, the authors should explore the activation of the other UPR branches (i.e RIDD and PERK) in Figure 6. The authors should also in Figure 7 investigate whether the XBP1s, the RIDD and/or the PERK branches in the aneuploid cells might be involved in defining the BMDMs secretome content. Additional points are detailed in the letter to the authors

Specific comments
Introduction: the following sentence on page 4 seems misplaced as the authors have not introduced the UPR at this point of the intro: With the issue remaining largely unresolved, we decided to test the hypothesis that the unfolded protein response (UPR) could serve as the link between cancer cell aneuploidy and immune cells. It might be better placed after line 12 on the same page.

The gene "signatures" used in this study remain very superficial as now several studies have identified complex molecular signatures reflecting PERK, IRE1 or ATF6 activity, As such the authors should use such tools to validate their correlation. In particular the authors used the expression of EIF2AK3, ERN1 and ATF6 mRNA as relevant predictors which they are not. The use of Reactome for the selection of UPR genes should have been complemented with UPR target genes found in other reference databases (MGI, GO) to provide an exhaustive view of the pathway.

RIDD target genes used in the manuscript are those described by Maurel et al in 2014, however, even though this list constituted a first repository of mRNA found to be cleaved by IRE1 in various (and independent experimental settings), the authors should definitely refine this list for the purpose of their study. Many reports, since Maurel et al, identified new RIDD targets, and RIDD is very variable from one cell/tissue type to one other.

Figure 4D: I am not sure the annotations are correct, these should be checked again by the authors. For example, HERPUD1 is depicted in blue (PERK pathway) even though it is an ATF6 target.

How do the authors differentiate the ISR from the UPR, especially regarding the PERK pathway. For instance, what is the activation status of PKR or GCN2 in the tumors studied. This point should be specifically and experimentally addressed.

Page 24. The authors state that "the fact that RIDD and PERK have a similar relationship to CYT is not surprising since RIDD activity was shown to be dependent (Moore and Hollien, 2015). How general is really this statement (does it apply to all RIDD substrates)? From the finding that the RIDD and PERK pathway score correlate in 8 out of 12 tumors, the authors conclude that "PERK and RIDD exert a negative effect on immune cells in the tumor microenvironment cooperatively". This reads like an over-interpretation of this correlation and needs to be toned down here. RIDD activation was not associated with macrophage recruitment in brain tumor models, how do the authors reconcile their data with this observation?

Figure 6: Is the PERK branch (what is the eif2a phosphorylation status?; what is the PKR activation status) also regulated in these conditions? Same question for the RIDD. Moreover the secretome produced by those cells should be determined quantitatively.

Figure 7: Is there also a direct effect of CM from aneuploid cells on T lymphocytes? Is there a role for the PERK branch here? Is the activity of IRE1 (XBP1 splicing or RIDD) and/or PERK in the aneuploid cells required for the effect observed on BMDMs? RIDD activity should be quantified.

In the models tested, the authors should also differentiate aneuploidy-associated proteotoxicity from UPR signalling. In their models, what is the impact of antiproliferation compounds such as AICAR or 17AAG on the activation of the UPR and on the production of an immunomodulating secretome?

At last, aneuploidy has been linked to resistance to chemotherapeutics in cancer cells, is that validated in the cancer data used

by the authors? And if yes, how does the UPR relate to this phenotype?

Referee #1:

The unfolded protein response links tumor aneuploidy to local immune dysregulation
Su Xian, Stephen Searles, Paras Sahani, T. Cameron Waller, Kristen Jepsen, Hannah Carter and Maurizio Zanetti

In this manuscript, Xian et al suggest that the unfolded protein response (UPR) connects aneuploidy to the disruption of local immunity in multiple cancer types and contributes to immune evasion. They suggest that the aneuploidy-generated UPR is a new variable linking cancer and immunity. The authors propose an interesting model whereby two seemingly distinct UPR pathways work together to oppose local immune surveillance which is achieved through reduced cytolytic activity of intratumor T cells and also dysregulation of macrophages. Interestingly, they also demonstrate that trans regulation of myeloid immune cells by aneuploid cancer cells is dependent on IRE1-XBP1. However much of the analysis is restricted by limited data analyses, poor statistical assessments with weak significance and incomplete figure descriptions hindering solid conclusions to be made. These issues will need to be improved prior to publication in EMBO journal.

We thank the reviewer for the helpful suggestions for improving the clarity and quality of our manuscript.

Major comments

1. Considering the pan cancer nature of this study, the frequencies of chromosome, arm and focal SCNA should be described more extensively, highlighting how these estimates vary between cancer types. How is the data in Figure 1D meant to be interpreted, how is it normalised/adjusted. Many of the figures lack statistical analysis and legends are insufficient to determine the conclusion. No scale is shown to indicate what the intensities indicate.

We sought to improve Figure 1 and its description to better convey how the frequencies of chromosome, arm and focal SCNAs vary between cancer types. **Figure 1D** shows the distribution of the number of SCNA events across tumors grouped by tumor type and SCNA category. We have modified the figure to mimic similar plots as are used to show tumor mutation burden distribution across tumors for each cancer type. We also improved the axis labels and added the missing color bar for **Figure 1C**.

We revised the text describing Figure 1D as follows to improve clarity:

"We further compared the number of each SCNA category per tumor across the thirty-two tumor types (Fig. 1D)."

We also updated the Figure 1 caption to more clearly describe what is shown:

"**Figure 1. Characterizing aneuploidy across 32 tumor types**.
(**A**) An illustration on the definition of focal, arm, and chromosome level somatic copy-number alterations (SCNAs).
(**B**) Example of somatic copy-number alterations detected in a single TCGA sample (TCGA-02-0003, GBM) with copy number gain in red and copy number loss in blue. The x axis represents the chromosomal location, and the y axis shows the $\log_2$ fold change in the intensity of the corresponding region relative to diploid.
(**C**) Heatmap showing the average fold change of copy number gain (red) and loss (blue) of chromosomal p and q arm events across 32 tumor types in TCGA.
(**D**) Visualization of the distribution of total SCNA events (loss and gain) by category across tumors grouped by 32 tumor types in TCGA. From top to bottom, plots depict events of chromosomal, arm, and focal level SCNA respectively."

As suggested by the reviewer, we revised all of our figures and figure captions to ensure that all axes are labeled, legends are present, figure panels are clearly explained, and that statistics are included where appropriate.

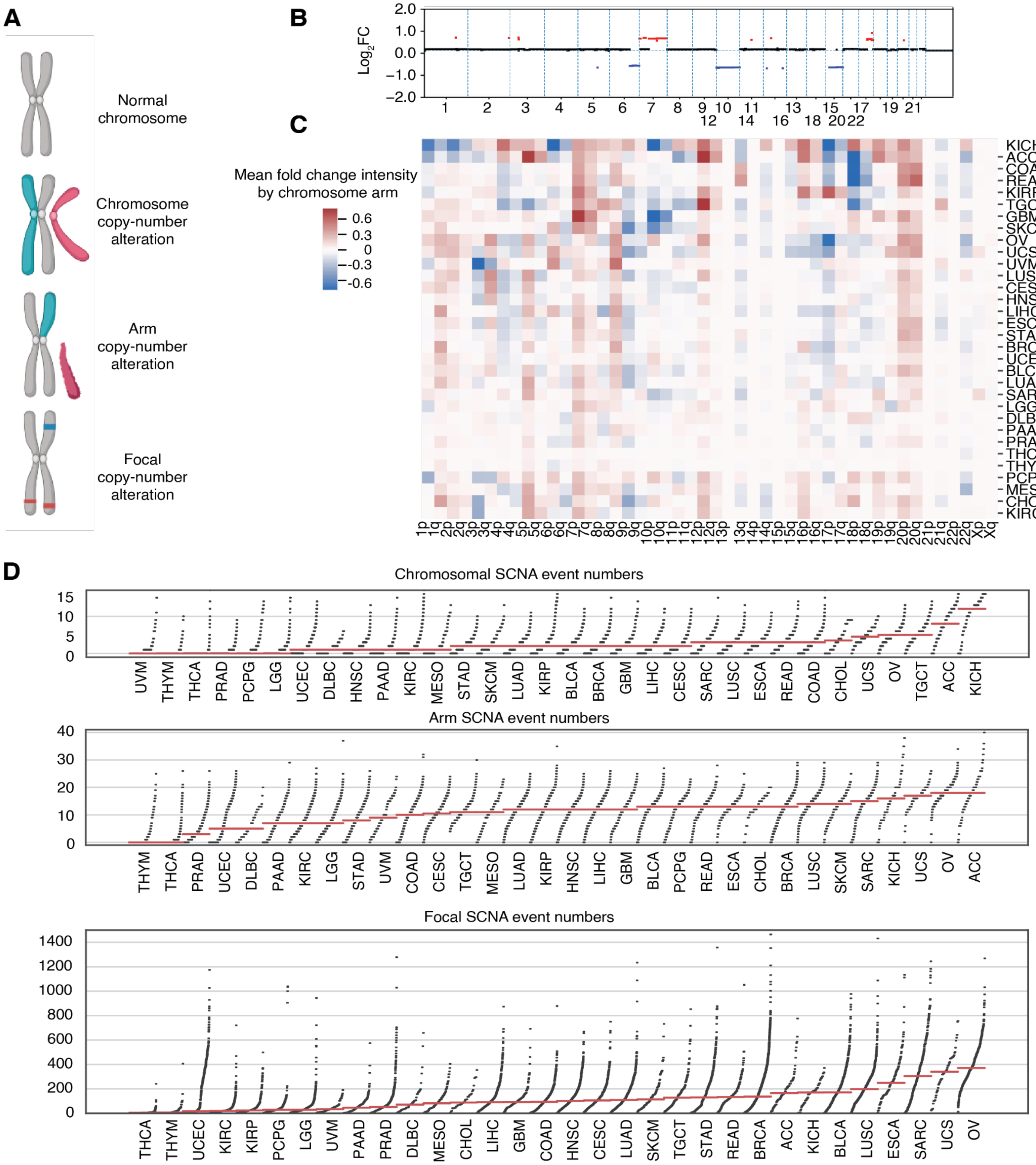

Updated Figure 1.

2. The negative correlation between p53 activity and SCNA scores is only evident in half the tumour types. Clearly additional factors influence the tolerance of SCNAs. The authors should extend this analysis and dissect out whether these differences are attributed to the prevalence of specific types of SCNA or attempt to derive additional SCNA tolerance mechanisms from their data. Alternatively, the p53 activity score could be redefined to better reflect samples where p53 pathway defects are allowing tolerance of SCNAs.

We thank the reviewer for this comment. Other studies have more thoroughly addressed tolerance mechanisms that allow tumor cells to accumulate SCNAs. For example, somatic mutations in genes regulating apoptosis are associated with higher levels of aneuploidy in many tumor types [1]. Our work is more focused on the downstream consequences of high SCNA burden with focus on the unfolded protein response and reduced immune activity. We did revisit p53 activity scores with respect to the 3 SCNA categories and found a significant negative correlation between TP53 activity and SCNA burden suggesting that TP53 loss is permissive of accumulation of any type of SCNA. Furthermore, evaluating the partial correlation between SCNA and CYT add P53 activity scores as covariates still show that higher SCNA levels tended to show lower CYT scores (now **Appendix Figure S4B** in the manuscript).

Table R1. Regression coefficients for focal, arm and chromosome level SCNA events in a model predicting TP53 activity score across multiple tumor types, with tumor type as a covariate.

	coefficient	standard error	t statistics	p value	ci [0.025]	ci [0.975]
focal	-0.1824	0.013	-14.113	9.08e-45	-0.208	-0.157
arm	-0.2465	0.015	-16.683	1.34e-61	-0.276	-0.218
chr	-0.0947	0.014	-6.690	2.36e-11	-0.122	-0.067

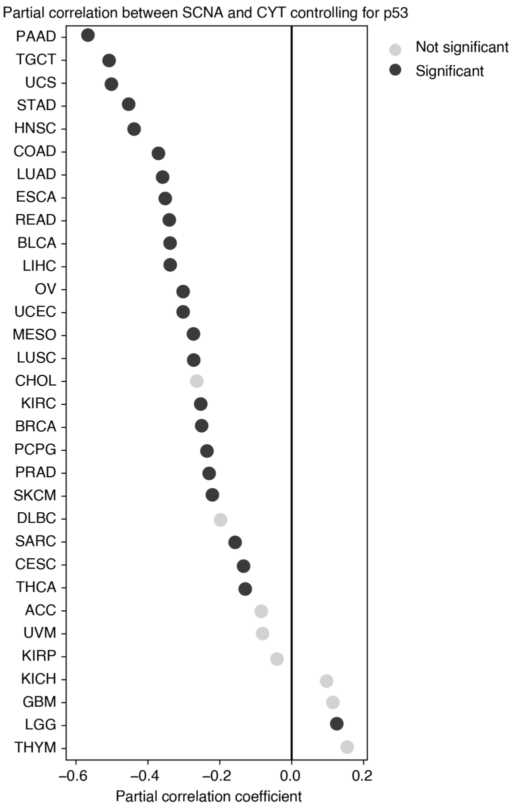

Appendix Figure S4B. SCNA level partial correlation with CYT score in tumors controlling for TP53 activity level. SCNA still associates with worse immune activity (e.g. worse CYT score) in many tumor types.

This has now been added to the main manuscript as follows:

"We computed *TP53* activity scores using ten *TP53* repressed genes [51] and found a significant negative correlation between *TP53* activity and SCNA score in seventeen out of thirty-two cancer types (Appendix Fig. S3B). Among these, only THYM showed a significant positive correlation. Across tumors, TP53 activity was negatively correlated with each of the three SCNA categories (Appendix Figure S3C)."

and

“Although we noted correlation between TP53 activity and SCNA score, the tumor-type specific correlation between SCNA score and CYT was independent of TP53 activity score by partial correlation analysis (Appendix Fig. S4B).”

3. In Figure 2, the authors suggest a significant negative correlation between SCNA and CYT. Firstly, can the authors speculate as to why this correlation is positive in LGG and THYM? Is this because of a different type or SCNA in these two cancer types? This finding suggests again that additional factors are not being considered which is not surprising.

We thank the reviewer for highlighting these outlier cases. We took a closer look at the SCNA and CYT score distributions in LGG and THYM specifically. We note that THYM had lower levels of SCNA relative to other tumor types. If we consider only THYM samples where the SCNA score is at least 0.1, there is no longer a positive correlation (**Figure R1A**). When restricting to samples with SCNA score > 1, the correlation is strongly negative but not significant due to small sample size ($r = -0.325$, $p = 0.096$). Thus for the THYM case, the positive correlation appears to be driven by samples with very few SCNAs and lower CYT scores. This could suggest that alternative mechanisms of immune evasion are at play in this disease.

Thymoma represent rare tumors of the thymus, mainly of TEC (epithelial cells), and are traditionally linked with autoimmunity, including myasthenia gravis, lupus erythematosus, thyroiditis and aplastic anemia [2]. A plausible interpretation of this association is a dysregulation of T cell immunity with defective negative selection, loss of Tregs, and malfunction of AIRE. Significantly, a few reports have focused on the characteristics of T cell immunity in thymoma patients. In one report it was found that the $CD8^+$ but not $CD4^+$ subset among $CD45RA^+$ T cells is increased in the blood of patients with thymoma suggesting a potential role of $CD8^+$ T cells as mediators of autoimmunity [3]. Another report showed that patients with thymoma and hypopituitarism have anti-pituitary antibodies but also CTL specific for the PIT-1 antigen [4].
Based on these reports we suggest that in thymoma a dysregulation of the T cell compartment is likely complicated by the occurrence of self-reactive CD8 T cells that may result in variable levels of CYT in samples independent of SCNA status.

The trend toward positive correlation between CYT and SCNA in LGG and GBM may reflect the reduced overall infiltration of immune cells into the brain in general. **Figure R1B** shows that LGG samples have low levels of CYT in general, consistent with reports that these tumors have particularly low CD8 T cell infiltration, even more so than GBM [5]. Higher levels of immune cell infiltration have also been associated with worse prognosis in brain tumors [6]. Together, these observations suggest that brain tumors have less need to evade the immune system, but when T cells do make it into the tumor, SCNAs could still provide a means to escape.

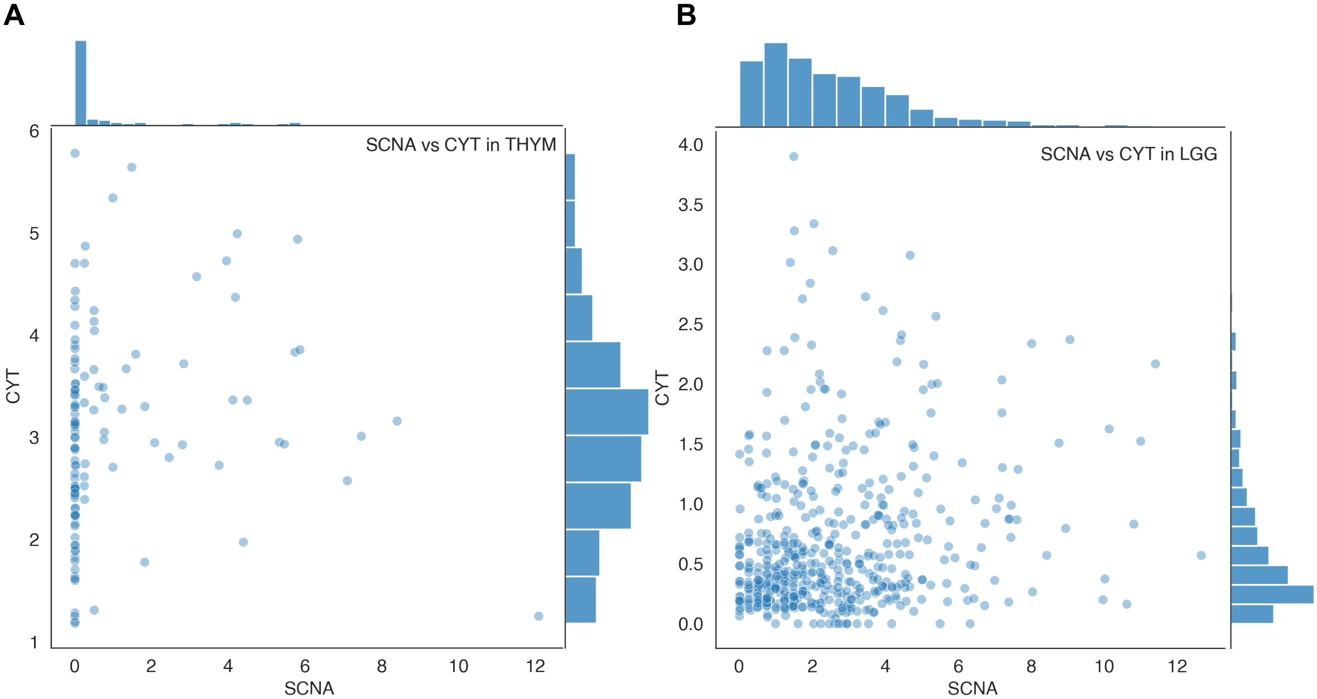

Figure R1. A) The relation between CYT score and SCNA burden in THYM. B) The relation between CYT score and SCNA burden in LGG.

We have added the following text to the manuscript discussing these cases as suggested by the reviewer:

"We note that THYM had lower SCNA scores than other tumor types, and when we restricted analysis to THYM tumors with an SCNA score > 1, there was a trend toward inverse correlation between SCNA score and CYT (r = -0.325, p = 0.096). In low SCNA THYM tumors, other factors appear to influence CYT levels, which may reflect the association of THYM with autoimmunity [54] and altered immune surveillance and potentially promoting alternative mechanisms immune evasion and increased cytotoxic T cells [55, 56]. Trends in LGG and GBM may reflect differences in the tumor microenvironment in the brain; LGG in particular is characterized by low levels of CD8 T cell infiltration [57]."

Secondly, what is the cause of the reduced SCNA at stage IV compared to stage III. Finally, why is the correlation not significant in stage III when SCNA levels are maximal?

SCNA levels are likely highest in stage III because the majority of high-grade ovarian serous carcinomas (OV) are stage III (new **Appendix Figure S4A**) and these tend to have a high level of chromosomal aberrations. Sample sizes for stage IV are also smaller than for other stages. For this reason, we used OLS regression with tumor type as a covariate to further verify that SCNA scores are progressively higher across tumors, and that CYT values fall. This was accomplished by evaluating these values relative to stage I. Although the drop in CYT at stage three was not significant relative to stage I, we still see a strong inverse correlation between SCNAs and CYT score and this holds in multiple tumor types (**Figure R2**). We have sought to further clarify this analysis and its interpretation as follows:

"We defined separate models to predict SCNA scores and CYT scores from tumor stage, using Stage I as a baseline for comparison (Table 1). SCNA scores were significantly higher in stages II-IV relative to stage I (p= 1.39e-09, p= 3.77e-10, p= 2.01e-11) (Table EV1). For CYT, we observed a near significant negative coefficient for Stage II (p = 0.075) and a significant negative coefficient for Stage IV (p = 9.74e-5) (Table 1), but no significant reduction in stage III relative to stage I. Nonetheless, we observed a significant inverse correlation between CYT score and SCNA score in all stages (Fig 2C; Table 1)."

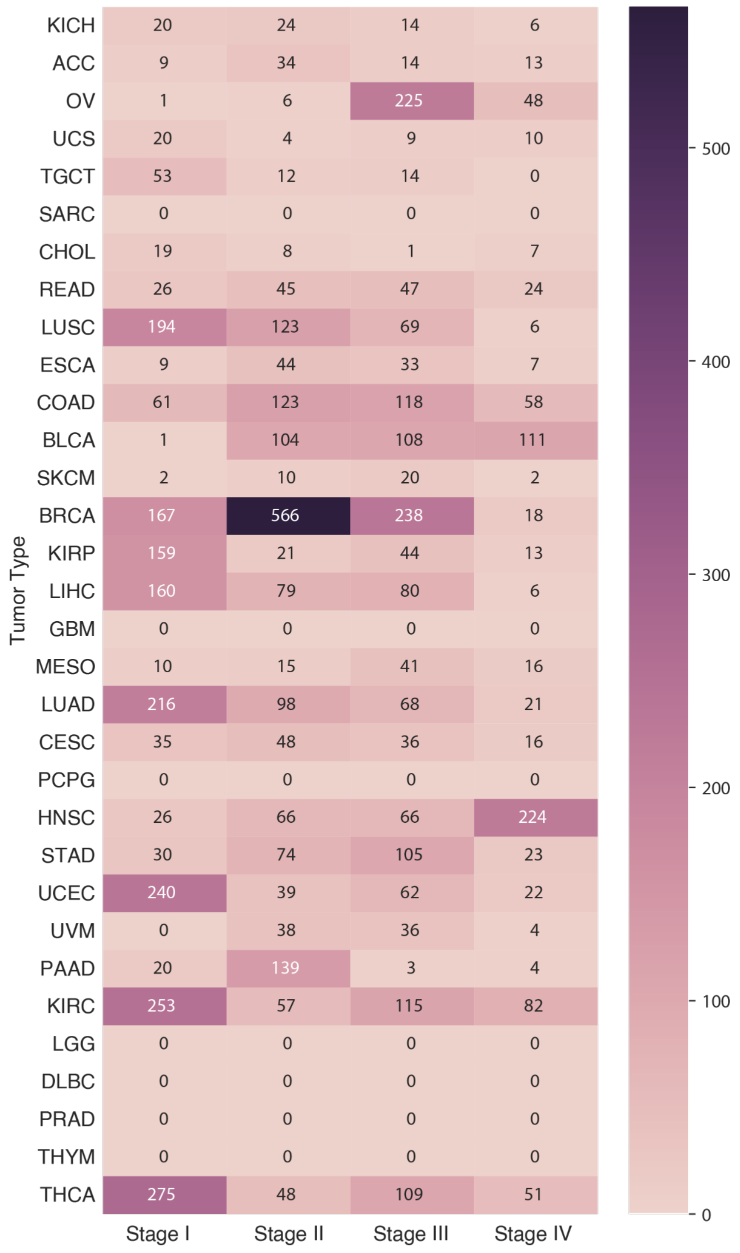

Appendix Figure S4A. Distribution of samples by tumor type across stages in TCGA.

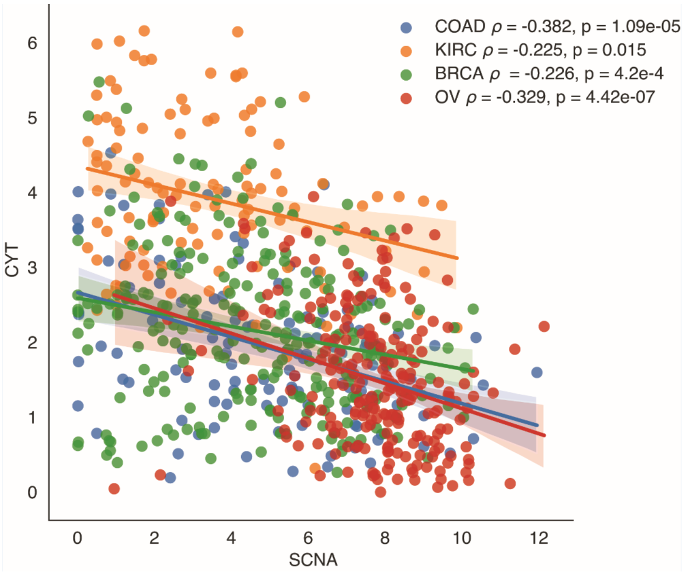

Figure R2. Relationship between CYT score and SCNA level in the four most abundant stage 3 tumors. Correlation values: OV correlation = -0.329, p-value = 4.42e-07; BRCA correlation = -0.226, p-value = 0.00042; COAD correlation = -0.382, p-value = 1.09e-05; KIRC correlation = -0.225, p-value = 0.015

4. On a similar note, it would be interesting to extend this analysis to look at the level of instability in these tumours to see if this correlates better with the CYT score than their aneuploidy score.

We thank the reviewer for this suggestion. We evaluated chromosomal instability (CIN) scores from [7] and also levels of microsatellite instability (MSI) quantified by the MANTIS score [8] for all tumors in our data set. Neither CIN nor MANTIS score correlated as consistently with CYT as the SCNA score (**Figure R3**). As expected, the MANTIS score showed positive association with CYT in several tumor types where high MSI tumors are more common (COAD, READ, UCEC). We evaluated the relationship between each score and CYT score using a linear model: CYT ~ instability score + tumor type. Using the AIC criterion to compare models, the model using SCNA scores provided the best explanation of the data; the CIN and MANTIS based models were 6.84e-109 and 1.84e-137 times as probable as the SCNA model to minimize information loss.

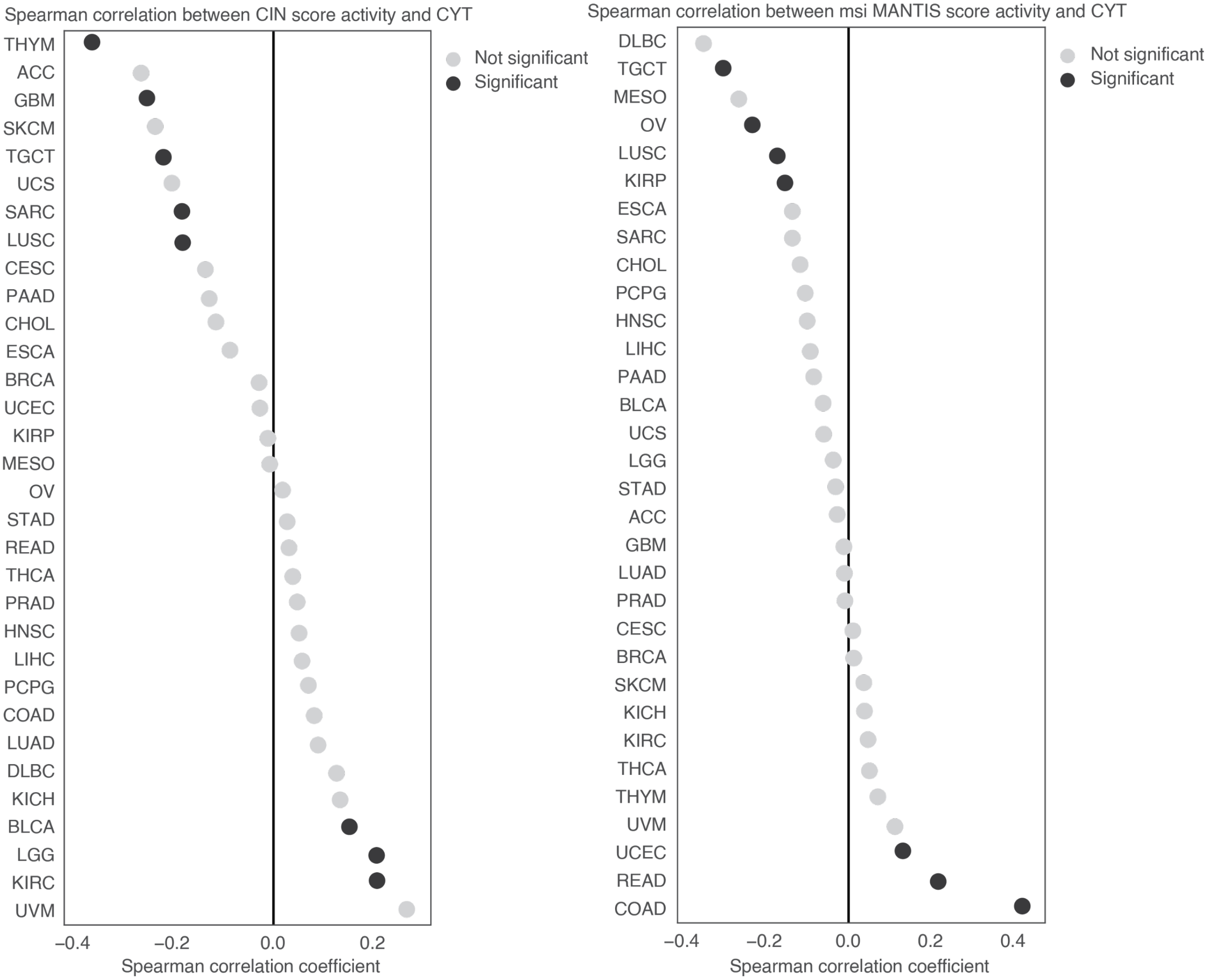

Figure R3. Correlation between alternative measures of genomic stability and cytolytic activity across cancer types.

5. As the manuscript is currently written, the link between SCNA and the UPR is not intuitive. The text would benefit from a more thorough citing of relevant papers, which would serve to make the flow more logical.

To address this comment, we significantly revised our introduction, reordering ideas and adding additional citations to make the link between SCNA and UPR more logical.

6. In Figure 3B, this link between UPR and SCNA is again largely correlative, n numbers are small and many of the correlations are not significant (or stats not even shown) which makes it very difficult to ascertain a link between the SCNA scores and UPR pathways. Furthermore, in correlating the SCNA level with the gene expression profiles, it is not clear what expression normalisation/adjustments have been done to factor in the contribution of copy number changes leading to altered gene expression, independent of the UPR. Furthermore, what was the rationale for switching to 30% and 70% quantiles for figure 3C? Can this data be plotted using similar correlations as was used in figure 3B?

We thank the reviewer for these suggestions. We sought to improve the consistency and clarity of the analysis presented in **Figure 3B-C**. We revised 3B to more clearly distinguish which correlations were significant. We also updated **Figure 3C** to now display significant correlations/ inverse correlations between SCNAs and gene

expression instead of log2 FC in gene expression between high and low SCNA tertiles. We further evaluated whether the expression of the UPR genes was still correlated with SCNA levels after accounting for direct effects of copy number changes to genes in the UPR pathway using partial correlation analysis. In short, we evaluated correlation between the regression residuals of models regressing gene expression with copy number, and SCNA with copy number. Intuitively, this corresponds to subtracting correlation with copy number from SCNA and gene expression separately, then checking whether there is still a correlation between SCNA and gene expression. We have updated **Figure 3C** to show the partial correlations and modified the manuscript text as follows:

"We then compared the expression of these genes in each UPR branch in tumor samples with SCNA score using partial correlation analysis to account for contributions of amplifications or deletions affecting each gene (Fig. 3C). "

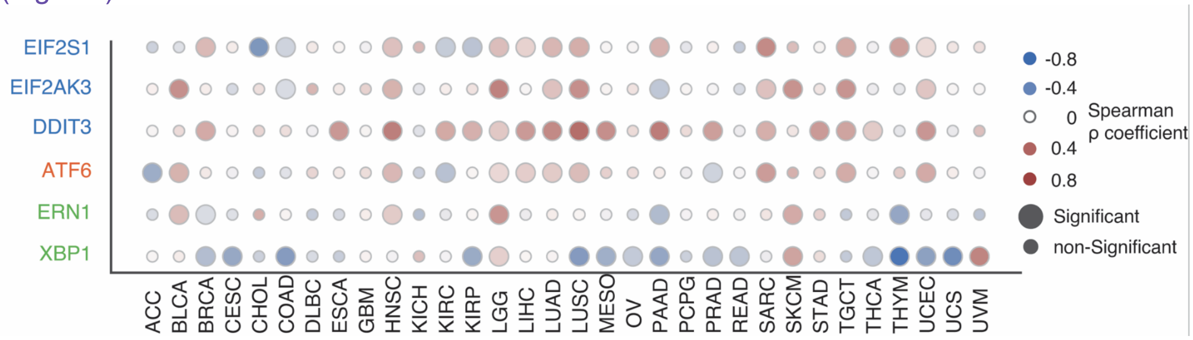

Updated Figure 3B: Our revised Figure 3B now shows statistical significance of Spearman correlations between SCNA and gene expression level using size to illustrate relationships passing a multiple testing corrected significance threshold of 0.05.

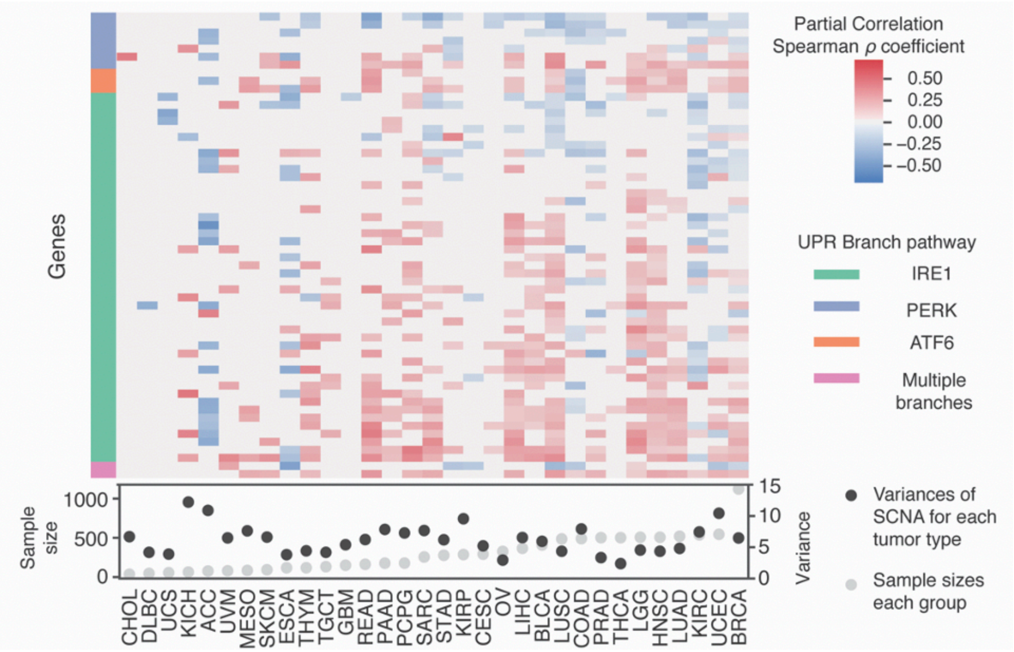

Updated Figure 3C: Revised Fig. 3C now shows correlation/ inverse correlation between UPR pathway gene expression and SCNA level in order to be consistent with Figure 3B. Only correlations significant after multiple testing correction are shown. Non-significant correlations are set to 0.

7. The coordinated activity of UPR proteins on page 19 in relation to figure 4 is conceptually interesting and is a key finding, but the section is poorly written again making it difficult to determine the contribution of the co expression relationships. Furthermore, has the specificity of this analysis this been controlled for by assessing other pairs of genes unrelated to the UPR?

We have revised this section for clarity and compared to some alternative gene sets to aid interpretation. In our initial analysis, we used randomization of SCNA levels within the geneset to generate an empirical null against which we assessed whether genes were more or less co-expressed than expected. To assess specificity, the ideal control would be a pathway not under selection during tumor development and thus would represent the random effects of SCNAs on co-expression patterns. A random sample of genes would not show a functionally relevant pattern of co-expression at baseline. While it is impossible to be sure that none of the genes in a pathway are under selection in tumor progression, we identified several pathways that seemed likely irrelevant to tumorigenesis, including the cardiac conduction pathway plateau phase(R-HSA-5576893.2), the olfactory signaling pathway(R-HSA-381753.3) and the visual phototransduction pathway(R-HSA-2187338.1). We also selected several known oncogenic pathways including TP53(R-HSA-3700989.6), hypoxia(R-HSA-9646399.3) EGFR(R-HSA-177929.2) and MAPK(R-HSA-187687.1) (only 7 genes overlap between these definitions of EGFR and MAPK). We revisited our analysis with each of these pathways and found that the number of co-expression relationships conserved or preserved in oncogenic pathways was typically higher than those in the selected control pathways (now included in the manuscript as expandable Figure EV3). We can further use the control pathways to estimate a false discovery rate for the UPR pathway analysis by assessing how many gene pairs in the non-cancer pathway(s) would be deemed perturbed or conserved at that threshold. We did note that the most perturbed gene pair in the visual phototransduction pathway was METAP2/FNTA and these genes have both been implicated as drivers of tumor development, METAP2 in rhabdomyosarcoma [9] and FNTA in pancreatic endocrine tumor [10].

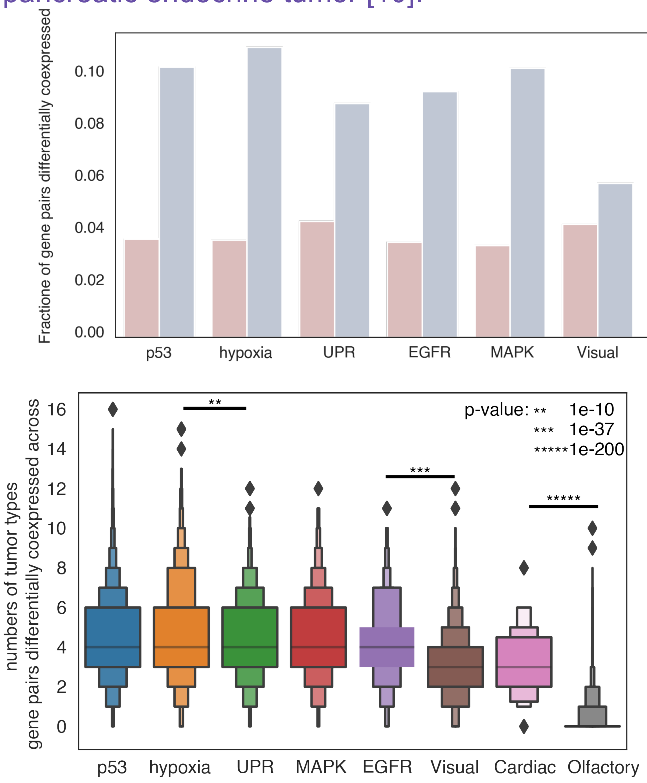

Figure EV3. Comparison of differential co-expression of gene pairs under high and low SCNA conditions across the UPR and 7 control pathways. **Top:** UPR and cancer-associated pathways show more perturbed gene pairs than non-cancer control pathways. **Bottom**: Gene pairs are more consistently perturbed across tumor types for cancer pathways than for control pathways.

As described above, the control pathways are cardiac conduction pathway plateau phase (R-HSA-5576893.2), the olfactory signaling pathway (R-HSA-381753.3) and the visual phototransduction pathway (R-HSA-2187338.1). We examined the gene pairs in each control pathway and evaluated among how many tumor types these gene pairs are differentially co-expressed. We noticed that among these control pathways, the numbers of differentially expressed gene pairs across tumor types are far less than those cancer related pathways (e.g. the t-tests reveal differences in the EGFR and visual phototransduction pathway, p = 2.4e-38). Thus, our method

is able to capture the differences between the control pathway and cancer related pathway. We also noticed that UPR pathway has a co expression change similar to MAPK and EGFR pathway. Assuming all gene pairs we identified from the control pathways are false positives, and the gene pairs in cancer pathways are true positives, we estimate the false discovery rate of our method is 0.0995 (10%). The calculation is normalized by the total number of gene pairs in control pathways and cancer pathways to avoid potential bias (with 46932/3048160 gene pairs differentially coexpressed in control pathways, 291227/2091424 gene pairs differentially coexpressed in cancer pathways, FDR is calculated as 46932/3048160 / (46932/3048160 + 291227/2091424)). This additional analysis is now included in the manuscript as follows:

"To aid interpretation, we repeated this analysis for three oncogenic pathways (TP53, EGFR and MAPK) and three pathways selected for lack of association with tumorigenesis (Olfaction, cardiac conduction and visual phototransduction). We noted that the fraction of gene pairs perturbed in association with higher SCNA levels was consistently higher in oncogenic as compared with control pathways (Fig EV3A). Perturbations were also observed more consistently across multiple cancer types for oncogenic versus control pathways (Fig EV3B). We used the control pathways to estimate an empirical false discovery rate of 0.0995 (~10%) for detection of recurrently perturbed genes. This may be conservative as we found literature evidence that the genes in the top perturbed gene pair of the visual phototransduction control pathway, METAP2-FNTA, play roles in oncogenic progression in rhabdomyosarcoma [65] and pancreatic endocrine tumors [66], respectively."

8. Considering that this paper is largely correlative, the functional work needs to be more extensive. With the very different response kinetics to reversine in the two tested cell lines and the excessively high concentrations of reversine used, this analysis could benefit from using additional cell lines, lower reversine doses that are sufficient to inhibit mps1 or additional drugs. Alternatively, an additional readout of UPR in the fusion experiments could suffice as this splicing XBP1 readout seems not very sensitive.

We thank the reviewer for the suggestion to expand the breadth of the UPR analysis in aneuploid cells. Given the time frame we found it very difficult to procure additional quasi-diploid cancer cell lines, a requirement for the experiment. Accordingly, we performed an analysis of the three branches of the UPR by PCR and Western blotting in DLD1 and SKOV3 using reversine at the concentration determined in the titration experiments. The scope was to determine if aneuploidy triggers a more general UPR. The new data, updated **Figure 6E and F,** show a general UPR activity induced by reversine**.**

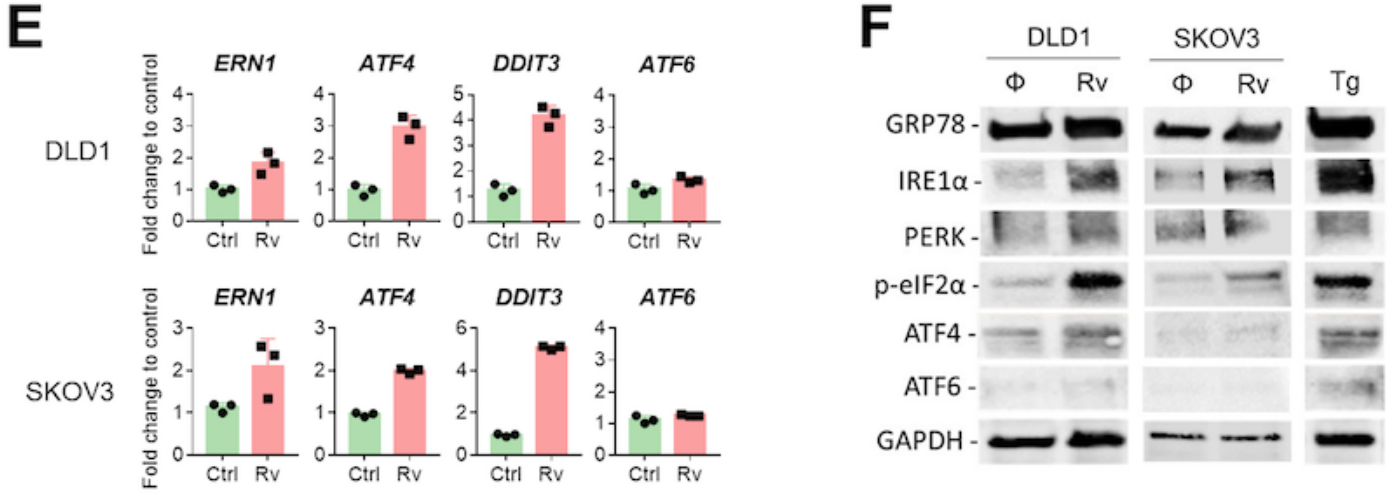

Updated Figure 6 E and F.

Summary and comment to results:

We noted an upregulation of GRP78, the master regulator of the UPR, and CHOP (*DDIT3*). Overall, both the IRE1α and the PERK branches were activated (**Fig. 6E-F**), with phosphorylation of eIF2α downstream of PERK

being clearly discernable. Together this suggests a global activation of the UPR. Oddly, we did not detect transcription or translation of ATF6. This may be interpreted as follows. (a) ATF6 activation is delayed relative to IRE1α and PERK consistent with an earlier report [11]. (b) Insufficient activation would provide a pro-survival dynamic to aneuploid cells since ATF6 is an important regulator of the pro-apoptotic factor CHOP downstream of ATF4 [12]. This interpretation is also consistent with previous work from this laboratory showing that the UPR induced through transcellular transmission results in reduced activation of ATF4 downstream of eIF2α [13]. Together, this suggests that aneuploidy-driven UPR is a homeostatic mechanism cancer cells use to avoid apoptosis and survive. (c) The magnitude of aneuploidy-driven UPR is below the threshold needed for ATF6 activation. Since ATF6 has been shown to reduce markers of stemness in epithelial colon cells [14] it can be argued that a non-involvement of ATF6 facilitates self-renewal. A caveat to this interpretation is that since ATF6 is required for the transcription of XBP1 [15] lack of ATF6 activation would serve as a rate limiting factor of XBP1 splicing.

Minor comments

In Supplementary figure 2A, this result could be interpreted as p53 tolerating SCNA rather than having a protective role as described

We have rephrased this to clarify that the results are consistent with the data presented by [16] based on which they concluded that TP53 acts to prevent chromosomal segregation errors.

Referee #2:

General comments

In their study the authors highlight that the UPR constitutes a link between tumour aneuploidy and local immune dysregulation. To do so, the authors first highlight the inverse correlation between a somatic copy-number alteration (SCNA)/aneuploidy score (which increases with tumour stage) and a cytotoxicity score (granzyme-perforin expression) in TCGA data across 23 different tumour types. They further report a correlation between SCNA score and expression of genes downstream of UPR sensors. The authors also highlight UPR target gene pairs which co-expression is regulated by the SCNA score. They also report that the activation of the PERK and IRE1 (via RIDD) branches negatively correlates with the cytotoxicity score and positively correlate with SCNA score. Using Reversine and cell-cell fusions, the authors also report that aneuploidy induces XBP1 splicing. Lastly, the authors describe that the transfer of aneuploid cells CM to BMDMs promotes production of cytokines like IL6 or Arg1. I think several points need to be experimentally addressed to allow the authors to draw their current conclusions, as detailed below.

Aneuploidy has been linked in many instances to proteotoxic stress as illustrated previously by the seminal work of Angelika Amon. Conceptually the work presented in this manuscript is of interest but remains very correlative. In addition, the mutational burden is not considered at all in the conclusions raised by this manuscript and this could also be a cause of UPR activation and immune landscape modulation. As such the authors should find a way to deconvolute both aspects in their in silico approach first and second experimentally. Regarding the latter, the cellular models developed in the Amon lab could be considered as relevant tools.

Defining the link that the UPR (and the specific branches of the UPR) could constitute between tumour cell aneuploidy and immune microenvironment modulation is an interesting topic. However, some of the results are a bit over-interpreted and further experiments are needed for the authors to draw their current conclusions. The first 5 figures remain somehow correlative. The two last figures, which aim at proving the link identified through these correlations, need to be improved. In particular, the authors should explore the activation of the other UPR branches (i.e RIDD and PERK) in Figure 6. The authors should also in Figure 7 investigate whether the XBP1s, the RIDD and/or the PERK branches in the aneuploid cells might be involved in defining the BMDMs secretome content. Additional points are detailed in the letter to the authors

Specific comments
Introduction: the following sentence on page 4 seems misplaced as the authors have not introduced the UPR at this point of the intro: With the issue remaining largely unresolved, we decided to test the hypothesis that the unfolded protein response (UPR) could serve as the link between cancer cell aneuploidy and immune cells. It might be better placed after line 12 on the same page.

We agree with this and have moved the sentence. We also worked to improve the clarity of the introduction as suggested by reviewer 2.

The gene "signatures" used in this study remain very superficial as now several studies have identified complex molecular signatures reflecting PERK, IRE1 or ATF6 activity, As such the authors should use such tools to validate their correlation. In particular the authors used the expression of EIF2AK3, ERN1 and ATF6 mRNA as relevant predictors which they are not. The use of Reactome for the selection of UPR genes should have been complemented with UPR target genes found in other reference databases (MGI, GO) to provide an exhaustive view of the pathway.

We hope to clarify that **Figure 3B** illustrates precisely the reviewers point, that the major regulators of the UPR branch pathways themselves are not very good biomarkers for UPR activity. Accordingly, we assembled gene sets downstream of each individual branch, including additional gene sets for RIDD and XBP1 arms of the IRE1a branch. **Figure 3C** shows that many of these downstream indicators of the UPR are activated by high levels of SCNAs and provide a better indication of UPR branch activities. We further used an approach similar to that used by [17], that seeks to score pathway activity based on the activities of downstream effector genes activated by those pathways. As suggested by this reviewer, we did seek to expand our pathway definitions from additional databases, however we note that including genes that are more peripherally associated with a pathway can also introduce noise.

The overlap between Reactome [18], MsigDB [19] and GO genesets [19,20] for each of the branch pathways is relatively high, with the most new genes being added for the PERK branch (**Figure R4**). Incorporating these additional genes increased the number of PERK pathway genes from 8 to 39 and the number of ATF6 pathway genes from 5 to 17. RIDD and XBP1 related genesets did not change.

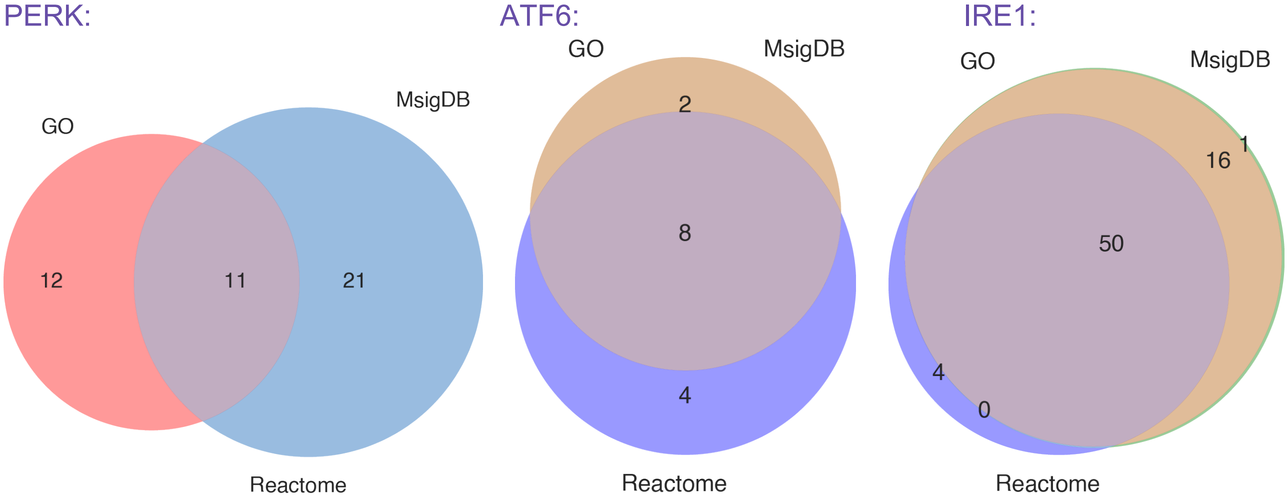

Figure R4. Correspondence between Reactome, GO and MsigDB gene sets describing the three UPR branch pathways.

We further evaluated whether including these additional genes improved UPR branch pathway scores by evaluating whether models with or without these genes were better at discriminating normal from tumor tissues. We evaluated the cases where including the extra genes increased or decreased the difference between tumor and normal pathway score distributions and found more cases where including the genes weakened the ability of the pathway biomarker to discriminate the two (**Figure R5**). Therefore, while we agree with the reviewer that

a set of confident downstream effector genes would be the best candidates for inclusion in a UPR branch activity biomarker, we do not think that the genes available through GO and MsigDb meet this criteria.

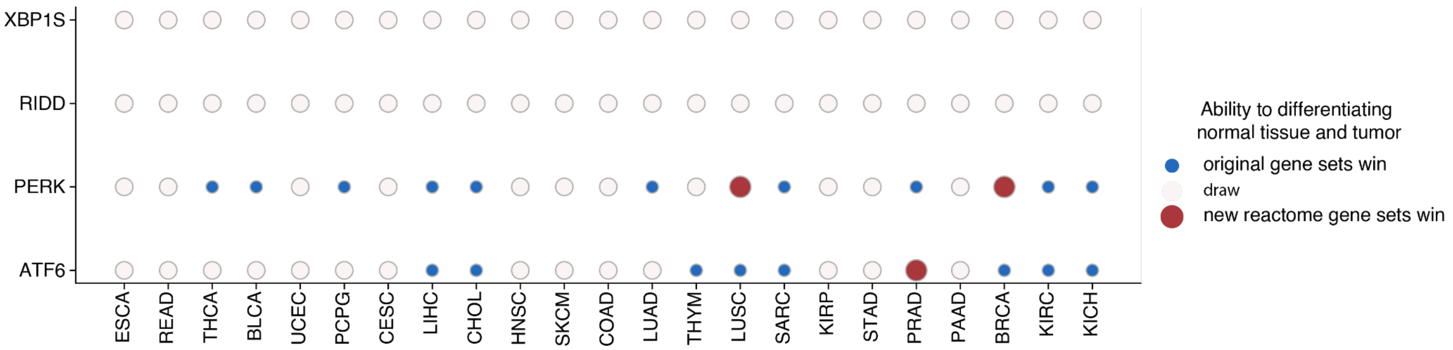

Figure R5. Comparison of models that predict tumor versus normal status based on UPR gene activity using original versus expanded genesets. In most cases, there is no significant difference in model performance, but in the majority of cases where we did see differences, the additional genes detracted from performance.

RIDD target genes used in the manuscript are those described by Maurel et al in 2014, however, even though this list constituted a first repository of mRNA found to be cleaved by IRE1 in various (and independent experimental settings), the authors should definitely refine this list for the purpose of their study. Many reports, since Maurel et al, identified new RIDD targets, and RIDD is very variable from one cell/tissue type to one other.

We sought to identify additional RIDD target sets in the literature. We identified two studies that are frequently cited [21,22] but these appear to predate the study by [23], and the three show limited agreement (**Figure R6**). A review just published in 2021 [24] suggests that only 37 genes have been confirmed as RIDD targets however did not provide a list of all genes. This suggests that we are not likely missing a large number of RIDD targets. In general, systematic identification of RIDD targets has been difficult because UPR activation generally employs multiple mechanisms of remodeling the transcriptome at the same time. Although a consensus cleavage sequence has been identified for RIDD, this sequence alone does not appear to effectively predict RIDD targets [25]. [26] investigated RIDD orthologs and found BLOC1S1 is a highly conserved RIDD target, and may serve as one of the best markers for RIDD activity [26].

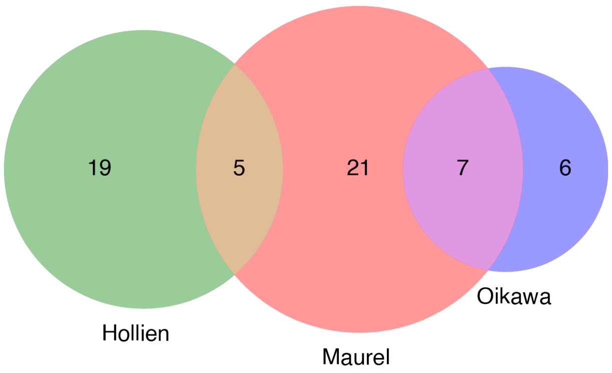

Figure R6: Overlap in RIDD target genes from three highly cited studies.

Figure 4D: I am not sure the annotations are correct, these should be checked again by the authors. For example, HERPUD1 is depicted in blue (PERK pathway) even though it is an ATF6 target.

We note that HERPUD1 is a member of the Reactome pathway “PERK regulates gene expression” (Id: R-HSA-381042.1) which may explain why we included it under PERK. We found several sources suggesting that

HERPUD1 is PERK dependent [27,28]. We now include it under both PERK and ATF6 for the various analyses in the manuscript.

How do the authors differentiate the ISR from the UPR, especially regarding the PERK pathway. For instance, what is the activation status of PKR or GCN2 in the tumors studied. This point should be specifically and experimentally addressed.

We thank the reviewer for asking us to interrogate the ISR in the context of aneuploidy. The question is whether in addition to the UPR aneuploidy also activates the ISR. Phosphorylation of eIF2α is the control regulatory hub of the ISR. Mechanistically, upon stress the alpha subunit of eIF2 is phosphorylated at Ser51 and interacts with eIF2B for which it displays strong affinity, acting as a potent noncompetitive inhibitor of it [29]. The ISR family is composed of four members of which PERK is one. The other three are double-stranded RNA-dependent protein kinase (PKR), general control non-repressible 2 (GCN2), and heme-regulated eIF2α kinase (HRI) [30]. Although all four eIF2α kinases share extensive homology in their kinase catalytic domains, each responds to distinct environmental and physiological stresses to reflect their unique regulatory mechanisms [31]. Thus, PKR responds to dsRNA during viral infections and GCN2 responds to amino acid deprivation and glucose deprivation. The experiments shown in the revision of the manuscript have been limited to the analysis of PKR and GCN2 considering it unnecessary to probe HRI, which is mainly expressed in erythroid cells.

As shown in the updated **Fig. 6G** neither kinase was phosphorylated in DLD1 cells treated with reversine as determined by Western blotting. A faint p-PKR band was observed in SKOV3 cells treated with reversine. Thapsigargin, which perturbs ER calcium homeostasis, did not induce the phosphorylation of either PKR or GCN2 as expected.

The new results, updated **Figure 6G**, indicate that aneuploidy induced by short-term treatment with reversine is mainly linked with inhibition of protein translation via eIF2α phosphorylation by a canonical UPR.

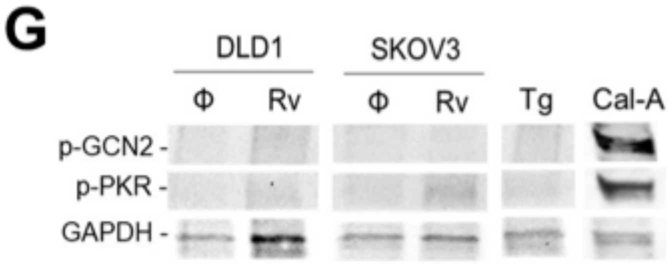

Updated Figure 6G.

The modest activation of the transcription factor ATF4 in reversine-treated cancer cells is consistent with lack of detectable activation of PKR and GCN2 since ATF4 is an important effector of the ISR [30]. Collectively, the new data suggest that aneuploid cells activate the UPR but not the ISR, two regulatory circuits of cellular homeostasis that share the common hub eIF2a. However, the GCN2-eIF2alpha-ATF4 pathway, which is critical for maintaining metabolic homeostasis in tumor cells [32] appears not to be activated by aneuploidy. Together with a reduced activation of ATF4 through the UPR, it appears as if aneuploid cells effectively avoid the deleterious consequences of ATF4 activation leading to apoptosis [33,34], to preserve survival.

Page 24. The authors state that "the fact that RIDD and PERK have a similar relationship to CYT is not surprising since RIDD activity was shown to be dependent (Moore and Hollien, 2015). How general is really this statement (does it apply to all RIDD substrates)? From the finding that the RIDD and PERK pathway score correlate in 8 out of 12 tumors, the authors conclude that "PERK and RIDD exert a negative effect on immune cells in the

tumor microenvironment cooperatively". This reads like an over-interpretation of this correlation and needs to be toned down here. RIDD activation was not associated with macrophage recruitment in brain tumor models, how do the authors reconcile their data with this observation?

We have modified the language to avoid over interpretation as follows:

“We found significant positive correlation in eight out of twelve tumor types (**Fig EV5C**) where both RIDD and PERK pathway scores were available, consistent with the possibility of functional interdependence [75]. Thus, we conclude that among the UPR branch pathways, PERK and RIDD likely exert a negative effect on immune cells in the tumor microenvironment.”

Figure 6: Is the PERK branch (what is the eif2a phosphorylation status?; what is the PKR activation status) also regulated in these conditions? Same question for the RIDD. Moreover the secretome produced by those cells should be determined quantitatively.

The issue of the factor(s) responsible for transcellular effects originating in cancer cells has been an effort by our laboratory which proved to be difficult and to date it still remains unresolved. We have determined that the activity contained in conditioned medium form ER stressed cancer cells is assignable to a lipophilic compound whose activity can be entirely separated in the organic phase from the cell-free conditioned media by ethyl acetate partitioning.

Serial preparative HPLC fractionation organic extract using a series of chromatographic techniques led to the potential identification of a fraction that is responsible for XBP1 splicing and transcriptional upregulation of Il-6 and Arg1. In parallel, a lipidomic analysis at LipidMaps (UCSD Lipidomic Core) showed that among a panel of 154 individual eicosanoids present in the conditioned medium of ER stressed cancer cells there exists a selective enrichment in some eicosanoids, including PGE2, PGA2, PGD1, 5-HETE and PGJ2 (**Figure R7**). While these eicosanoids, individually or collectively, could not account for all biologic features (XBP1 splicing and Il-6 and Arg1 transcriptional upregulation), it is our current believe that they complement the activity of the stress-transmitting lipophilic compound.

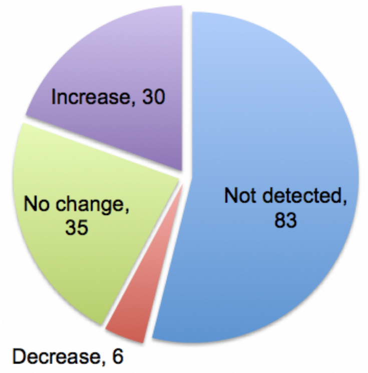

	Veh (nM)	TERS (nM)	Fold increase
PGA2	0.01	0.21	25.5
PGD1	0.49	2.59	5.29
PGE2	1.32	6.49	4.93
8-iso-15k PGF2b	0.95	4.33	4.57
PGJ2	0.01	0.03	3.59
11-HETE	0.17	0.62	3.57

Figure R7. Relevant lipids of the arachidonic acid family in the conditioned medium from ER stress cancer cells as detected by LipidMaps analysis. Abbreviations: Veh = vehicle medium; TERS = transmissible (transcellular) ER stress.

Figure 7: Is there also a direct effect of CM from aneuploid cells on T lymphocytes? Is there a role for the PERK branch here?

We thank the reviewer for the excellent suggestion that we broaden the scope of our studies and interrogate whether cell-nonautonomous effects of aneuploid cells on T cell activation. We performed the experiments that are now shown in updated **Fig. 7E-G**.

We activated human T cells from buffy coats of normal blood donors activated using a conventional method (anti-CD3 plus anti-CD28 Dynabeads). We found that the CM of aneuploid cells caused a marked decrease of both IFNγ and Granzyme B relative to control CM (**Figure 7E**). The effect could not be attributed to carry-over of Reversine since cultured medium spiked with Reversine (0.5 μM) had only modest effect (**Figure 7F**). IFNγ and Granzyme B were also reduced in human T cells treated with CM from fused B16 cells (**Figure 7G**).

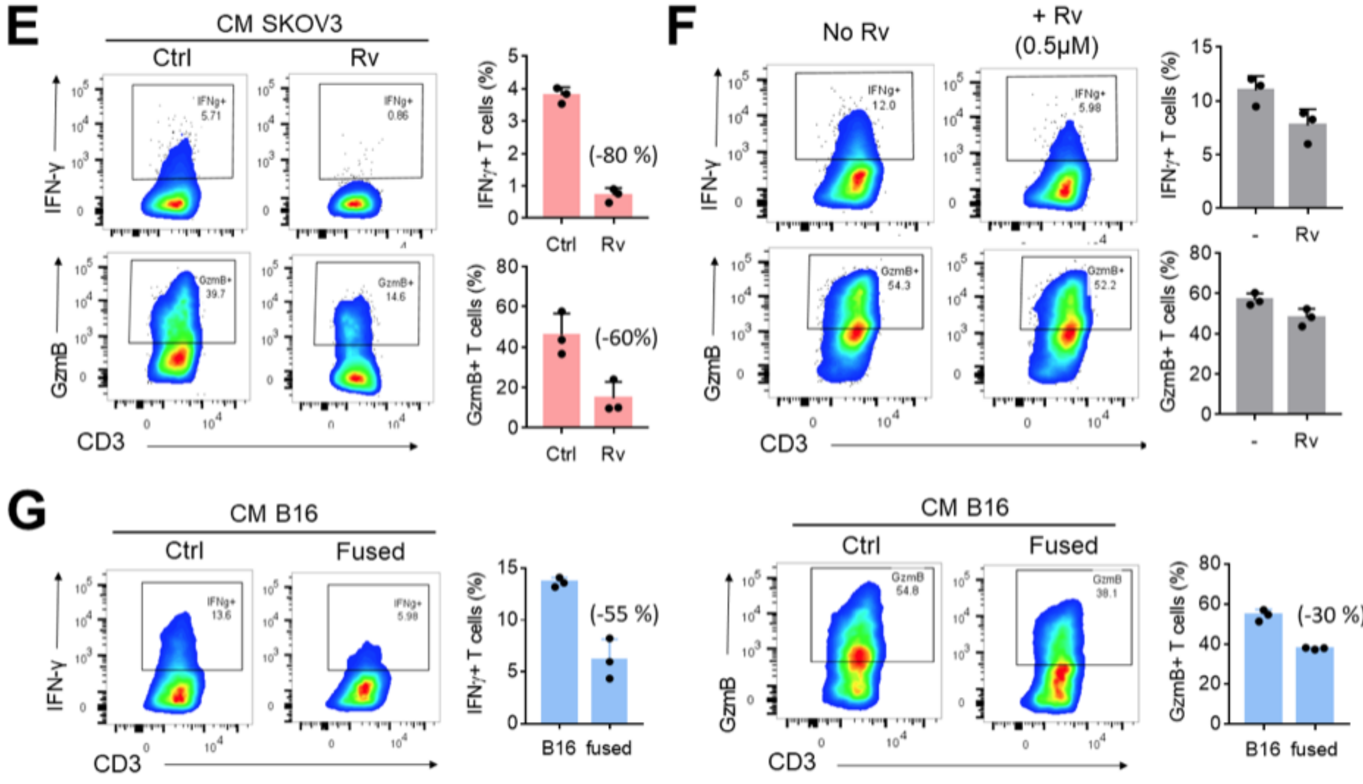

Is the activity of IRE1 (XBP1 splicing or RIDD) and/or PERK in the aneuploid cells required for the effect observed on BMDMs? RIDD activity should be quantified.

We note that our analysis suggested RIDD activity was stronger in infiltrating immune cells than tumor cells (**Fig EV5**).

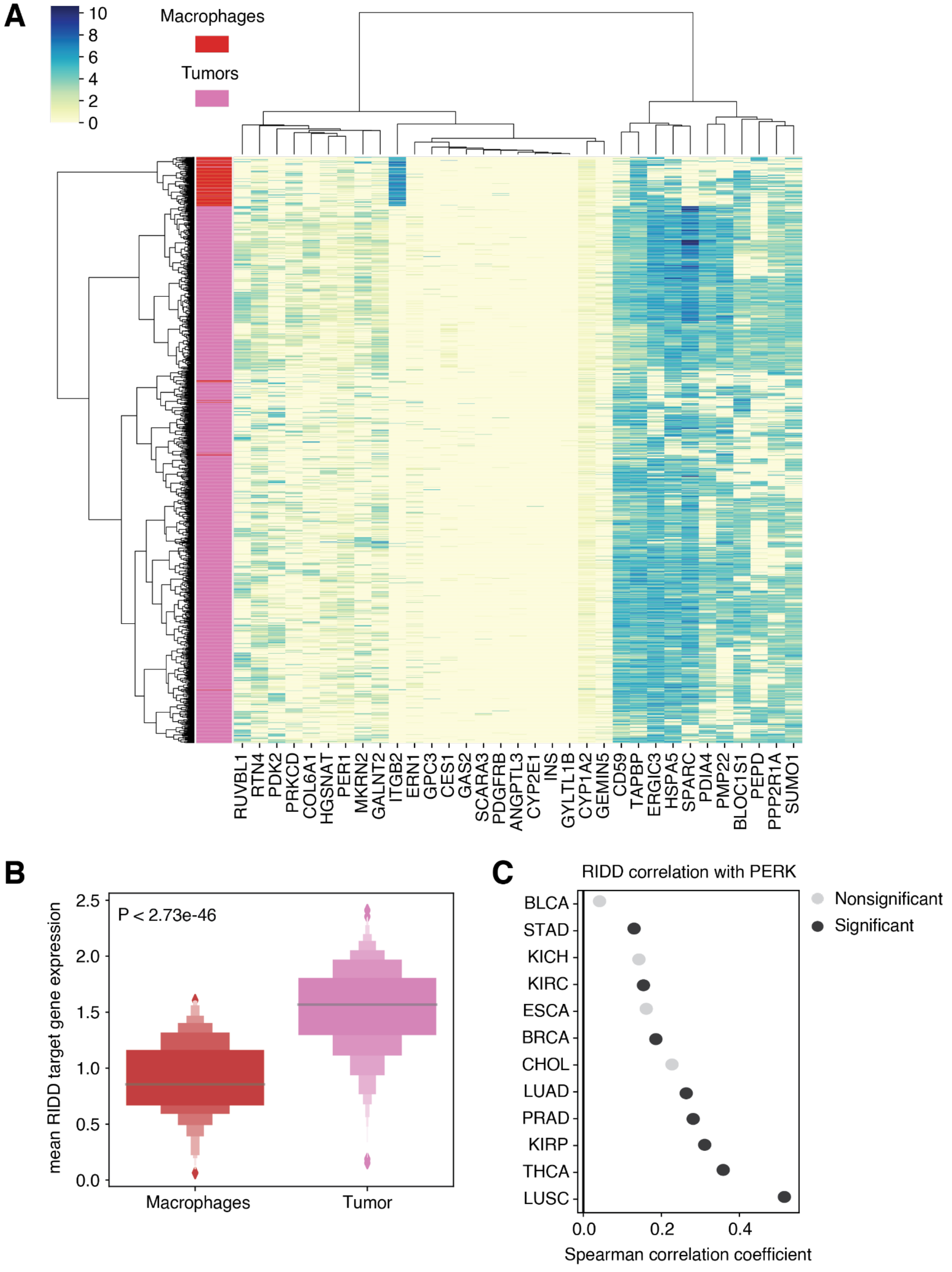

Figure EV5. Analysis of RIDD target gene expression in single-cell data showed reduced expression in tumor-associated macrophages compared to tumor cells.

(**A**) Heatmap showing the unsupervised clustering of 1257 tumor cells and 119 macrophages (rows) according to expression in $\log_2$(TPM/10 + 1) of 33 RIDD target genes (columns). The left sidebar indicates cell type: red – macrophages; pink – tumor cells.

(**B**) Boxplots showing the distribution of mean RIDD target gene expression in macrophages (blue) and tumor cells (orange). ITGB2 and TAPBP were excluded as their behavior is counter to regulation by RIDD.

(**C**) Spearman correlation coefficients linking PERK pathway score and RIDD activity score for 12 tumor types for which both pathway scores could be calculated. Black color indicates correlations that were statistically

significant after multiple hypothesis testing correction using the Benjamini Hochberg procedure (FDR < 0.05). Gray dots represent non-significant cases.

We nonetheless attempted to quantify RIDD activity in SKOV3 and DLD1 cells through PCR measurement of the prototype RIDD target gene BLOC1S1 per our recent publication [35]. In repeat assays we found a minor decrease in expression in SKOV3 cells at 72 hours and none at the 6 hr and time points in DLD1 cells (**Figure R8**).

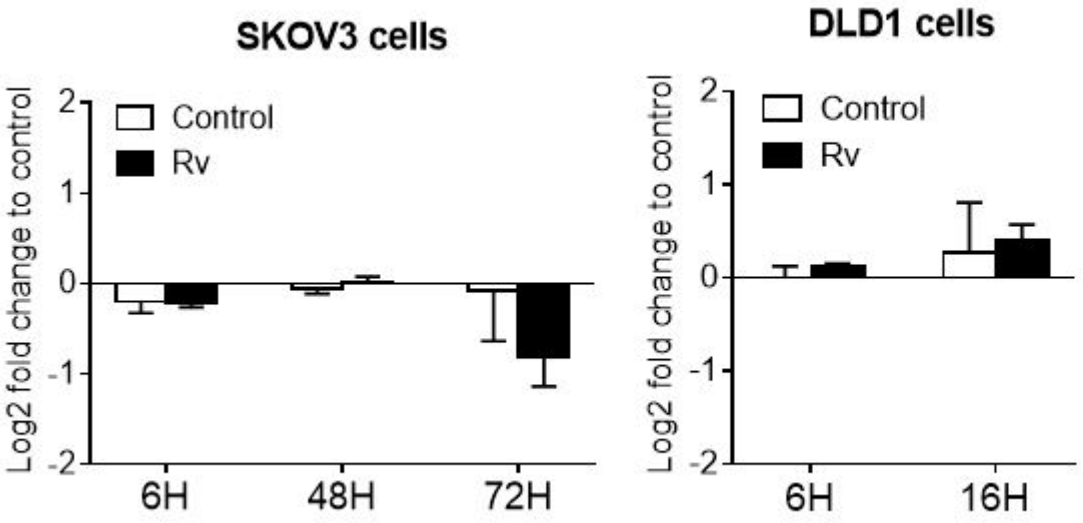

Figure R8. mRNA expression (RT-PCR) of BLOC1S1 in DLD1 and SKOV3 cells treated or not with Rv. Bars represent the mean of triplicates +/- SEM.

This variability in RIDD activity between the two cell lines is consistent with the variability from cell-type to cell-type that the reviewer alluded to.

The question raised by the reviewer is an interesting one. Since BLOC1S1 provided essentially no clues and **Fig. EV5** shows that RIDD activity is stronger in infiltrating immune cells than tumor cells, refer for completeness to a finding from recent study of our lab showing that macrophages treated with CM containing transmissible ER stress factor(s) (TERS) activate RIDD and that this activation is IRE1-dependent. For convenience of reviewers we show panels D and E of Fig. 5 of our paper [35], which was published June 10, 2020.

The experiment shows that macrophages treated with transmissible ER stress factor(s) undergo activation of RIDD that is lost in *Ern1*-/- macrophages. We believe that this analysis is sufficiently demonstrative of the fact that in macrophages RIDD is activated cell-nonautonomously.

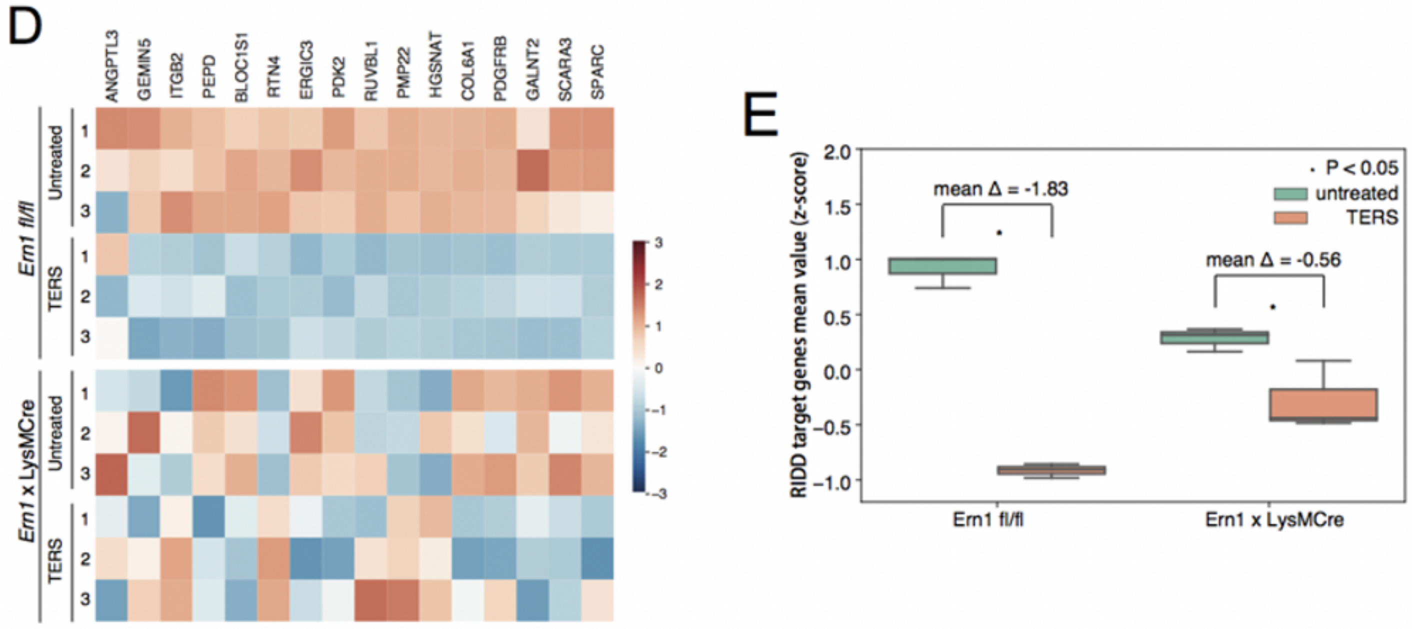

From [35] Figure 5 panels (D) Heatmap showing the relative expression of 16 RIDD target genes in untreated or TERS CM–treated WT or Ern1-CKO BMDM. (E) Comparison of mean z scores for the 16 RIDD target genes in untreated or TERS CM–treated WT or Ern1- CKO BMDM.

In the models tested, the authors should also differentiate aneuploidy-associated proteotoxicity from UPR signalling.

Differentiating aneuploidy-associated proteotoxicity from UPR is an excellent suggestion, albeit a difficult one from an experimental standpoint. We performed an experiment but it proved inconclusive. Briefly, with the assistance of Dr. Robert Signer (UCSD Moores Cancer Center) [36] we interrogated fused B16 cells that are highly aneuploid, measuring the abundance of ubiquitylated proteins according to [37]. The method is exemplified below.

From: A thiol probe for measuring unfolded protein load and proteostasis in cells

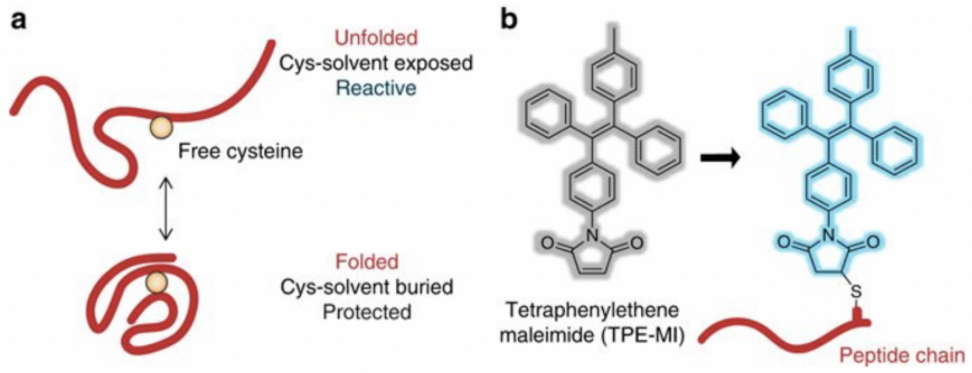

Strategy for assaying protein foldedness via access to buried cysteine thiols. **a** Strategy for probing free cysteine thiols that become exposed to the solvent upon protein unfolding and permissive to maleimide reaction. **b** Structure of tetraphenylethene (TPE) conjugated to a maleimide (MI). Fluorescence is enabled upon conjugation to a protein and immobilisation of the phenyl rotamers.

From [37] Figure 1 describing use of a thiol probe to assay proteostasis.

Using this technique we found no detectable levels of misfolded proteins in the fused B1 clone relative to parental B16 cells (Figure R9).

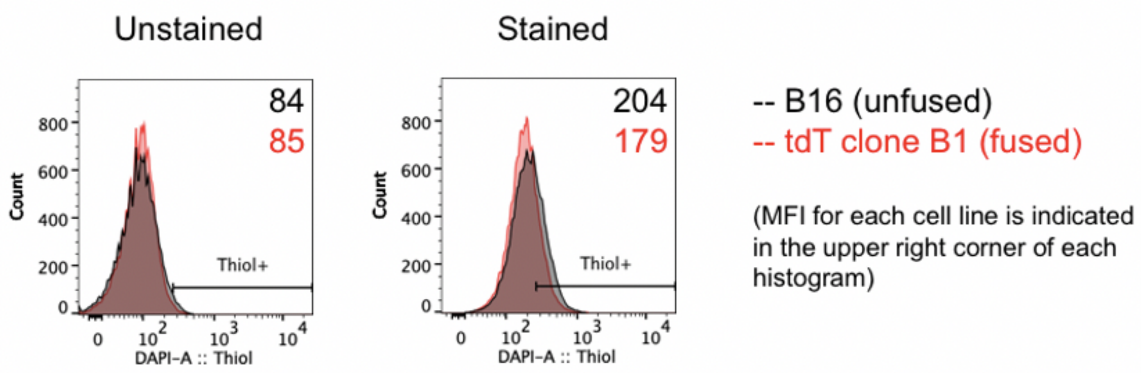

Figure R9. Comparison of measured unfolded proteins in unmanipulated B16 parental and B16 fused (clone #B1) cells.

Based on this result we abandoned this line of investigation on the belief that the method used may not be sensitive enough to detect variations in protein misfolding in aneuploid cells. Of note, the method was developed using purified proteins and confirmed under a variety of proteotoxic stresses but not in the context of aneuploidy.

In the literature, the connection between chromosome dosage changes, changes in mRNA and changes in protein levels have been established both in yeasts and eukaryotic cells [38]. In trisomic and tetrasomic eukaryotic cells, aneuploidy has been associated with a response pattern involving genes linked to endoplasmic reticulum, Golgi apparatus and lysosomes, and downregulation of DNA replication, transcription as well as ribosomes [39]. In these studies broad proteomic analysis were assessed by Stable Isotope Labeling with Amino acids in Cell culture (SILAC) [40].

A single recent report quantified the response to various canonical stressors (tunicamycin, thapsigargin and DTT) in HeLa cells using SILAC. This systematic quantification of the proteome-wide expression changes by proteostatic stress found variability among the different stressors and identified increased expression of 38 proteins not previously linked to the UPR [41].

In conclusion, at the present time the quantitative aspects of the relation between aneuploidy and misfolded protein content in cancer cells remains unanswered and may need a dedicated effort using higher sensitivity techniques.

In their models, what is the impact of antiproliferation compounds such as AICAR or 17AAG on the activation of the UPR and on the production of an immunomodulating secretome?

The reviewer asked that we test the effect of 17-AAG, an inhibitor of HSP90. Since 17-AAG has been shown to cause resistance to therapeutic agents [42], an effect imputed to the induction of the heat shock response and increased cellular levels of pro-survival chaperones [43], the question appears to be whether 17-AAG has an effect on the UPR when used in combination with reversine. In the experiment shown below, 17-AAG has both a direct effect and additive effect on the induction of UPR when used in combination with Reversine (**Figure R10**).

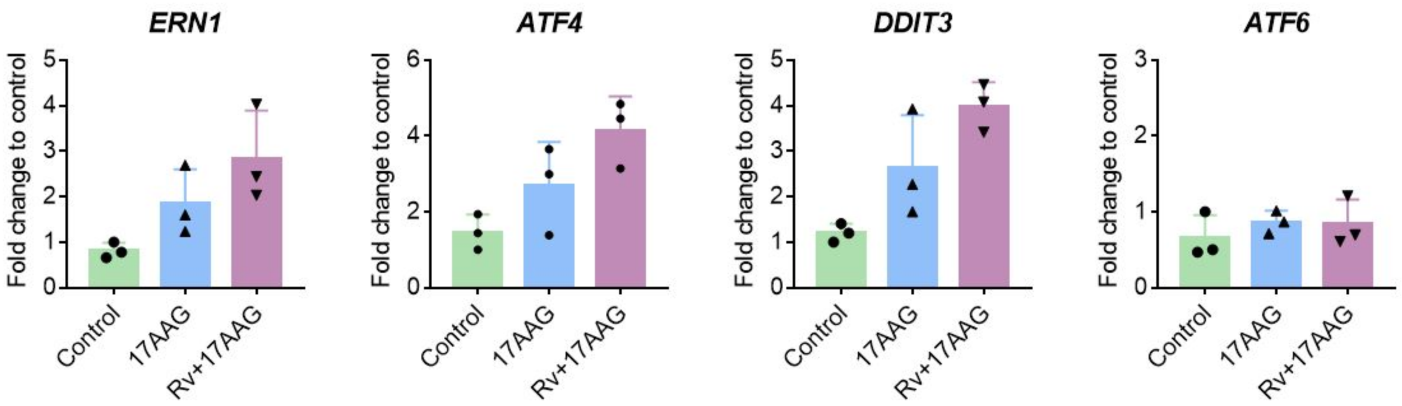

Figure R10. Effect of 17-AAG with and without Revesine on induction of the UPR.

This result suggests that 17-AAG may ultimately create a situation of adaptive UPR [13,44] favoring survival and drug resistance. We note that our result and interpretation may be at variance with a report by [45] showing that 17-AAG exerts anti-proliferative effects on MEFs trisomic for either Chromosome 1, 13, 16 or 19. In these experiments 17-AAG was used mainly in combination with AICAR, an analog of adenosine monophosphate (AMP) that is capable of stimulating AMP-dependent protein kinase (AMPK) activity, which is used clinically to treat and protect against cardiac ischemic injury.

At last, aneuploidy has been linked to resistance to chemotherapeutics in cancer cells, is that validated in the cancer data used by the authors? And if yes, how does the UPR relate to this phenotype?

The reviewer asked to assess the effect of reversine on the resistance to chemotherapy for which we used Paclitaxel. The protocol is as shown in **Figure R11** panel A. The result in panel B shows that pre-treatment with reversine does not modify the response of aneuploid cells to Paclitaxel (5µM) since no difference in the percentage of early or late apoptotic cells relative to control cells treated with Paclitaxel were observed in an Annexin-V assay.

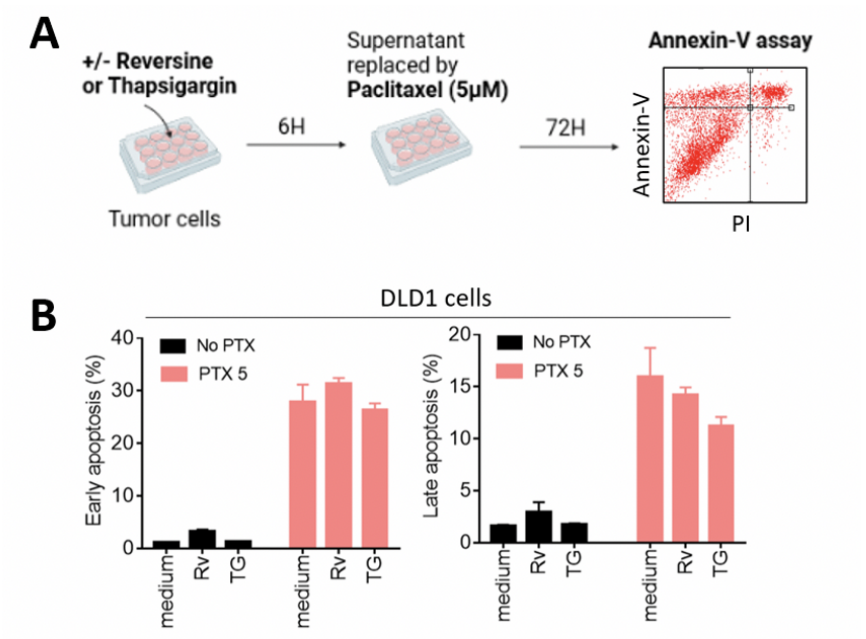

Figure R11. A) Protocol to assess combined effect of Reversine and Paclitaxel. B) Effect of pre-treatment with Reversine on Paclitaxel response as compared with control conditions quantified for early and late cell death.

We note that these results are from a report by [46] who showed that aneuploidy induces resistance to taxol.

Summary of Changes

To main text

1. We substantially revised the Introduction following the reviewers comments to (a) better logical progression of the arguments, and (b) more relevant citations.

2. We separated p53 and CYT results under two distinct subheadings for greater clarity.

To Figures

To facilitate the task of reviewers to identify the changes in primary figures we have prepared the Table shown below.

**Fig. 1**	No change in information. The display of panel D has been updated and reformatted.
**Fig. 2**	Information in panel C is new. We moved the original panel C to panel D.
**Fig. 3**	Panel B and C have been reformatted and provide similar representations but the underlying data were calculated differently in response to reviewer suggestions. The interpretation remains the same.
**Fig. 4**	Unchanged
**Fig. 5**	Panel D has been changed to address reviewer comments.
**Fig. 6**	Panels E-G are new. Portions of the data on fused B16 cells have been moved to Appendix Fig. S10.
**Fig. 7**	Panels E-G are new. Panels A-D have been updated.

In conclusion, the manuscript has been significantly improved and clarified by systematically addressing the reviewer comments and suggestions. The correlative aspect between aneuploidy and UPR, even after revision remains a foundational aspect of the paper. To the best of our knowledge this type of analysis has not been reported before. The paper offers in addition two new and relevant pieces of information: 1) aneuploidy triggers a more global UPR than previously assessed, and 2) T cells are affected in a cell non-autonomous way. This is very relevant to the biology of T cells in the tumor microenvironment and no doubt the beginning of new explorations. We feel, therefore, that our work makes an important advance toward understanding the interaction between tumor genome evolution and the tumor immune response.

References:

1. Auslander N, Wolf YI, Koonin EV (2020) Interplay between DNA damage repair and apoptosis shapes cancer evolution through aneuploidy and microsatellite instability. *Nat Commun* **11**: 1–11.
2. Shelly S, Agmon-Levin N, Altman A, Shoenfeld Y (2011) Thymoma and autoimmunity. *Cell Mol Immunol* **8**: 199–202.
3. Hoffacker V, Schultz A, Tiesinga JJ, Gold R, Schalke B, Nix W, Kiefer R, Müller-Hermelink HK, Marx A (2000) Thymomas alter the T-cell subset composition in the blood: a potential mechanism for thymoma-associated autoimmune disease. *Blood* **96**: 3872–3879.
4. Bando H, Iguchi G, Okimura Y, Odake Y, Yoshida K, Matsumoto R, Suda K, Nishizawa H, Fukuoka H, Mokubo A, et al. (2017) A novel thymoma-associated autoimmune disease: Anti-PIT-1 antibody syndrome. *Sci Rep* **7**: 43060.
5. Weenink B, Draaisma K, Ooi HZ, Kros JM, Sillevis Smitt PAE, Debets R, French PJ (2019) Low-grade glioma harbors few CD8 T cells, which is accompanied by decreased expression of chemo-attractants, not immunogenic antigens. *Sci Rep* **9**: 1–11.
6. Cheng W, Ren X, Zhang C, Cai J, Liu Y, Han S, Wu A (2016) Bioinformatic profiling identifies an immune-related risk signature for glioblastoma. *Neurology* **86**: 2226–2234.
7. Carter SL, Eklund AC, Kohane IS, Harris LN, Szallasi Z (2006) A signature of chromosomal instability inferred from gene expression profiles predicts clinical outcome in multiple human cancers. *Nat Genet* **38**: 1043–1048.
8. Bonneville R, Krook MA, Kautto EA, Miya J, Wing MR, Chen H-Z, Reeser JW, Yu L, Roychowdhury S (2017) Landscape of Microsatellite Instability Across 39 Cancer Types. *JCO Precis Oncol* **2017.**:
9. Nielsen T, Wittenborn T, Horsman MR (2012) Dynamic Contrast-Enhanced Magnetic Resonance Imaging (DCE-MRI) in Preclinical Studies of Antivascular Treatments. *Pharmaceutics* **4**: 563–589.
10. Larghi A, Capurso G, Carnuccio A, Ricci R, Alfieri S, Galasso D, Lugli F, Bianchi A, Panzuto F, De Marinis L, et al. (2012) Ki-67 grading of nonfunctioning pancreatic neuroendocrine tumors on histologic samples obtained by EUS-guided fine-needle tissue acquisition: a prospective study. *Gastrointest Endosc* **76**: 570–577.
11. DuRose JB, Tam AB, Niwa M (2006) Intrinsic capacities of molecular sensors of the unfolded protein response to sense alternate forms of endoplasmic reticulum stress. *Mol Biol Cell* **17**: 3095–3107.
12. Yang H, Niemeijer M, van de Water B, Beltman JB (2020) ATF6 Is a Critical Determinant of CHOP Dynamics during the Unfolded Protein Response. *iScience* **23**: 100860.
13. Rodvold JJ, Chiu KT, Hiramatsu N, Nussbacher JK, Galimberti V, Mahadevan NR, Willert K, Lin JH, Zanetti M (2017) Intercellular transmission of the unfolded protein response promotes survival and drug resistance in cancer cells. *Sci Signal* **10.**:
14. Spaan CN, Smit WL, van Lidth de Jeude JF, Meijer BJ, Muncan V, van den Brink GR, Heijmans J (2019) Expression of UPR effector proteins ATF6 and XBP1 reduce colorectal cancer cell proliferation and stemness by activating PERK signaling. *Cell Death Dis* **10**: 490.
15. Yoshida H, Matsui T, Yamamoto A, Okada T, Mori K (2001) XBP1 mRNA is induced by ATF6 and spliced by IRE1 in response to ER stress to produce a highly active transcription factor. *Cell* **107**: 881–891.
16. Soto M, Raaijmakers JA, Bakker B, Spierings DCJ, Lansdorp PM, Foijer F, Medema RH (2017) p53 Prohibits Propagation of Chromosome Segregation Errors that Produce Structural Aneuploidies. *Cell Rep* **19**: 2423–2431.
17. Schubert M, Klinger B, Klünemann M, Sieber A, Uhlitz F, Sauer S, Garnett MJ, Blüthgen N, Saez-Rodriguez J (2018) Perturbation-response genes reveal signaling footprints in cancer gene expression. *Nat Commun* **9**: 1–11.
18. Jassal B, Matthews L, Viteri G, Gong C, Lorente P, Fabregat A, Sidiropoulos K, Cook J, Gillespie M, Haw R, et al. (2020) The reactome pathway knowledgebase. *Nucleic Acids Res* **48**: D498–D503.
19. Liberzon A, Birger C, Thorvaldsdóttir H, Ghandi M, Mesirov JP, Tamayo P (2015) The Molecular Signatures Database (MSigDB) hallmark gene set collection. *Cell Syst* **1**: 417–425.
20. The Gene Ontology Consortium (2019) The Gene Ontology Resource: 20 years and still GOing strong. *Nucleic Acids Res* **47**: D330–D338.
21. Hollien J, Lin JH, Li H, Stevens N, Walter P, Weissman JS (2009) Regulated Ire1-dependent decay of messenger RNAs in mammalian cells. *J Cell Biol* **186**: 323–331.

22. Oikawa D, Tokuda M, Hosoda A, Iwawaki T (2010) Identification of a consensus element recognized and cleaved by IRE1 alpha. *Nucleic Acids Res* **38**: 6265–6273.
23. Maurel M, Chevet E, Tavernier J, Gerlo S (2014) Getting RIDD of RNA: IRE1 in cell fate regulation. *Trends Biochem Sci* **39**: 245–254.
24. (2021) The molecular mechanism and functional diversity of UPR signaling sensor IRE1. *Life Sci* **265**: 118740.
25. Moore K, Hollien J (2015) Ire1-mediated decay in mammalian cells relies on mRNA sequence, structure, and translational status. *Mol Biol Cell* **26**: 2873–2884.
26. Bright MD, Itzhak DN, Wardell CP, Morgan GJ, Davies FE (2015) Cleavage of BLOC1S1 mRNA by IRE1 Is Sequence Specific, Temporally Separate from XBP1 Splicing, and Dispensable for Cell Viability under Acute Endoplasmic Reticulum Stress. *Mol Cell Biol* **35**: 2186–2202.
27. Ma Y, Hendershot LM (2004) Herp Is Dually Regulated by Both the Endoplasmic Reticulum Stress-specific Branch of the Unfolded Protein Response and a Branch That Is Shared with Other Cellular Stress Pathways. *Journal of Biological Chemistry* **279**: 13792–13799.
28. Gonen N, Sabath N, Burge CB, Shalgi R (2019) Widespread PERK-dependent repression of ER targets in response to ER stress. *Sci Rep* **9**: 4330.
29. Costa-Mattioli M, Walter P (2020) The integrated stress response: From mechanism to disease. *Science* **368.**:
30. Pakos-Zebrucka K, Koryga I, Mnich K, Ljujic M, Samali A, Gorman AM (2016) The integrated stress response. *EMBO Rep* **17**: 1374–1395.
31. Donnelly N, Gorman AM, Gupta S, Samali A (2013) The eIF2α kinases: their structures and functions. *Cell Mol Life Sci* **70**: 3493–3511.
32. Ye J, Kumanova M, Hart LS, Sloane K, Zhang H, De Panis DN, Bobrovnikova-Marjon E, Diehl JA, Ron D, Koumenis C (2010) The GCN2-ATF4 pathway is critical for tumour cell survival and proliferation in response to nutrient deprivation. *EMBO J* **29**: 2082–2096.
33. Han J, Back SH, Hur J, Lin Y-H, Gildersleeve R, Shan J, Yuan CL, Krokowski D, Wang S, Hatzoglou M, et al. (2013) ER-stress-induced transcriptional regulation increases protein synthesis leading to cell death. *Nat Cell Biol* **15**: 481–490.
34. Hiramatsu N, Chiang K, Aivati C, Rodvold JJ, Lee J-M, Han J, Chea L, Zanetti M, Koo EH, Lin JH (2020) PERK-mediated induction of microRNA-483 disrupts cellular ATP homeostasis during the unfolded protein response. *J Biol Chem* **295**: 237–249.
35. Batista A, Rodvold JJ, Xian S, Searles SC, Lew A, Iwawaki T, Almanza G, Waller TC, Lin J, Jepsen K, et al. (2020) IRE1α regulates macrophage polarization, PD-L1 expression, and tumor survival. *PLoS Biol* **18**: e3000687.
36. Hidalgo San Jose L, Sunshine MJ, Dillingham CH, Chua BA, Kruta M, Hong Y, Hatters DM, Signer RAJ (2020) Modest Declines in Proteome Quality Impair Hematopoietic Stem Cell Self-Renewal. *Cell Rep* **30**: 69–80.e6.
37. Chen MZ, Moily NS, Bridgford JL, Wood RJ, Radwan M, Smith TA, Song Z, Tang BZ, Tilley L, Xu X, et al. (2017) A thiol probe for measuring unfolded protein load and proteostasis in cells. *Nat Commun* **8**: 474.
38. Chunduri NK, Storchová Z (2019) The diverse consequences of aneuploidy. *Nat Cell Biol* **21**: 54–62.
39. Dürrbaum M, Kuznetsova AY, Passerini V, Stingele S, Stoehr G, Storchová Z (2014) Unique features of the transcriptional response to model aneuploidy in human cells. *BMC Genomics* **15**: 139.
40. Stingele S, Stoehr G, Peplowska K, Cox J, Mann M, Storchova Z (2012) Global analysis of genome, transcriptome and proteome reveals the response to aneuploidy in human cells. *Mol Syst Biol* **8**: 608.
41. Itzhak DN, Sacco F, Nagaraj N, Tyanova S, Mann M, Murgia M (2019) SILAC-based quantitative proteomics using mass spectrometry quantifies endoplasmic reticulum stress in whole HeLa cells. *Dis Model Mech* **12.**:
42. Gaspar N, Sharp SY, Pacey S, Jones C, Walton M, Vassal G, Eccles S, Pearson A, Workman P (2009) Acquired resistance to 17-allylamino-17-demethoxygeldanamycin (17-AAG, tanespimycin) in glioblastoma cells. *Cancer Res* **69**: 1966–1975.
43. Schaefer S, Svenstrup TH, Guerra B (2017) The small-molecule kinase inhibitor D11 counteracts 17-AAG-mediated up-regulation of HSP70 in brain cancer cells. *PLoS One* **12**: e0177706.
44. Rutkowski DT, Arnold SM, Miller CN, Wu J, Li J, Gunnison KM, Mori K, Sadighi Akha AA, Raden D, Kaufman RJ (2006) Adaptation to ER stress is mediated by differential stabilities of pro-survival and pro-

apoptotic mRNAs and proteins. *PLoS Biol* **4**: e374.

45. Tang Y-C, Williams BR, Siegel JJ, Amon A (2011) Identification of aneuploidy-selective antiproliferation compounds. *Cell* **144**: 499–512.
46. Swanton C, Nicke B, Schuett M, Eklund AC, Ng C, Li Q, Hardcastle T, Lee A, Roy R, East P, et al. (2009) Chromosomal instability determines taxane response. *Proc Natl Acad Sci U S A* **106**: 8671–8676.

Dear Prof Zanetti

Thank you for the submission of your revised manuscript to EMBO reports. We have now received the full set of referee reports that is copied below.

As you can see, the referees find that the study is significantly improved during revision and recommend publication. Before I can accept the manuscript, I need you to address some editorial points below:

- Please reduce the number of keywords to 5.

- Please format the references according to the new alphabetical style of EMBO reports. The abbreviation 'et al' should be used if more than 10 authors. You can download the respective EndNote file from our Guide to Authors https://endnote.com/style_download/embo-reports/

- Please enter all information on funding, including grant numbers, also in the relevant fields in our online submission system.

- Please upload the figures as individual production quality files (.eps, .tif, .jpg; one file per figure). This also applies to EV figures. The figures should be removed from the manuscript text file.

- Please add callouts in the text to Fig. 1B, Fig. 4D and the panels of Fig. EV4.

- Please add the heading 'Expanded View Figure Legends' to these legends

- Appendix: please add page numbers to the table of content
- Appendix figure S10: Please add information on the number of technical or biological repeats and define the bars in (B).

- Please upload the EV tables in .xls or .xlsx format and add a legend to the first line of the file.
- Table EV4 would be best uploaded as a dataset excel file using the name 'Dataset EV1' and with the legend to a separate tab.

- Your methods section refers to the Appendix and Supplementary text for detailed experimental procedures. Please note that all Materials and Methods must be part of the main manuscript. Therefore, please add this information to the Materials and Methods section.
- Please also add a statement regarding the use of mice and the committee approving these experiments, even if it is just the isolation of bone marrow cells.

- Please change 'Data and materials availability' to 'Data availability' and move this section to the end of the Materials and Methods section.

- Figure legend for 7B, C indicates that the experiment was run in duplicate but the graphs display three datapoints. Please clarify.

- Finally, EMBO reports papers are accompanied online by A) a short (1-2 sentences) summary of the findings and their significance, B) 2-3 bullet points highlighting key results and C) a synopsis image that is 550x200-600 pixels large (width x height) in .png format. You can either show a model or key data in the synopsis image. Please note that the size is rather small and that text needs to be readable at the final size. Please send us this information along with the revised manuscript.

We look forward to seeing a final version of your manuscript as soon as possible.

With kind regards,

Martina Rembold, PhD
Senior Editor
EMBO reports

****

Referee #1:

Xian and colleagues have adequately revised the manuscript according to the comments from my first round of review. This manuscript is now suitable for publication in EMBO reports

Referee #2:

I would like to thank the authors for the work carried out to address my critique on the initial version of the manuscript, and which is entirely satisfactory. I have no further comment at this stage.

The authors have addressed all minor editorial requests.

Prof. Maurizio Zanetti
UCSD
United States

Dear Maurizio,

Thank you for clarifying some remaining points. I am now very pleased to accept your manuscript for publication in the next available issue of EMBO reports. Thank you for your contribution to our journal.

At the end of this email I include important information about how to proceed. Please ensure that you take the time to read the information and complete and return the necessary forms to allow us to publish your manuscript as quickly as possible.

As part of the EMBO publication's Transparent Editorial Process, EMBO reports publishes online a Review Process File to accompany accepted manuscripts. As you are aware, this File will be published in conjunction with your paper and will include the referee reports, your point-by-point response and all pertinent correspondence relating to the manuscript.

If you do NOT want this File to be published, please inform the editorial office within 2 days, if you have not done so already, otherwise the File will be published by default [contact: emboreports@embo.org]. If you do opt out, the Review Process File link will point to the following statement: "No Review Process File is available with this article, as the authors have chosen not to make the review process public in this case."

Thank you again for your contribution to EMBO reports and congratulations on a successful publication. Please consider us again in the future for your most exciting work.

Kind regards,

Martina

Martina Rembold, PhD
Senior Editor
EMBO reports

****

THINGS TO DO NOW:

You will receive proofs by e-mail approximately 2-3 weeks after all relevant files have been sent to our Production Office; you should return your corrections within 2 days of receiving the proofs.

Please inform us if there is likely to be any difficulty in reaching you at the above address at that time. Failure to meet our deadlines may result in a delay of publication, or publication without your corrections.

All further communications concerning your paper should quote reference number EMBOR-2021-52509V4 and be addressed to emboreports@wiley.com.

Should you be planning a Press Release on your article, please get in contact with emboreports@wiley.com as early as possible, in order to coordinate publication and release dates.

EMBO PRESS

YOU MUST COMPLETE ALL CELLS WITH A PINK BACKGROUND ➔

PLEASE NOTE THAT THIS CHECKLIST WILL BE PUBLISHED ALONGSIDE YOUR PAPER

Corresponding Author Name: Maurizio Zanetti and Hannah Carter
Journal Submitted to: EMBO Reports
Manuscript Number: EMBOR-2021-52509V2

USEFUL LINKS FOR COMPLETING THIS FORM

http://www.antibodypedia.com
http://1degreebio.org
http://www.equator-network.org/reporting-guidelines/improving-bioscience-research-repor
http://grants.nih.gov/grants/olaw/olaw.htm
http://www.mrc.ac.uk/Ourresearch/Ethicsresearchguidance/Useofanimals/index.htm
http://ClinicalTrials.gov
http://www.consort-statement.org
http://www.consort-statement.org/checklists/view/32-consort/66-title
http://www.equator-network.org/reporting-guidelines/reporting-recommendations-for-tumo
http://datadryad.org
http://figshare.com
http://www.ncbi.nlm.nih.gov/gap
http://www.ebi.ac.uk/ega
http://biomodels.net/
http://biomodels.net/miriam/
http://jjj.biochem.sun.ac.za
https://osp.od.nih.gov/biosafety-biosecurity-and-emerging-biotechnology/
http://www.selectagents.gov/

Reporting Checklist For Life Sciences Articles (Rev. June 2017)

This checklist is used to ensure good reporting standards and to improve the reproducibility of published results. These guidelines are consistent with the Principles and Guidelines for Reporting Preclinical Research issued by the NIH in 2014. Please follow the journal's authorship guidelines in preparing your manuscript.

A- Figures
1. Data
The data shown in figures should satisfy the following conditions:

- ➔ the data were obtained and processed according to the field's best practice and are presented to reflect the results of the experiments in an accurate and unbiased manner.
- ➔ figure panels include only data points, measurements or observations that can be compared to each other in a scientifically meaningful way.
- ➔ graphs include clearly labeled error bars for independent experiments and sample sizes. Unless justified, error bars should not be shown for technical replicates.
- ➔ if n< 5, the individual data points from each experiment should be plotted and any statistical test employed should be justified
- ➔ Source Data should be included to report the data underlying graphs. Please follow the guidelines set out in the author ship guidelines on Data Presentation.

2. Captions

Each figure caption should contain the following information, for each panel where they are relevant:

- ➔ a specification of the experimental system investigated (eg cell line, species name).
- ➔ the assay(s) and method(s) used to carry out the reported observations and measurements
- ➔ an explicit mention of the biological and chemical entity(ies) that are being measured.
- ➔ an explicit mention of the biological and chemical entity(ies) that are altered/varied/perturbed in a controlled manner.

- ➔ the exact sample size (n) for each experimental group/condition, given as a number, not a range;
- ➔ a description of the sample collection allowing the reader to understand whether the samples represent technical or biological replicates (including how many animals, litters, cultures, etc.).
- ➔ a statement of how many times the experiment shown was independently replicated in the laboratory.
- ➔ definitions of statistical methods and measures:
  - common tests, such as t-test (please specify whether paired vs. unpaired), simple χ^2 tests, Wilcoxon and Mann-Whitney tests, can be unambiguously identified by name only, but more complex techniques should be described in the methods section;
  - are tests one-sided or two-sided?
  - are there adjustments for multiple comparisons?
  - exact statistical test results, e.g., P values = x but not P values < x;
  - definition of 'center values' as median or average;
  - definition of error bars as s.d. or s.e.m.

Any descriptions too long for the figure legend should be included in the methods section and/or with the source data.

In the pink boxes below, please ensure that the answers to the following questions are reported in the manuscript itself. Every question should be answered. If the question is not relevant to your research, please write NA (non applicable). We encourage you to include a specific subsection in the methods section for statistics, reagents, animal models and human subjects.

B- Statistics and general methods

Please fill out these boxes ➔ (Do not worry if you cannot see all your text once you press return)

1.a. How was the sample size chosen to ensure adequate power to detect a pre-specified effect size?	We used the largest publicly available data set of human multi-omic tumor data, which comprises > 9000 samples. Wherever possible, we performed pan-cancer analysis and included tumor as a covariate. In these cases, we made sure to clarify sample sizes and charactieristics of the underlying data, and always performed multiple hypothesis testing. For experiments, a minimum of 3 biological replicates was required.
1.b. For animal studies, include a statement about sample size estimate even if no statistical methods were used.	There are no in vivo animal experiemts per se. Mice were simply used as bone marrow donors.
2. Describe inclusion/exclusion criteria if samples or animals were excluded from the analysis. Were the criteria pre-established?	N/A
3. Were any steps taken to minimize the effects of subjective bias when allocating animals/samples to treatment (e.g. randomization procedure)? If yes, please describe.	N/A
For animal studies, include a statement about randomization even if no randomization was used.	N/A
4.a. Were any steps taken to minimize the effects of subjective bias during group allocation or/and when assessing results (e.g. blinding of the investigator)? If yes please describe.	N/A
4.b. For animal studies, include a statement about blinding even if no blinding was done	N/A
5. For every figure, are statistical tests justified as appropriate?	Statistical analysis has been performed and p values indicated wherever applicable
Do the data meet the assumptions of the tests (e.g., normal distribution)? Describe any methods used to assess it.	We used standard methods for processing and normalization of the data types we evaluated, and well established statistical approaches for those data types. We visualized data distributions to ensure they were approximately normal where appropriate.

Is there an estimate of variation within each group of data?	In addition to showing how data are distributed (e.g. Figure 1D) we also show tumor-type specific variance in SCNA levels in Figure 3C.
Is the variance similar between the groups that are being statistically compared?	We do not do a direct statistical comparison of groups with different variances with any statistical test that assumes similar variance. The statistical approaches we use allow for different variances in different groups, for example multivariable regression using a covariates to indicate tumor type.

C- Reagents

6. To show that antibodies were profiled for use in the system under study (assay and species), provide a citation, catalog number and/or clone number, supplementary information or reference to an antibody validation profile. e.g., Antibodypedia (see link list at top right), 1DegreeBio (see link list at top right).	This information is provided in Supplemenray Informatioion Material and Methods
7. Identify the source of cell lines and report if they were recently authenticated (e.g., by STR profiling) and tested for mycoplasma contamination.	This infrmation for both human and murine cell lines is provided in Supplemenray Informatioion Material and Methods

* for all hyperlinks, please see the table at the top right of the document

D- Animal Models

8. Report species, strain, gender, age of animals and genetic modification status where applicable. Please detail housing and husbandry conditions and the source of animals.	Bone marrow donor mice were housed in the UCSD vivarium. Femurs were removed per UCSD IACUC approved protocol in compliance with animal welfare standards.
9. For experiments involving live vertebrates, include a statement of compliance with ethical regulations and identify the committee(s) approving the experiments.	Human blood was purchased from the San Diego Blood Bank. No IRB is required for the purchase of esisting tissue de-identified.
10. We recommend consulting the ARRIVE guidelines (see link list at top right) (PLoS Biol. 8(6), e1000412, 2010) to ensure that other relevant aspects of animal studies are adequately reported. See author guidelines, under 'Reporting Guidelines'. See also: NIH (see link list at top right) and MRC (see link list at top right) recommendations. Please confirm compliance.	N/A

E- Human Subjects

11. Identify the committee(s) approving the study protocol.	No ethical committee was required to apporve this study
12. Include a statement confirming that informed consent was obtained from all subjects and that the experiments conformed to the principles set out in the WMA Declaration of Helsinki and the Department of Health and Human Services Belmont Report.	N/A
13. For publication of patient photos, include a statement confirming that consent to publish was obtained.	N/A
14. Report any restrictions on the availability (and/or on the use) of human data or samples.	N/A
15. Report the clinical trial registration number (at ClinicalTrials.gov or equivalent), where applicable.	N/A
16. For phase II and III randomized controlled trials, please refer to the CONSORT flow diagram (see link list at top right) and submit the CONSORT checklist (see link list at top right) with your submission. See author guidelines, under 'Reporting Guidelines'. Please confirm you have submitted this list.	N/A
17. For tumor marker prognostic studies, we recommend that you follow the REMARK reporting guidelines (see link list at top right). See author guidelines, under 'Reporting Guidelines'. Please confirm you have followed these guidelines.	N/A

F- Data Accessibility

18: Provide a "Data Availability" section at the end of the Materials & Methods, listing the accession codes for data generated in this study and deposited in a public database (e.g. RNA-Seq data: Gene Expression Omnibus GSE39462, Proteomics data: PRIDE PXD000208 etc.) Please refer to our author guidelines for 'Data Deposition'.<br><br>Data deposition in a public repository is mandatory for:<br>a. Protein, DNA and RNA sequences<br>b. Macromolecular structures<br>c. Crystallographic data for small molecules<br>d. Functional genomics data<br>e. Proteomics and molecular interactions	We provide a link to a github repository with code and data to allow all computational analyses to be reproduced.
19. Deposition is strongly recommended for any datasets that are central and integral to the study; please consider the journal's data policy. If no structured public repository exists for a given data type, we encourage the provision of datasets in the manuscript as a Supplementary Document (see author guidelines under 'Expanded View' or in unstructured repositories such as Dryad (see link list at top right) or Figshare (see link list at top right).	N/A
20. Access to human clinical and genomic datasets should be provided with as few restrictions as possible while respecting ethical obligations to the patients and relevant medical and legal issues. If practically possible and compatible with the individual consent agreement used in the study, such data should be deposited in one of the major public access-controlled repositories such as dbGAP (see link list at top right) or EGA (see link list at top right).	We used only publicly available data and provided information on where the data can be obtained.
21. Computational models that are central and integral to a study should be shared without restrictions and provided in a machine-readable form. The relevant accession numbers or links should be provided. When possible, standardized format (SBML, CellML) should be used instead of scripts (e.g. MATLAB). Authors are strongly encouraged to follow the MIRIAM guidelines (see link list at top right) and deposit their model in a public database such as Biomodels (see link list at top right) or JWS Online (see link list at top right). If computer source code is provided with the paper, it should be deposited in a public repository or included in supplementary information.	Code is made available via a github repository.

G- Dual use research of concern

22. Could your study fall under dual use research restrictions? Please check biosecurity documents (see link list at top right) and list of select agents and toxins (APHIS/CDC) (see link list at top right). According to our biosecurity guidelines, provide a statement only if it could.	N/A
